# Atezolizumab Plus Bevacizumab as First-line Treatment for Patients With Metastatic Nonsquamous Non–Small Cell Lung Cancer With High Tumor Mutation Burden: A Nonrandomized Controlled Trial

**DOI:** 10.1001/jamaoncol.2022.5959

**Published:** 2022-12-15

**Authors:** Mariano Provencio, Ana Laura Ortega, Juan Coves-Sarto, Virginia Calvo, Raquel Marsé-Fabregat, Manuel Dómine, María Guirado, Enric Carcereny, Natalia Fernández, Ruth Álvarez, Remei Blanco, Luis León-Mateos, José Miguel Sánchez-Torres, Ivana Gabriela Sullivan, Manuel Cobo, Alfredo Sánchez-Hernández, Bartomeu Massuti, Belen Sierra-Rodero, Cristina Mártinez-Toledo, Roberto Serna-Blasco, Atocha Romero, Alberto Cruz-Bermúdez

**Affiliations:** 1 Medical Oncology Department, Hospital Universitario Puerta de Hierro-Majadahonda, Madrid, Spain; 2 Medical Oncology Department, Hospital Universitario de Jaén, Jaén, Spain; 3 Medical Oncology Department, Hospital Universitari Son Llàtzer, Palma de Mallorca, Spain; 4 Medical Oncology Department, Hospital Universitari Son Espases, Palma de Mallorca, Spain; 5 Cancer Research Area, Instituto de Investigación Sanitaria, Fundación Jiménez Díaz, Madrid, Spain; 6 Medical Oncology Department, Hospital General Universitario de Elche General de Elche, Elche, Spain; 7 Medical Oncology Department, Catalan Institute of Oncology, Hospital Universitari Germans Trias i Pujol, Badalona Applied Research Group in Oncology, Germans Trias i Pujol Research Institute, Badalona, Spain; 8 Medical Oncology Department, Hospital Universitario Lucus Augusti, Lugo, Spain; 9 Hospital Vírgen de la Salud, Toledo, Spain; 10 Consorci Sanitari de Terrassa, Terrassa, Spain; 11 Hospital Clínico Universitario de Santiago, Santiago de Compostela, Spain; 12 Hospital Universitario de La Princesa, Madrid, Spain; 13 Medical Oncology Department, Hospital de la Santa Creu i Sant Pau, Barcelona, Spain; 14 Hospital Universitario Regional de Málaga, Málaga, Spain; 15 Consorci Hospitalari Provincial de Castelló, Castelló de la Plana, Castelló, Spain; 16 Medical Oncology Department, Hospital General Universitario de Elche, Alicante, Spain

## Abstract

**Importance:**

Antiangiogenic drug combinations with anti–programmed cell death 1 protein and anti–programmed cell death 1 ligand 1 (PD-L1) agents are a novel treatment option for lung cancer. However, survival remains limited, and the activity of these combinations for tumors with high tumor mutation burden (TMB) is unknown.

**Objective:**

To assess the clinical benefits and safety of atezolizumab plus bevacizumab for patients with high-TMB advanced nonsquamous non–small cell lung cancer (NSCLC).

**Design, Setting, and Participants:**

This multicenter, single-arm, open-label, phase 2 nonrandomized controlled trial (Atezolizumab Plus Bevacizumab in First-Line NSCLC Patients [TELMA]) included treatment-naive patients aged 18 years or older with confirmed stage IIIB-IV nonsquamous NSCLC with TMB of 10 or more mutations/megabase and no *EGFR*, *ALK*, *STK11*, *MDM2,* or *ROS1* alterations. From May 2019 through January 2021, patients were assessed at 13 sites in Spain, with follow-up until February 28, 2022.

**Interventions:**

Participants were given atezolizumab, 1200 mg, plus bevacizumab, 15 mg/kg, on day 1 of each 21-day cycle. Treatment was continued until documented disease progression, unacceptable toxic effects, patient withdrawal, investigator decision, or death.

**Main Outcomes and Measures:**

The primary end point was 12-month progression-free survival (PFS) rate (according to Response Evaluation Criteria in Solid Tumours, version 1.1 criteria); PFS was defined as the time from enrollment to disease progression or death. Adverse events were monitored according to the National Cancer Institute Common Terminology Criteria for Adverse Events, version 4.0.

**Results:**

A total of 307 patients were assessed for trial eligibility, of whom 266 were ineligible for enrollment. Of the 41 patients enrolled, 3 did not fulfill all inclusion criteria and were excluded. The remaining 38 patients (28 [73.7%] male; mean [SD] age, 63.7 [8.3] years) constituted the per-protocol population. The 12-month PFS rate was 51.3% (95% CI, 34.2%-66.0%), which met the primary end point. The 12-month overall survival (OS) rate was 72.0% (95% CI, 54.1%-83.9%). The median PFS was 13.0 months (95% CI, 7.9-18.0 months), and the median OS was not reached. Of the 38 patients, 16 (42.1%) achieved an objective response and 30 (78.9%) achieved disease control. The median time to response was 2.8 months (IQR, 2.8-3.58 months), with a median duration of response of 11.7 months (range, 3.57-22.4 months; the response was ongoing at cutoff). Of 16 responses, 8 (50.0%) were ongoing. Most adverse events were grade 1 or 2. For atezolizumab, the most common adverse events were fatigue (6 [15.8%]) and pruritus (6 [15.8%]). For bevacizumab, they were hypertension (10 [26.3%]) and proteinuria (4 [10.5%]). Drug discontinuation occurred in 2 patients receiving atezolizumab (5.3%) and 3 patients receiving bevacizumab (7.9%). PD-L1 levels were not associated with response, PFS, or OS.

**Conclusions and Relevance:**

These findings suggest that atezolizumab with bevacizumab is a potential treatment for high-TMB nonsquamous NSCLC.

**Trial Registration:**

ClinicalTrials.gov Identifier: NCT03836066

## Key Points

**Question:**

What are the outcomes of atezolizumab plus bevacizumab for treatment of patients with advanced nonsquamous non–small cell lung cancer (NSCLC) with high tumor mutation burden?

**Findings:**

This multicenter, single-arm, open-label, phase 2 nonrandomized controlled trial including 38 adults found a favorable safety profile of atezolizumab plus bevacizumab, with 51.3% progression-free survival at 12 months and durable responses for patients with advanced nonsquamous NSCLC with high tumor mutation burden and no *EGFR* or *ALK* genomic alterations.

**Meaning:**

The findings suggest that atezolizumab plus bevacizumab could become a standard treatment in this patient population.

## Teaser

This nonrandomized controlled trial evaluates the clinical benefits and safety of atezolizumab plus bevacizumab for treatment of patients with metastatic non–small cell lung cancer (NSCLC) with high tumor mutation burden.

## Introduction

Frontline treatment options for patients with advanced or metastatic non–small cell lung cancer (NSCLC) have changed radically with the incorporation of immunotherapy into treatment algorithms.^1^ Immune checkpoint inhibitors targeting programmed cell death 1 protein (PD-1; eg, pembrolizumab and nivolumab), programmed cell death 1 ligand 1 (PD-L1; eg, atezolizumab), and cytotoxic T lymphocyte–associated antigen 4 (eg, ipilimumab), either as monotherapy or combined with chemotherapy, modify the tumor microenvironment and have emerged as a new standard of care for patients without actionable driver sequence variations.^2,3,4,5,6,7,8,9^ However, only a minority of tumors respond, and long-term survival for most patients remains poor. Atezolizumab has been approved as monotherapy for first-line treatment of patients with metastatic NSCLC whose tumors have high PD-L1 expression (either ≥50% of tumor cells or ≥10% of tumor-infiltrating immune cells) and no *EGFR* alteration or *ALK* translocation.^7,10^

Pathological angiogenesis caused by proangiogenic factors such as vascular endothelial growth factor (VEGF) prevents immune cells from infiltrating tumors efficiently, favoring resistance to immune checkpoint blockade.^11^ The use of antiangiogenic drugs can reprogram the tumor microenvironment, increasing the effectiveness of immunotherapy.^12,13,14^ Based on the results from the open-label phase 3 Impower150 trial,^8,15^ atezolizumab in combination with the humanized anti–VEGF-A monoclonal antibody bevacizumab plus carboplatin and paclitaxel has also been approved for the first-line treatment of patients with metastatic nonsquamous NSCLC regardless of PD-L1 expression.

Identifying predictive biomarkers for patient selection beyond PD-L1, which has limitations, particularly when immune checkpoint inhibitors are given in combination, is one of the critical challenges in immuno-oncology. Tumor mutation burden (TMB), a measure of the total amount of somatic coding sequence variations in a tumor that may function as neoantigens recognized by the immune system, has recently emerged as a promising biomarker.^16,17^ In NSCLC, PD-L1 and TMB have been found to be independent biomarkers.^18,19,20^ In general, patients with cancer with high TMB (≥10 mutations/megabase [mut/Mb] in tissue samples or ≥16 mut/Mb in blood samples measured by the FoundationOne CDx gene panel [Foundation Medicine]) are more likely to show improved objective response, durable benefit, and progression-free survival (PFS) from immune checkpoint blockade.^21,22^

We report the results of a single-arm, open-label, phase 2 nonrandomized controlled trial (Atezolizumab Plus Bevacizumab in First-Line NSCLC Patients [TELMA]) that evaluated the efficacy of atezolizumab in combination with bevacizumab as first-line treatment for patients with locally advanced or metastatic nonsquamous NSCLC with high TMB (≥10 mut/Mb or ≥16 mut/mB in tissue or blood samples, respectively) and no *EGFR* or *ALK* alterations. The primary efficacy end point was the rate of PFS at 12 months.

## Methods

### Study Design and Patients

TELMA is a multicenter, open-label, single-arm, phase 2 nonrandomized controlled trial (NCT03836066). Patients were eligible for the study if they were aged 18 years or older and had histologically or cytologically confirmed, treatment-naive, stage IIIB-IV nonsquamous NSCLC according to the International Association for the Study of Lung Cancer *Staging Manual in Thoracic Oncology, 8th Edition*^23,24^; measurable disease at baseline according to the Response Evaluation Criteria in Solid Tumours, version 1.1 (RECIST v1.1)^25^; a baseline Eastern Cooperative Oncology Group (ECOG) performance status of 0 or 1; adequate hematologic and organ function; and a high-intermediate TMB, defined as 10 mut/Mb or more when determined on archival tumor tissue samples or tissue samples obtained through biopsy at prescreening using the US Food & Drug Administration–approved FoundationOne CDx assay or as 16 mut/Mb or more when measured on circulating tumor DNA (ctDNA) in blood samples. Patients were excluded if they had known genomic alterations in *EGFR*, *ALK*, *STK11*/*LKB1*, *MDM2*, or *ROS1* genes; autoimmune disease; or active or untreated central nervous system metastases. Full details of the inclusion and exclusion criteria are listed in the trial protocol in Supplement 1 and the eResults in Supplement 2. This study was performed in accordance with the International Conference on Harmonization Good Clinical Practice guideline^26^ and the Declaration of Helsinki.^27^ All patients provided written informed consent before enrollment, and the protocol was approved by the clinical research ethics committee of the Hospital Puerta de Hierro-Majadahonda. This study followed the Transparent Reporting of Evaluations With Nonrandomized Designs (TREND) reporting guideline.

### Procedures

Patients were assessed at 13 sites in Spain from May 2019 through January 2021. The total trial duration was 4.5 years, including 1.5 years of recruitment, treatment, and follow-up (until February 28, 2022). Participants were given atezolizumab, 1200 mg, plus bevacizumab, 15 mg/kg, on day 1 of each 21-day (±3 days) cycle by intravenous infusion. Day 1 of cycle 1 treatment started within 1 to 5 days from enrollment. Treatment was continued until documented disease progression, unacceptable toxic effects, patient withdrawal, investigator decision, or death. If toxic effects were clearly attributed to 1 agent, that drug alone could be discontinued as long as the patient did not present with disease progression. Patients were allowed to continue receiving atezolizumab after apparent radiographic progression provided the benefit-to-risk ratio was judged to be favorable.

Tumor assessments by computed tomography imaging were done during screening (within 28 ± 12 days before enrollment) and every 12 weeks (±7 days) from day 1, cycle 1, until disease progression or loss of clinical benefit as applicable for patients who continued atezolizumab treatment beyond initial disease progression. The planned schedule of computed tomography scans was maintained even if a delay in treatment administration occurred. Response was assessed according to RECIST v1.1.

Laboratory tests assessing hematologic characteristics, blood chemistry parameters, and thyroid function and urinalysis were done within 14 days before enrollment and within 3 days prior to day 1 administration of each cycle. Adverse events (AEs) and abnormal laboratory findings were graded according to the National Cancer Institute Common Terminology Criteria for Adverse Events, version 4.0.^28^ Investigators assessed whether AEs were treatment related according to the study protocol and standard regulatory requirements. Molecular methods, including TMB, PD-L1, blood cell counts, biochemistry, ctDNA, and flow cytometry analyses, are described in the eResults in Supplement 2.

### End Points

The primary end point was investigator-assessed, 12-month PFS by RECIST v1.1 criteria. Progression-free survival was defined as the time from enrollment to the first occurrence of disease progression or death from any cause, whichever occurred first. Secondary end points included investigator-assessed overall response rate (ORR), duration of response (DOR), and time to response according to RECIST v1.1; 1-year overall survival (OS) rate; ORR and PFS according to PD-L1 expression; and safety and tolerability of atezolizumab plus bevacizumab combination therapy.

Prespecified exploratory end points included evaluation of the clinical utility of the TMB reports describing druggable alterations or driver sequence variations that may influence treatment selection (*KRAS*, *EGFR*, *BRAF*, *HER2*, *MET*, *ALK*, *RET*, and *ROS1*) in patients with TMB less than 10 mut/Mb; OS and ORR according to the TMB determination in blood and tumor samples; and peripheral blood immune cells and plasma levels of soluble factors and their changes during treatment as well as their correlation with clinical variables associated with treatment efficacy (PFS, OS, ORR, and DOR) and AEs. Additional end points are described in the eMethods in Supplement 2.

### Statistical Analysis

Progression-free survival, OS, and ORR were assessed in the per-protocol population, which included all patients who received at least 2 cycles of atezolizumab plus bevacizumab combination therapy or had at least the first tumor response evaluation carried out. The sample size was based on the number of events needed to demonstrate efficacy for the primary end point. For 1 arm, as an alternative hypothesis, we estimated achievement of a 12-month PFS rate of 40% (vs 18% as a null hypothesis achieved in previous studies with chemotherapy), with a 90% power at an α of 5% (1-sided test). The test statistic for survival probability was based on the nonparametric estimate of the survival distribution. Thus, with an estimation of 10% of errors, withdrawals, or other causes reducing the number of eligible patients, it was considered necessary to recruit 40 patients.

We used the Kaplan-Meier method to estimate PFS, OS, DOR, and corresponding 95% CIs. The reverse Kaplan-Meier method was used to calculate the median follow-up time and corresponding IQR. Categorical variables were presented as absolute and relative frequencies and numerical variables as mean (SD) or median (IQR). Spearman rank correlation coefficient was used for bivariate analysis. Comparisons between groups were done using nonparametric tests (Mann-Whitney U test or Wilcoxon signed rank test for 2 groups and Kruskal-Wallis test with Bonferroni correction for 3 or more groups). Cox proportional hazards regression models were used to assess the association of study variables with survival outcomes. *P* ≤ .05 was considered statistically significant, and all statistical tests were 2-sided. Statistical analyses were performed using GraphPad Prism software, version 8.0 (Dotmatics).

## Results

### Patient Characteristics

From May 2019 through January 2021, a total of 307 patients were assessed for eligibility at the 13 sites. Of these patients, 266 were ineligible for enrollment (149 with a TMB <10 mut/Mb, 41 with a TMB ≥10 mut/Mb but with other noneligibility reasons, 13 with a TMB that could not be determined, 24 with no tumor or an invalid sample, 21 with an insufficient sample, and 18 with other reasons).

Of the 41 patients enrolled (intention-to-treat population), 3 did not fulfill all inclusion criteria and were excluded (eResults in Supplement 2). The remaining 38 patients constituted the per-protocol population (12.3% of total screened patients) (eFigure 1 in Supplement 2). Overall, 10 patients (26.3%) were female, 28 (73.7%) were male, 36 (94.7%) were current or former smokers (median pack-years, 45; IQR, 30-74), 16 (42.1%) had a baseline ECOG performance status of 0, and 22 (57.9%) had a baseline ECOG performance status of 1. The mean (SD) age was 63.7 (8.3) years. The most frequent histological type was adenocarcinoma (35 patients [92.1%]), and 32 patients (84.2%) had stage IV disease (14 patients [36.8%] had stage IVA, and 18 patients [47.4%] had stage IVB) (Table 1). The most common comorbidities were hypertension (19 patients [50.0%]), dyslipemia (17 [44.7%]), chronic obstructive pulmonary disease (12 [31.6%]), and diabetes (11 [28.9%]) (eTable 1 in Supplement 2). As of February 28, 2022 (data cutoff), the median duration of follow-up was 22.1 months (IQR, 15.4-24.5 months).

**Table 1.** Baseline Patient Demographic and Clinical Characteristics

Characteristic	Patients (N = 38)^a^
Age, mean (SD), y	63.7 (8.3)
Sex	
Female	10 (26.3)
Male	28 (73.7)
BMI, mean (SD)	25.4 (4.1)
Smoking history	
Former (≥1 y)	21 (55.3)
Never (≤100 cigarettes per lifetime)	1 (2.6)
Smoker	15 (39.5)
Unknown	1 (2.6)
Pack-years, median (IQR)	45 (30-74)
White race^b^	38 (100)
ECOG performance status	
0	16 (42.1)
1	22 (57.9)
Histologic characteristics	
Adenocarcinoma	35 (92.1)
Large cell carcinoma	1 (2.6)
NOS or undifferentiated	2 (5.3)
Cancer stage	
IIIA	1 (2.6)
IIIB	3 (7.9)
IVA	14 (36.8)
IVB	18 (47.4)
Other	2 (5.3)

Abbreviations: BMI, body mass index (calculated as weight in kilograms divided by height in meters squared); ECOG, Eastern Cooperative Oncology Group; NOS, not otherwise specified.

^a^ Per-protocol population. Data are presented as number (percentage) of patients unless otherwise indicated.

^b^ Race was ascertained by self-report and was included in the analysis to control for possible associations with treatment outcomes or toxic effects.

### Primary End Point

**Figure 1.**
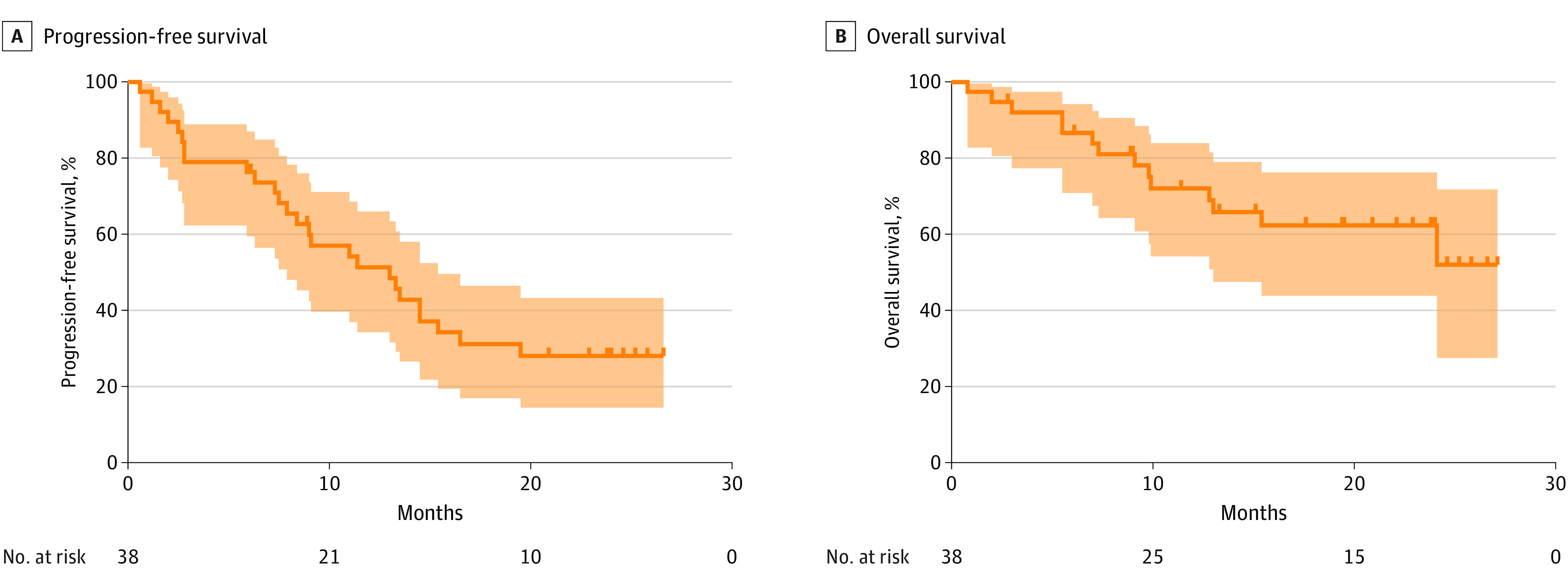
Kaplan-Meier Estimates of Progression-Free Survival and Overall Survival in the Per-Protocol Population Solid lines indicate survival estimates; shading, 95% CIs; and markers, censored cases.

As of data cutoff, 26 of 38 patients in the per-protocol population (68.4%) had experienced disease progression or had died: 12 patients (31.6%) had disease progression and were alive, and 14 patients (36.8%) had disease progression and died. The 12-month PFS rate was 51.3% (95% CI, 34.2%-66.0%; 96% data maturity), which met the study primary objective. The corresponding rate at 18 months was 31.1% (95% CI, 16.9%-46.4%; 92% data maturity), and the median duration of PFS was 13.0 months (95% CI, 7.9-18.0 months) (Figure 1A).

### Secondary End Points

The OS rate was 86.6% (95% CI, 70.8%-94.2%) at 6 months, 72.0% (95% CI, 54.1%-83.9%) at 12 months, and 62.3% (95% CI, 43.8%-76.2%) at 18 months (Figure 1B). Median OS was not reached at the time of analysis.

**Figure 2.**
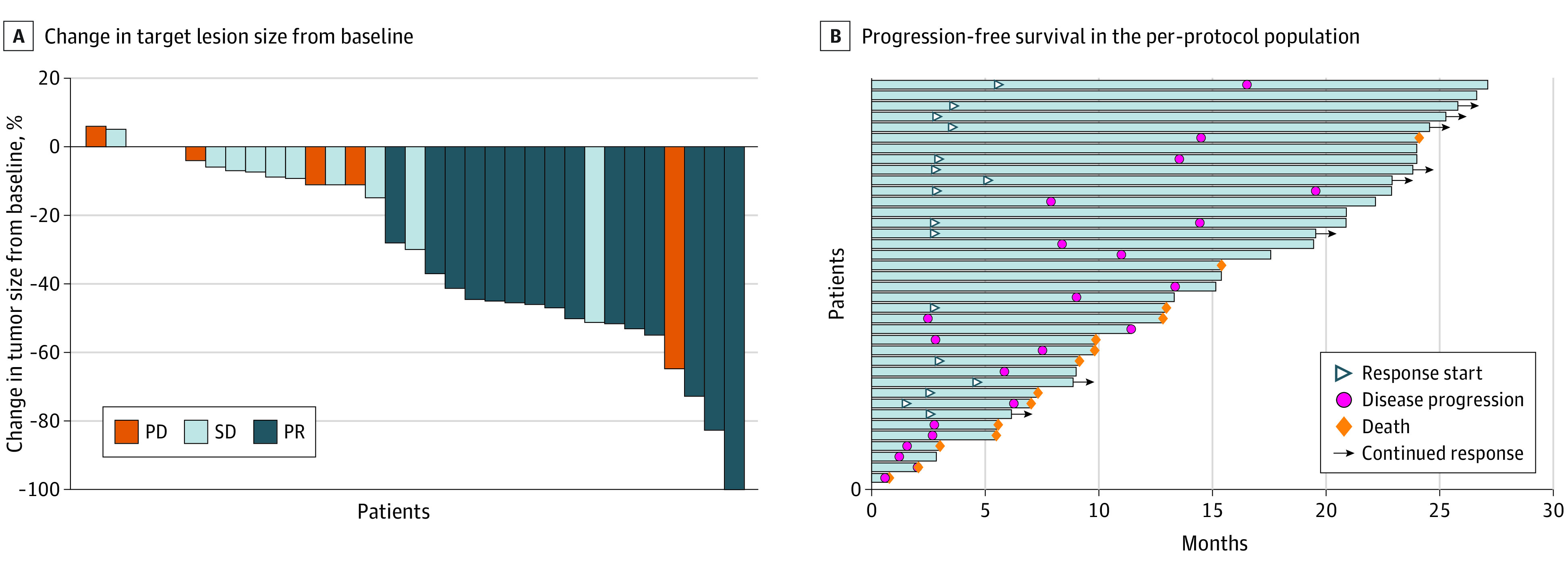
Tumor Response per Response Evaluation Criteria in Solid Tumours, Version 1.1 A, Waterfall plot of best percentage change in target lesion size from baseline. Bars along the x-axis represent individual patient data. B, Swimmer plot of progression-free survival in the per-protocol population (N = 38). Each bar represents 1 patient. At the time of data cutoff, 24 of 38 patients (63.2%) were alive, of whom 12 (31.6%) were free of recurrence. Twenty-six patients (68.4%) had experienced disease progression or had died: 14 patients (36.8%) had disease progression and died, and 12 (31.6%) had disease progression. PD indicates progressive disease; PR, partial response; SD, stable disease.

According to RECIST v1.1 criteria, 16 of 38 patients in the per-protocol population (42.1%) achieved an objective response (0 complete responses and 16 partial responses) and 30 (78.9%) achieved disease control (Table 2 and Figure 2A). The median time to response was 2.8 months (IQR, 2.8-3.58 months), with a median DOR of 11.7 months (range, 3.57-22.4 months; the response was ongoing at cutoff). Responses were durable, with 8 of 16 responses (50.0%) ongoing at cutoff. Of the 8 patients who had a partial response but subsequently had disease progression, 4 (50.0%) were alive at cutoff (Figure 2B).

**Table 2.** Investigator-Assessed Tumor Response and Duration of Response

Response	Patients (N = 38)^a^
Objective response^b^	16 (42.1)
Best overall response	
Complete response	0
Partial response	16 (42.1)
Stable disease	14 (36.8)
Progressive disease	7 (18.4)
Missing data	1 (2.6)
Time to response, median (IQR), mo	2.8 (2.8-3.58)
Duration of response, median (range), mo^c^	11.7 (3.57-22.4)

^a^ Per-protocol population. Data are presented as number (percentage) of patients unless otherwise indicated.

^b^ Defined as a confirmed complete response or partial response as ascertained by the investigator according to Response Evaluation Criteria in Solid Tumours, version 1.1. Only patients with measurable disease at baseline were included in the analysis of patients achieving an objective response.

^c^ Responses were ongoing at cutoff.

### Safety

All-grade AEs associated with atezolizumab treatment occurred in 29 of 38 patients in the per-protocol population (76.3%). The most common grade 1 or 2 AEs associated with atezolizumab were fatigue (6 of 38 patients [15.8%]), pruritus (6 [15.8%]), anorexia (5 [13.2%]), and diarrhea (4 [10.5%]). Grade 3 or 4 AEs associated with atezolizumab treatment were reported in 5 patients (13.2%), including increased alanine aminotransferase level (1 of 38 patients [2.6%]), arthralgia (1 [2.6%]), arthritis (1 [2.6%]), diarrhea (1 [2.6%]), and increased serum amylase level (1 [2.6%]). All-grade AEs associated with bevacizumab treatment occurred in 23 of 38 patients in the per-protocol population (60.5%). The most common grade 1 or 2 AEs associated with bevacizumab were hypertension (10 of 38 patients [26.3%]), proteinuria (4 [10.5%]), anorexia (3 [7.9%]), and diarrhea (3 [7.9%]). Grade 3 or 4 AEs associated with bevacizumab treatment were reported in 6 patients (15.8%) and included hypertension (2 of 38 patients [5.3%]), increased alanine aminotransferase level (1 [2.6%]), anal fistula (1 [2.6%]), myocardial infarction (1 [2.6%]), and vascular disorders (1 [2.6%]). No treatment-related AEs leading to death occurred (Table 3).

**Table 3.** Treatment-Related Adverse Events in All Recipients of Atezolizumab Plus Bevacizumab

Adverse event^a^	Patients, No. (%) (N = 38)	
	Grade 1 or 2 adverse event	Grade 3 or 4 adverse event
**Atezolizumab**		
Alanine aminotransferase level increased	0	1 (2.6)
Arthritis	0	1 (2.6)
Fatigue	6 (15.8)	0
Pruritus	6 (15.8)	0
Anorexia	5 (13.2)	0
Diarrhea	4 (10.5)	1 (2.6)
Vomiting	3 (7.9)	0
Arthralgia	2 (5.3)	1 (2.6)
Hypothyroidism	2 (5.3)	0
Mucositis oral	2 (5.3)	0
Nausea	2 (5.3)	0
Proteinuria	2 (5.3)	0
Rash acneiform	2 (5.3)	0
Serum amylase level increased	2 (5.3)	1 (2.6)
Skin and subcutaneous tissue disorder	2 (5.3)	0
Aphonia	1 (2.6)	0
Back pain	1 (2.6)	0
Creatinine concentration increased	1 (2.6)	0
Dizziness	1 (2.6)	0
Dry mouth	1 (2.6)	0
Dry skin	1 (2.6)	0
Dysgeusia	1 (2.6)	0
Edema limbs	1 (2.6)	0
Flatulence	1 (2.6)	0
Gastrointestinal disorders	1 (2.6)	0
General disorders and administration	1 (2.6)	0
Hepatobiliary disorders	1 (2.6)	0
Hypomagnesemia	1 (2.6)	0
Lipase increased	1 (2.6)	0
**Bevacizumab**		
Hypertension	10 (26.3)	2 (5.3)
Alanine aminotransferase level increased	0	1 (2.6)
Anal fistula	0	1 (2.6)
Myocardial infarction	0	1 (2.6)
Vascular disorders	0	1 (2.6)
Proteinuria	4 (10.5)	0
Anorexia	3 (7.9)	0
Diarrhea	3 (7.9)	0
Fatigue	2 (5.3)	0
Gastrointestinal disorders	2 (5.3)	0
Mucositis oral	2 (5.3)	0
Aphonia	1 (2.6)	0
Arthralgia	1 (2.6)	0
Back pain	1 (2.6)	0
Dysgeusia	1 (2.6)	0
Flatulence	1 (2.6)	0
Gingival pain	1 (2.6)	0
Hypomagnesemia	1 (2.6)	0
Nausea	1 (2.6)	0
Neck pain	1 (2.6)	0
Oral hemorrhage	1 (2.6)	0
Periodontal disease	1 (2.6)	0
Pruritus	1 (2.6)	0
Respiratory, thoracic, and mediastinal disease	1 (2.6)	0
Serum amylase level increased	1 (2.6)	0
Vomiting	1 (2.6)	0

^a^ All adverse events that occurred during the trial period or within 30 days from the last dose administration.

Adverse events leading to discontinuation of atezolizumab occurred in 2 of 38 patients (5.3%; both grade 3 AEs), AEs leading to a delay in atezolizumab administration occurred in 9 of 38 patients (23.7%; 1 grade 1, 4 grade 2, and 4 grade 3 AEs), and AEs leading to atezolizumab dose omission occurred in 3 of 38 patients (7.9%; all grade 3 AEs). Adverse events leading to discontinuation of bevacizumab occurred in 3 of 38 patients (7.9%; 1 grade 2 and 2 grade 3 AEs), AEs leading to a delay in bevacizumab administration occurred in 9 of 38 patients (23.7%; 2 grade 1, 3 grade 2, and 4 grade 3 AEs), and AEs leading to bevacizumab dose omission occurred in 10 of 38 patients (26.3%; 2 grade 1, 2 grade 2, and 6 grade 3 AEs) (eTable 2 in Supplement 2).

### Biomarkers

The PD-L1 tumor proportion score was available in 30 patients (78.9%). No association between PD-L1 tumor proportion score and ORR, PFS, or OS was observed. Tumor mutation burden determined from tissue samples was available for all patients (n = 38), and TMB was higher in patients with an objective response, with a median TMB of 15.5 mut/Mb (IQR, 11.5-24.5 mut/Mb) compared with 13 mut/Mb (IQR, 10.5-15.0 mut/Mb) in patients with progressive disease or stable disease (*P* = .03). However, no differences were observed in PFS or OS (eFigures 2-4 in Supplement 2).

The percentage of screened tumors with druggable alterations was lower in the subgroup with TMB of 10 mut/MB or more (14 of 82 patients [17.1%]) compared with the subgroup with TMB less than 10 mut/Mb (56 of 149 patients [37.6%]) (*P* = .001) (eFigure 5 in Supplement 2). Regarding the per-protocol population, sequence alterations in *KRAS* or *P53* genes had no association with ORR, PFS, or OS (eFigure 6 in Supplement 2). However, the presence at diagnosis of at least 1 sequence variation in *KEAP*, *RB1*, *VEGFA*, *PTEN*, or *HER2* (eFigure 7 in Supplement 2); elevated baseline lactate dehydrogenase or alkaline phosphatase plasma levels (eFigure 8 in Supplement 2); and higher percentage of PD-1–positive peripheral blood T cells during treatment (eFigure 9 in Supplement 2) was associated with worse prognosis. Flow cytometry analysis of paired response and progression samples is shown in eFigure 10 in Supplement 2. None of the patients who showed a ctDNA decrease during treatment (n = 4) had died (eFigure 11 in Supplement 2). Finally, the association of clinical and molecular variables with PFS and OS were assessed using Cox proportional hazards regression (eFigure 12 in Supplement 2).

## Discussion

Strategies to overcome treatment resistance and increase the proportion of patients who benefit from immunotherapy include the combination of PD-1 and PD-L1 inhibitors with conventional cytotoxic chemotherapy and/or targeted therapies as well as the identification of predictive biomarkers of response.^29,30,31,32,33^ Thus, dual immune modulation with PD-1 and PD-L1 and VEGF inhibitors has shown synergistic activity, providing clinical benefits over each therapy alone in different tumor types, including NSCLC.^4,8,34,35,36,37,38,39^ Likewise, TMB has emerged as a predictive biomarker for checkpoint inhibitor–based immunotherapy in several cancer types, including NSCLC.^40,41,42,43,44^

To our knowledge, TELMA is the first prospective study to evaluate TMB as a biomarker to estimate survival benefit associated with the combination of atezolizumab plus bevacizumab in treatment-naive patients with locally advanced or metastatic nonsquamous NSCLC with no *EGFR* or *ALK* genomic alterations. In patients with a high TMB, the addition of bevacizumab to first-line atezolizumab was associated with an encouraging and durable survival benefit, with 51.3% of patients having progression-free disease and 72.0% of patients being alive at 1 year. The median PFS was 13.0 months, while the median OS was not reached at the time of analysis. The investigator-assessed ORR was 42.1%, and the median DOR was 11.7 months, with 50.0% of those with a response having ongoing responses at the time of the last follow-up. The combination of atezolizumab plus bevacizumab was well tolerated. Most treatment-related AEs were grade 1 or 2 and were consistent with the known safety profile of each agent and the underlying disease. New safety signals were not identified.

Although cross-trial comparisons are limited by study design and patient populations, in general, the survival benefit observed in the TELMA study is encouraging considering that of previously reported phase 3 trials, including the IMpower110 trial of atezolizumab monotherapy (12-month PFS and OS rate in patients with high PD-L1 level of 36.9% and 64.9%, respectively),^7,10^ the IMpower130 trial of atezolizumab plus carboplatin plus nab-paclitaxel (12-month PFS and OS rate regardless of PD-L1 expression of 29.1% and 63.1%, respectively),^6^ the IMpower132 trial of atezolizumab plus carboplatin or cisplatin plus pemetrexed (12-month PFS rate in patients with high PD-L1 level of 46%),^45^ and the IMpower150 trial of atezolizumab plus bevacizumab, carboplatin, and paclitaxel (median PFS of 12.6 months in patients with high PD-L1 level).^4,8^ In addition, the survival benefits associated with atezolizumab plus bevacizumab in patients with a PD-L1 tumor proportion score of 50% or more from the phase 2 @Be study^46^ were comparable to those in the TELMA study. The median PFS was 15.9 months (12-month PFS rate, 54.9%), and the median DOR was 10.4 months; the median OS was not reached at the time of analysis. The ORR in the @Be study (64.1%) was higher than the ORR in the TELMA study (42.1%).

Of note, the population in the TELMA study had somewhat worse basal characteristics than the population in the @Be study^46^ (ie, higher proportion of patients with an ECOG performance status of 1 [57.9% vs 35.9%] and higher proportion of patients with stage IVB disease [47.4% vs 38.5%]), which may have negatively impacted the outcomes. In this sense, biomarkers of tissue damage, such as elevated plasma levels of lactate dehydrogenase or alkaline phosphatase, were associated with worse PFS and OS in our study.^47^

PD-L1 and TMB are independent biomarkers of response to immunotherapy in most cancer types,^48^ and the combination of both may be better at predicting outcomes than any single biomarker.^41^ In our study, there was no correlation between TMB and PD-L1 levels, similar to previous results in unselected populations for TMB. Of note, it has been shown that the overlap between blood-based TMB and PD-L1 positivity ranges between 10% and 15% of cases.^7,41^ These data suggest that the patients who benefited from the atezolizumab plus bevacizumab combination in the @Be study^46^ and those in the TELMA study were 2 different but similarly sensitive subpopulations. In addition, our results seem to indicate that PD-L1 levels have no added value in estimating response or survival in the population with high TMB.

### Limitations

Our study has limitations, including the single-arm study design, the limited patient cohort size, the incomplete follow-up period for long-term survival analysis, and the reduced number of blood samples available for exploratory studies. Even so, the 12-month survival rates reported in both the TELMA study (72.0%) and the @Be study (70.6%)^46^ are higher than or noninferior to the best-reported rates with atezolizumab.^4,6,7,8,10,45^ In addition, our results are in line with those of previous studies^4,8,46^ showing that the incidence of treatment-related AEs of grade 3 or higher is less frequent with the combination of atezolizumab plus bevacizumab than with chemotherapy-containing regimens, resulting in a lower treatment discontinuation rate owing to toxic effects.

## Conclusions

In this nonrandomized controlled trial, we found that the combination of atezolizumab plus bevacizumab as first-line treatment for patients with advanced nonsquamous NSCLC with high TMB and no *EGFR* or *ALK* genomic alterations was associated with encouraging survival rates and durable responses, with a favorable safety profile. The superiority—or noninferiority—of the combination compared with PD-1 and PD-L1 inhibitor monotherapy or in combination with chemotherapy in patients with high TMB warrants further study, and this combination may become a standard treatment in this population.

## Notes

Supplement 1.
Trial Protocol

Supplement 2.
eMethods.

eResults.
**eFigure 1.** Disposition of Patients in the Study **eFigure 2.** PD-L1 and TMB Biomarkers **eFigure 3.** PD-L1 Categories and ORR **eFigure 4.** TMB and PD-L1 Correlation **eFigure 5.** TMB and Druggable Alterations in All Screened Patients **eFigure 6.** High Prevalence Sequence Variations (*KRAS* or *P53*) and Outcomes (ORR and PFS-OS) **eFigure 7.** Sequence Variation Signature and Outcomes (ORR and PFS-OS) **eFigure 8.** Blood Counts and Biochemistry With Outcomes (ORR and PFS-OS) **eFigure 9.** Flow Cytometry Analysis of Samples During Treatment and PFS **eFigure 10.** Flow Cytometry Analysis of Paired Response and Progression Samples **eFigure 11.** ctDNA Follow-up **eFigure 12.** Impact of Clinical and Molecular Variables on PFS and OS **eTable 1.** Associated Comorbidities (Per-Protocol Population) **eTable 2.** Treatment Compliances

Supplement 3.
Data Sharing Statement
