# Supplementary material for: Atezolizumab Plus Bevacizumab as First-line Treatment for Patients With Metastatic Nonsquamous Non–Small Cell Lung Cancer With High Tumor Mutation Burden: A Nonrandomized Controlled Trial
Source: JAMA Oncol. 2022 Dec 15;9(3):344–53. doi: 10.1001/jamaoncol.2022.5959 (PMC9856905; doi:10.1001/jamaoncol.2022.5959)
Supplement: Supplement 1. — Trial Protocol [file jamaoncol-e225959-s001.pdf]

Av. Meridiana, 358 / 6ªplanta
08027 Barcelona
Tel. 934 302 006
Fax. 934 191 768
Email: gecp@gecp.org
www.gecp.org

A PHASE II OPEN LABEL STUDY OF ATEZOLIZUMAB IN COMBINATION WITH BEVACIZUMAB AS FIRST LINE TREATMENT FOR LOCALLY ADVANCED OR METASTASIC HIGH-INTERMEDIATE TUMOR MUTATION BURDEN (TMB) SELECTED NON-SQUAMOUS NON-SMALL CELL LUNG CANCER (NSCLC) PATIENTS

TELMA: Atezolizumab plus bevacizumab in first line NSCLC patients

Study Sponsor: Fundación GECP

EudraCT Number: 2018-004654-17
Sponsor code: GECP 18/03
Roche code: ML40237

Version 5.0

Contacts

**Trial Chair**	Dr. Mariano Provencio, MD, PhD<br><br>Fundación GECP President	Oncology Service<br><br>Hospital Univ. Puerta de Hierro, Majadahonda, Madrid,<br><br>Tel. 93 430 20 06<br>Fax. 993 419 17 68<br><br>Email: secretaria@gecp.org
**Safety and Regulatory Affairs**	Coordinating office of the Fundación GECP	Beatriz García<br><br>Avenida Meridiana 358, 6ª planta<br>08027 Barcelona<br>Tel. +34 93 430 20 06<br>Fax. +34 93 419 17 68<br>Email: pharmacovigilance.slcg@gecp.org
**Coordinating office of the Fundación GECP**<br><br>**Monitoring team**	Monitor/CRA	Montse Cairó<br>Avenida Meridiana 358, 6ª planta<br>08027 Barcelona<br>Tel. +34 93 430 20 06<br>Fax. +34 93 419 17 68<br>Email: mcairo@gecp.org
	Lead CRA	Ana Verdú<br>Avenida Meridiana 358, 6ª planta<br>08027 Barcelona<br>Tel. +34 93 430 20 06<br>Fax. +34 93 419 17 68<br>Email: averdu@gecp.org
	Clinical Operations Manager:	Eva Pereira<br>Avenida Meridiana 358, 6ª planta<br>08027 Barcelona<br>Tel. +34 93 430 20 06<br>Fax. +34 93 419 17 68<br>Email: epereira@gecp.org

Protocol Signature Page

A PHASE II OPEN LABEL STUDY OF ATEZOLIZUMAB IN COMBINATION WITH BEVACIZUMAB AS FIRST LINE TREATMENT FOR LOCALLY ADVANCED OR METASTATIC HIGH-INTERMEDIATE TUMOR MUTATION BURDEN (TMB) SELECTED NON-SQUAMOUS NON-SMALL CELL LUNG CANCER (NSCLC) PATIENTS

TELMA: Atezolizumab plus bevacizumab in first line NSCLC patients

Sponsor code: GECP 18/03

Approved by:

Signature

____

Dr. Mariano Provencio
Trial Chair

Signature

____

Dr. Bartomeu Massutí
Fundación GECP Secretary

Signature

____

Dr. Mariano Provencio
Fundación GECP President

Principal Investigator Protocol Signature Page

Study Title: “A phase II open-label study of Atezolizumab in combination with bevacizumab as first line treatment for locally advanced or metastatic high-intermediate tumor mutation burden (TMB) selected non-squamous non-small cell lung cancer (NSCLC) patients” **TELMA**

Sponsor protocol code: GECP 18/03
EudraCT Number: 2018-004654-17
Protocol version: v 5.0,20th January 2022

As principal investigator of this site, I hereby confirm that:

I have read the protocol and agree that it contains all necessary details for conducting this trial. I will conduct the trial as outlined in the following protocol and in compliance with GCP and will apply due diligence to avoid protocol deviations.

I will provide copies of the protocol and all drug information relating to pre-clinical and prior clinical experience furnished to me by the Fundación GECP, to all physicians responsible to me who participate in this trial. I will discuss this material with them to assure that they are fully informed regarding the drug and the conduct of the trial.

I agree to keep accurate records on all patient information including patient's informed consent statement, drug shipment and return forms, and all other information collected during the trial for a minimum period of 25 years according to the new Royal Decree 1090/2015 approved in Spain.

Name of Principal Investigator: ____________________________________

Institution’s name and place: ______________________________________

Signature   Date

TABLE OF CONTENTS

Section **Page**

1. Protocol summary ... 7
2. Trial schedule ... 20
3. Background and rationale ... 23
   3.1. Disease background and treatments in advanced NSCLC patients ... 23
   3.2. Immunotherapy ... 26
   3.3. Background on Atezolizumab ... 26
   3.4. Background on Bevacizumab ... 30
   3.5. Rationale for trial design ... 31
   3.6. Rationale for additional translational research (stool and blood samples) ... 32
4. Objectives and endpoints ... 33
   4.1. Primary objective and endpoint ... 33
   4.2. Secondary objectives and endpoint ... 34
   4.3. Exploratory objectives ... 35
5. Trial design, duration and termination ... 35
6. Patient selection ... 37
   6.1. Inclusion criteria ... 37
   6.2. Exclusion criteria ... 39
7. Patient pre-screening and enrollment ... 43
8. Trial procedures ... 44
   8.1. Overview of treatment sequence ... 44
9. Investigational Medicinal Product ... 52
   9.1. Packaging and labelling ... 53
   9.2. Receipt and storage of the IMP ... 53
   9.3. Unused trial drug supplies ... 53
10. Trial treatments description ... 54
   10.1. ATEZOLIZUMAB ... 54
   10.2. BEVACIZUMAB ... 55
11. Dose modifications criteria ... 55
   11.1. General Notes Regarding Dose Modification ... 55
   11.2. ATEZOLIZUMAB dose delay criteria ... 55
   11.3. BEVACIZUMAB dose delay/reduction criteria ... 56
12. Prohibited and permitted concomitant treatments ... 61
13. Adverse events and reporting ... 63
   13.1. Definition of an Adverse Event (AE) and AE's reporting ... 63
   13.2. Definition of Serious Adverse Event (SAE) and Suspected Unexpected Serious Adverse Reaction (SUSAR) ... 66
   13.3. Adverse events of Special Interest for Atezolizumab (Immediately reportable to the Sponsor) ... 68
   13.4. Overdose and secondary malignancies ... 69
   13.5. SAEs and SUSARs reporting ... 69
   13.6. Pregnancy ... 70
   13.1. Other safety considerations ... 71
   13.2. Adverse events related to the treatment and associated risks ... 71
14. Biological material and translational research ... 73

15. Case report forms and documentation 75
16. Statistical considerations 75
17. Criteria for termination of the trial 76
17.1. General criteria for termination of the trial 76
17.2. Discontinuation of protocol treatment and from the study for individual patients 76
18. Ethics aspects, regulatory approval, and Patient Informed Consent 77
18.1. Ethical Review Board/Ethics Committee/Health Authority 77
18.2. Informed consent 78
19. Governance and administrative issues 79
19.1. Study documentation 79
19.2. Protocol non-compliances/deviations 79
19.3. Protocol amendment 79
19.4. Final report 79
19.5. Independent Data Monitoring Committee 79
19.6. Publication 79
19.7. Financial disclosure 80
19.8. Clinical trial insurance 80
19.9. Quality assurance 80
19.10. Data protection 80
19.11. Study monitoring team 81
19.12. Record retention 81
20. References 82
20.1. References for translational research 86

Appendices:

1. Common Terminology criteria for Adverse Events (CTCAE)
2. RECIST criteria v1.1
3. SAE Form
4. Pregnancy Form
5. Health-related Quality of life questionnaire: EORTC C30 (QLQ-C30) and submodule LC13
6. System of classification for non-microcytic lung cancer (NSCLC) and definition of lymph node maps, 8th edition
7. Risks associated with Atezolizumab and guidelines for management of adverse events associated with Atezolizumab
8. Patient pre-screening form TELMA
9. Foundation one CDx Specimen Instructions
10. Foundation one Liquid technical sheet
11. Foundation one CDx technical sheet

Tables:

Table 1. Administration of First and Subsequent Infusions of Atezolizumab

1. Protocol summary

A PHASE II OPEN LABEL STUDY OF ATEZOLIZUMAB IN COMBINATION WITH BEVACIZUMAB AS FIRST LINE TREATMENT FOR LOCALLY ADVANCED OR METASTATIC HIGH-INTERMEDIATE TUMOR MUTATION BURDEN (TMB) SELECTED NON-SQUAMOUS NON-SMALL CELL LUNG CANCER (NSCLC) PATIENTS

TELMA: Atezolizumab plus bevacizumab in first line NSCLC patients

Sponsor: Fundación GECP

Trial Chair: Mariano Provencio Pulla (MD, PhD)

Pharma Partner: Hoffmann-La Roche

Protocol code: GECP 18/03 (ML40237)

Population: stage IIIB - IV non-squamous selected high-intermediate tumor mutation burden (TMB) non-small cell lung cancer patients

Design: Open-label, non-randomized, phase II multi-centre clinical trial

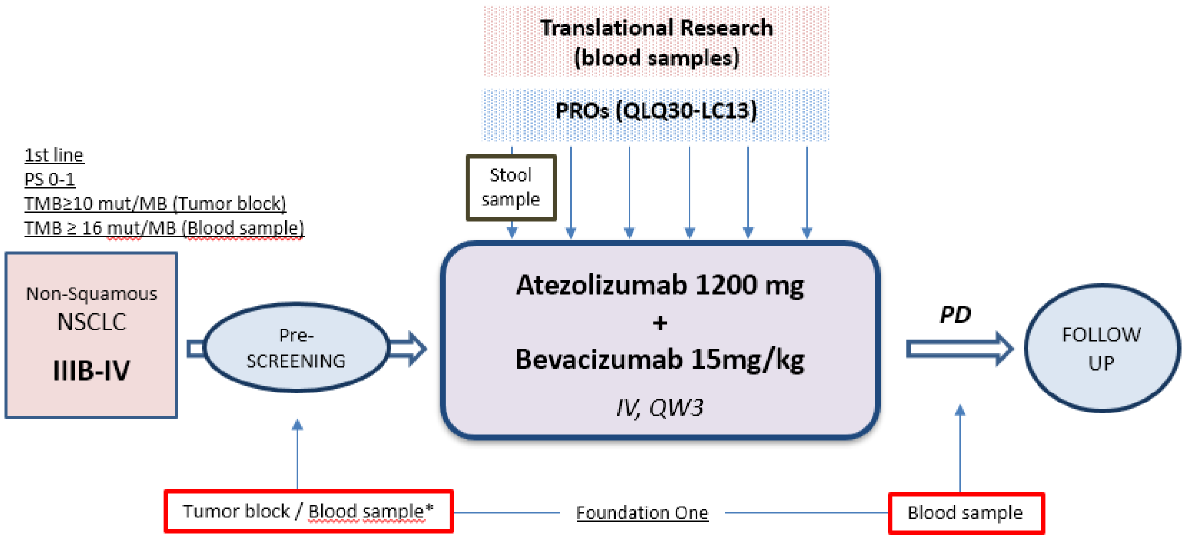

* If the submitted tumor sample is insufficient or not valid to evaluate the TMB, a blood sample can be sent instead .

Sample size: 40 patients

Rationale:

Despite recent improvements in treatment, the prognosis for patients with advanced NSCLC remains dismal, with a median OS of approximately 12.5 months[3].

Inhibition of programmed cell death protein-ligand 1/programmed cell death protein 1 (PD-L1/PD-1) signalling has been shown to induce durable responses in some patients with different tumors, and expression of PD-L1 by tumor cells (TCs) in several tumor types (including NSCLC) correlates with response to therapy[4]. Immunotherapy has demonstrated that therapies focused on enhancing T-cell responses against cancer may result in a significant survival benefit in patients with Stage IV cancer[5,6,7].

Immunotherapeutic targeting of PD-L1 or PD-1 is revolutionizing the treatment of metastatic NSCLC. Currently, two anti-PD-1 therapeutic antibodies (Pembrolizumab and Nivolumab) and one anti-PD-L1 (Atezolizumab) have been approved for NSCLC treatment. Both agents are recommended as second-line agents in advanced NSCLC after prior progression on platinum-containing chemotherapy.

Atezolizumab is a humanized anti-PD-L1 monoclonal antibody that inhibits its interaction with its receptors, PD-1 and B7.1 (CD80, B7-1), reinvigorating anticancer immunity. The OAK study evaluated the efficacy of Atezolizumab compared to Docetaxel in 1.225 patients previously treated with locally-advanced or metastatic NSCLC. The PD-L1 targeted therapy by Atezolizumab resulted in a clinically relevant improvement of overall survival *versus* Docetaxel regardless of PD-L1 expression or histology, with a favourable safety profile. The median OS in this study was 13.8 months (95% CI 11.8-15.7) in the Atezolizumab arm compared to 9.6 months (95% CI 8.6-11.2) in the Docetaxel arm (HR=0.73, 95% CI 0.62-0.87; p=0.0003)[8].

The GO28625 (NCT01846416, FIR) phase 2 study enrolled 137 PD-L1-selected NSCLC patients regardless previous treatments. The primary analysis for efficacy-evaluable patients, confirmed ORR after the first-line treatment with Atezolizumab was 28.6% (95% CI: 3.7-71.0) in IHC TC3 or IC3 patients and 25.8% (95% CI: 11.9-44.6) in IHC TC2/3 or IC2/3 patients[9].

The efficacy and safety of Atezolizumab was also evaluated in another phase 2 single-arm study where 142 PD-L1-selected advanced NSCLC patients (NCT02031458, BIRCH) were treated with Atezolizumab monotherapy. The primary endpoint ORR in the ITT population was 25%, in TC3 or IC3 subgroup was 34% and 18% in TC2 and IC2. Median PFS and OS for the entire population were 7.3 and 23.5 months respectively. These results indicate that Atezolizumab monotherapy has durable efficacy in the first line setting[10].

Currently, first-line treatment for advanced NSCLC without presence of genetic aberrations, such as sensitizing mutations of epidermal growth factor receptor (EGFR) or translocations of anaplastic lymphoma kinase (ALK), are chemotherapy and Pembrolizumab, approved based on KEYNOTE-024 study. This study randomized patients with advanced NSCLC and PD-L1 expression (at least 50% of

tumor cells), showing median progression-free survival (PFS) of 10.3 months (95% CI 6.7-not reached) versus 6.0 months (95% CI 4.2-6.2) (HR 0.50; 95% CI 0.37-0.68; P<0.001) for the Pembrolizumab and chemotherapy groups respectively[11].

Currently there is a clinical trial (NCT02409342, IMpower110) of Atezolizumab as first line monotherapy in PD-L1 selected NSCLC patients that is expected to add the first line approval to Roche´s portfolio. Thus, all the above data support the rational of cancer immunotherapy in first line PD-L1 positive NSCLC[12]. In addition, there is a growing body of evidence that suggests that pro-angiogenic factors can modulate the immune response and may serve as mechanisms of scape. Therefore, the combination of cancer immunotherapy with antiangiogenic agents makes sense.

The vascular endothelial growth factor (VEGF) is the most important pro-angiogenic protein and a key regulator of physiological angiogenesis. It is also implicated in pathological angiogenesis associated with tumor growth. Indeed, increased levels of VEGF have been found in lung cancer; moreover, the overexpression of VEGF is associated with a poor prognosis[13,14]. Actually, VEGF plays a role in cancer immune evasion through modification of the endothelial cells in the tumor microenvironment, where VEGF may reduce lymphocyte adhesion and migration from vessels to the tumor site contributing to decreased immune cell recruitment[15]. Given the immunosuppressive role of VEGF and angiogenesis within tumors, it is not surprising that there is evidence supporting how antiangiogenic agents stimulate the immune response and enhance the efficacy of immunotherapies[16].

Recent findings indicating an intertwined regulation of VEGF signalling and immunosuppression in the tumor microenvironment suggest that the combination of anti-VEGF agents and immune checkpoint blockade could have synergistic antitumor activity, along with favourable tolerability[16].

Bevacizumab is a recombinant, humanized therapeutic anti-VEGF antibody that inhibits tumor angiogenesis which may correct the immunosuppressive function exerted by VEGF, increasing the infiltration of T effector cells in cancer[17].

So far, the more advanced studies to address the combination of cancer immunotherapy (CIT)-antiangiogenic agents such as Bevacizumab, there is a phase 3 study of Paclitaxel, Carboplatin Bevacizumab and Atezolizumab (NCT02366143, IMpower150). IMpower150 is the first phase III immunotherapy-based combination study to demonstrate a statistically significant and clinically meaningful improvement in PFS in all-comer 1L NSQ mNSCLC, providing a potential new standard of care for patients, showing a median PFS of 8.3 moths vs 6.8 moths (HR 0.62; 95% CI: 0.52, 0.74; P < 0.0001) for the Atezolizumab, chemotherapy and Bevacizumab arm (ABCP) and Carboplatin, Paclitaxel and Bevacizumab arm (BCP) respectively. In terms of PFS 12 months the results were 37% in the Atezolizumab arm vs 18% un the CT + Bevacizumab arm. Median overall survival among the patients was longer in the ABCP group than in the BCP group (19.2 months vs. 14.7 months; hazard ratio for death, 0.78; 95% CI, 0.64 to 0.96; P = 0.02)[18].

The phase 3 study of Atezolizumab in combination with Bevacizumab compared to Sunitinib single agent (IMmotion151), as first-line therapy in untreated metastatic Renal Cell Carcinoma (RCC) patients. This clinical trial showed a significant benefit of 3.5 months in the PFS favouring the CIT-Bevacizumab combination (11.2 m vs. 7.7 m) with a tolerable safety profile in PD-L1+ patients[19].

These data confirm the role of Bevacizumab as an immune modulating agent that may provide a successful combination strategy in different tumor types.

Increased mutation rate is a well-characterized feature of human cancer. Abnormal activity in several cellular pathways, including DNA damage repair and DNA replication, can increase the overall rate of somatic mutations in tumors, as can exposure to mutagens such as ultraviolet light and tobacco smoke[20]. Defects in DNA damage repair lead to the accumulation of mutations caused by replicative errors and environmental damage[21].

The core DNA mismatch repair protein complex is composed of two cooperative dimers: the PMS2 protein dimerizes with MLH1 to form the complex MutL-alpha, which cooperates with the MSH2-MSH6 dimer, MutS-alpha, to repair single base pair mismatches and small insertion–deletion loops[22]. PMS2 promoter mutations are present in ~10% of melanoma samples and ~8% of squamous cell carcinomas[23].

Perturbations in mismatch repair gene expression, both loss and overexpression, can be deleterious to genomic stability, and loss of function mutations in mismatch repair pathway genes are known to correlate with high Tumor Mutational Burden (TMB) in tumors, overall mutations in these genes accounted for less than 10% of cases with high TMB[23].

As such, tumors with defective DNA repair mechanisms are more likely to benefit from immunotherapy. DNA replication is another key pathway in which defects can lead to increased somatic mutation rate.

Loss of function mutations in TP53 are very common in cancer and are a somatic marker of elevated mutation rate[24]. Mutations in a number of other genes have also been linked to increase TMB. KRAS-mutant lung cancers account for approximately 25% of non-small cell lung carcinomas, thus representing an enormous burden of cancer worldwide. KRAS mutations are clear drivers of tumor growth and are characterized by a complex biology involving the interaction between mutant KRAS, various growth factor pathways, and tumor suppressor genes. KRAS mutations are classically associated with a significant smoking history[25]. Then, it is an emerging biomarker for response to immunotherapy. The CheckMate 026 trial is a randomized trial comparing the treatment efficacy of Nivolumab versus chemotherapy in first line therapy of advanced non-small cell lung cancer based on the programmed death-ligand 1 expression. However, there was no strong correlation between treatment response and PD-L1 expression. Therefore, researchers performed a subanalysis to assess if the TMB could determine Nivolumab treatment response in 312 patients with evaluable TMB data. The results demonstrated that patients with a high mutation burden had a better progression free survival (9.7 vs 5.8 months; HR, 0.62) and objective response rate (46.8% vs 28.3%) compared with the control arm. These findings imply that tumor mutation burden may be considered as a biomarker for immunotherapy26.

Regarding TMB data, the CheckMate227 study is an open-label, phase 3 trial, that examined progression-free survival with nivolumab plus ipilimumab versus chemotherapy among patients with stage IV or recurrent NSCLC that was not previously treated with chemotherapy with a high tumor mutational burden (≥10 mutations per megabase). Median PFS among patients with a high tumor mutational burden was significantly longer with nivolumab plus ipilimumab than with chemotherapy, 7.2 months (95% confidence interval [CI], 5.5 to 13.2) versus 5.5 months (95% CI, 4.4 to 5.8) (HR, 0.58; 97.5% CI, 0.41 to 0.81; P<0.001)[27].

Further understanding the factors associated with increased TMB is important for better understanding this key driver of cancer progression and for understanding the molecular mechanisms which lead to TMB.

Unlike the majority of genetic panels used within the standard of practice in oncology, even those using Next Generation Sequencing (NGS), Foundation Medicine ® (FMI) is a "Comprehensive Genomic Profiling" test, able to detect all cancer-relevant genes with all classes of alterations across the entire coding region, being also a validated performance[36]. Moreover, is a hybridization capture-based NGS method which, contrary to the more common Hot Spot Multi-gene panels, do not miss any relevant gene or genetic alteration[37]. Besides, FMI has two formats, both of them validated in top tier peer-reviewed journal: "FoundationOne ®" for solid tumors[37]. Finally, current service has been implemented for detection of Microsatellite Instability (MSI) and Tumor Mutational Burden (TMB).

Based on the above synergistic effects, Atezolizumab plus Bevacizumab might provide significant TMB selected NSCLC patients. For this reason, the Fundación GECP finds of great interest the following protocol: A phase 2 study evaluating the efficacy of Atezolizumab in combination with Bevacizumab as first line therapy for advanced or metastatic high-intermediate TMB (TMB≥10 mutations/MB analyzed in tumor or TMB≥16 analyzed in blood) non-squamous NSCLC patients.

Objectives and endpoints:

Primary objective:

- To evaluate the efficacy of Atezolizumab in combination with Bevacizumab as measured by Progression Free Survival (PFS) at 12 months according to Response Evaluation Criteria in Solid Tumors (RECIST) Version 1.1.

Secondary objectives:

- To evaluate the ORR of Atezolizumab in combination with Bevacizumab as measured by investigator-assessed overall response rate (ORR) according to RECIST v1.1.
- To evaluate the efficacy of Atezolizumab in combination with Bevacizumab as measured by investigator-assessed duration of response (DOR) according to RECIST v1.1.
- To evaluate the efficacy of Atezolizumab in combination with Bevacizumab as measured by investigator-assessed time to response (TTR) according to RECIST v1.1.
- To evaluate the efficacy of Atezolizumab in combination with Bevacizumab as measured by investigator-assessed ORR, PFS and DOR according to immune-related response criteria; irRC.
- To evaluate the efficacy of Atezolizumab in combination with Bevacizumab as measured by investigator ORR, PFS and DOR according to immune-related response criteria (irRC) in patients with TMB ≥16 mutations/MB.
- To evaluate the PFS rate at 1 and 2 years of Atezolizumab in combination with Bevacizumab.
- To evaluate the OS rate at 1 and 2 years of Atezolizumab in combination with Bevacizumab.

- To evaluate ORR and PFS according PD-L1 expression of Atezolizumab in combination with Bevacizumab.
- To evaluate the safety and tolerability of Atezolizumab in combination with Bevacizumab.
- To evaluate patient-reported outcomes (PROs) of lung cancer symptoms, patient functioning, and health-related quality of life (HRQoL) as measured by the European Organisation for Research and treatment of Cancer (EORTC) Quality-of-life Questionnaire Core 30 (QLQ C30) and its Lung Cancer Module (QLQ LC13) of Atezolizumab in combination with Bevacizumab.

Exploratory Objectives

Foundation one:

- To determine the tumor mutational burden (TMB) and determine its association with all 4 types of genomic alteration (Indels, mutation, CNV, rearrangement) in 324 tumor related genes, MSI and determine by FoundationOne CDx™ (F1CDx), and other biomarkers such as tumor PD-L1 expression.
- To investigate genomics alteration change measure in blood after progression measured with FoundationOne®Liquid, in respect with genomics alteration measure by FoundationOne CDx™ (F1CDx) in tissue including genomics signatures (MSI and TMB) at progression.
- To evaluate the clinical utility of the TMB results/reports in patients with TMB<10, mutations/MB describing druggable alterations or driver mutation that influence treatment selection.
- To evaluate overall survival (OS) and ORR of atezolizumab in combination with bevacizumab based on the TMB analysis in blood and in tumor)

Additional pharmacogenomic exploratory analysis:

- To evaluate the basal peripheral blood TCR and BCR repertoire and their changes during treatment, as well as, their correlation with clinical variables associated with treatment efficacy (ORR, PFS, OS, DOR).
- To study the levels of peripheral blood immune cells and soluble factors from plasma samples considering their changes during treatment, as well as, their correlation with clinical variables associated with treatment efficacy (ORR, PFS, OS, DOR).
- To determine the pre-treatment fecal microbiome of all patients included in the study and its predictive value in terms of ORR, PFS, OS and DOR.

Eligibility criteria:

Inclusion criteria:

1. Male or female, aged ≥ 18 years old
2. ECOG performance status of 0 or 1.

3. Histologically or cytologically confirmed, Stage IIIB - IV non-squamous NSCLC according to 8th version of the International Association for the Study of Lung Cancer Staging Manual in Thoracic Oncology
4. No prior treatment for Stage IIIB - IV non-squamous NSCLC.
5. Patients who have received prior neo-adjuvant, adjuvant chemotherapy, radiotherapy, or chemo-radiotherapy with curative intent for non-metastatic disease must have experienced a treatment-free interval of at least 6 months from enrollment since the last chemotherapy, radiotherapy, or chemo-radiotherapy.
6. Patients with a treated asymptomatic CNS metastasis are eligible, provided they meet all of the following criteria:
    a. Only supratentorial and cerebellar metastases allowed (i.e., no metastases to midbrain, pons, medulla or spinal cord).
    b. No ongoing requirement for corticosteroids as therapy for CNS disease.
    c. No stereotactic radiation within 7 days or whole-brain radiation within 14 days prior to enrollment.
    d. No evidence of interim progression between the completion of CNS-directed therapy and the screening radiographic study.

    Patients with new asymptomatic CNS metastases detected at the screening scan must receive radiation therapy and/or surgery for CNS metastases. Following treatment, these patients may then be eligible without the need for an additional brain scan prior to inclusion, if all other criteria are met.
7. Patients with high-intermediate Tumor Mutational Burden analyzed by Foundation Medicine (≥10 mutations/ MB) performed by a Foundation Medicine laboratory on previously obtained archival tumor tissue or tissue obtained from a biopsy at pre-screening (sample must fulfil minimal sample requirements of 20% tumor cellularity and a minimum surface of 25mm^2). TMB≥16 mutations/ MB is required if the analysis is performed in blood sample.
8. Measurable disease, as defined by RECIST v1.1.

    Previously irradiated lesions can only be considered as measurable disease if disease progression has been unequivocally documented at that site since radiation and the previously irradiated lesion is not the only site of disease.
9. Adequate hematologic and organ function defined by the following laboratory results obtained within 14 days prior to enrollment:
    - Neutrophils ≥ 1500 cells/μL without granulocyte colony-stimulating factor support.
    - Lymphocyte count ≥ 500/μL.
    - Platelet count ≥ 100,000/μL without transfusion.
    - Haemoglobin ≥ 9.0 g/dL. Patients may be transfused to meet this criterion.
    - INR or aPTT ≤ 1.5 × upper limit of normal (ULN). This applies only to patients who are not receiving therapeutic anticoagulation; patients receiving therapeutic anticoagulation should be on a stable dose.
    - AST, ALT, and alkaline phosphatase ≤ 2.5 × ULN, with the following exceptions:

        Patients with documented liver metastases: AST and/or ALT ≤ 5 × ULN.

        Patients with documented liver or bone metastases: alkaline phosphatase ≤ 5 × ULN.
    - Serum bilirubin ≤ 1.25 × ULN. Patients with known Gilbert disease who have serum bilirubin level ≤ 3 × ULN may be enrolled.
    - Serum creatinine ≤ 1.5 × ULN or creatinine clearance of ≥45ml/min (based on the Cockcroft Gault formula).

10. All patients are notified of the investigational nature of this study and signed a written informed consent in accordance with institutional and national guidelines, including the Declaration of Helsinki prior to any trial-related intervention.
11. For female patients of childbearing potential, agreement (by patient and/or partner) to use a highly effective form(s) of contraception that results in a low failure rate (< 1% per year) when used consistently and correctly, and to continue its use for 5 months after the last dose of Atezolizumab and/or 6 months after the last dose of Bevacizumab, whichever is later. Such methods include: combined (oestrogen and progestogen containing) hormonal contraception, progestogen-only hormonal contraception associated with inhibition of ovulation together with another additional barrier method always containing a spermicide, intrauterine device (IUD): intrauterine hormone-releasing system (IUS), bilateral tubal occlusion, vasectomized partner (on the understanding that this is the only one partner during the whole study duration), and sexual abstinence.
12. For male patients with female partners of childbearing potential, agreement (by patient and/or partner) to use a highly effective form(s) of contraception that results in a low failure rate [< 1% per year] when used consistently and correctly, and to continue its use for 6 months after the last dose of Bevacizumab. Male patients should not donate sperm during this study and for at least 6 months after the last dose of Bevacizumab.
13. Oral contraception should always be combined with an additional contraceptive method because of a potential interaction with the study drugs. The same rules are valid for male patients involved in this clinical study if they have a partner of childbirth potential. Male patients must always use a condom.
14. Women who are not postmenopausal (≥ 12 months of non−therapy-induced amenorrhea) or surgically sterile must have a negative serum pregnancy test result within 8 days prior to initiation of study drug.

Exclusion criteria:

Cancer-Specific Exclusions:

1. Patients with a sensitizing mutation or an amplification in the epidermal growth factor receptor (EGFR) gene.
2. Patients with an anaplastic lymphoma kinase (ALK) fusion oncogene.
3. Patients with an STK-11 Ligand alteration.
4. Patients with MDM2 amplification.
5. Patients with ROS1 translocations.
6. Active or untreated CNS metastases as determined by CT or magnetic resonance imaging (MRI) evaluation during screening and prior radiographic assessments.
7. Spinal cord compression not definitively treated with surgery and/or radiation or previously diagnosed and treated spinal cord compression without evidence that disease has been clinically stable for > 2 weeks prior to enrollment.
8. Leptomeningeal disease.
9. Uncontrolled tumor-related pain.

    Patients requiring pain medication must be on a stable regimen at study entry.
    Symptomatic lesions amenable to palliative radiotherapy (e.g., bone metastases or metastases causing nerve impingement) should be treated prior to initiation of study drug.

Patients should be recovered from the effects of radiation. There is no required minimum recovery period.

Asymptomatic metastatic lesions whose further growth would likely cause functional deficits or intractable pain (e.g., epidural metastasis that is not currently associated with spinal cord compression) should be considered for locoregional therapy, if appropriate, prior to initiation of study drug.

10. Uncontrolled pleural effusion, pericardial effusion, or ascites requiring recurrent drainage procedures (once monthly or more frequently).

    Patients with indwelling catheters (e.g., PleurX®) are allowed.

11. Uncontrolled or symptomatic hypercalcemia (> 1.5 mmol/L ionized calcium or Ca > 12 mg/dL or corrected serum calcium > ULN).
12. Malignancies other than NSCLC within 5 years prior to enrollment, with the exception of those with a negligible risk of metastasis or death (e.g., expected 5-year OS > 90%) treated with expected curative outcome (such as adequately treated carcinoma in situ of the cervix, basal or squamous-cell skin cancer, localized prostate cancer treated with radiotherapy or surgically with curative intent, ductal carcinoma in situ treated surgically with curative intent).

General Medical Exclusions:

13. Women who are pregnant, lactating, or intending to become pregnant during the study.
14. History of severe allergic, anaphylactic, or other hypersensitivity reactions to chimeric or humanized antibodies or fusion proteins.
15. Known hypersensitivity or allergy to biopharmaceuticals produced in Chinese hamster ovary cells or any component of the Atezolizumab formulation.
16. History of autoimmune disease, including but not limited to myasthenia gravis, myositis, autoimmune hepatitis, systemic lupus erythematosus, rheumatoid arthritis, inflammatory bowel disease, vascular thrombosis associated with antiphospholipid syndrome, Wegener's granulomatosis, Sjögren's syndrome, Guillain-Barré syndrome, multiple sclerosis, vasculitis, or glomerulonephritis.

    Patients with a history of autoimmune-related hypothyroidism on a stable dose of thyroid replacement hormone are eligible for this study.

    Patients with controlled Type 1 diabetes mellitus on a stable dose of insulin regimen are eligible for this study.

    Patients with eczema, psoriasis, lichen simplex chronicus, or vitiligo with dermatologic manifestations only (e.g., patients with psoriatic arthritis would be excluded) are permitted provided that they meet the following conditions:

    - Rash must cover less than 10% of body surface area (BSA).
    - Disease is well controlled at baseline and only requiring low-potency topical steroids.
    - No acute exacerbations of underlying condition within the previous 12 months (not requiring PUVA [psoralen plus ultraviolet A radiation], methotrexate, retinoids, biologic agents, oral calcineurin inhibitors, high-potency or oral steroids).

17. History of idiopathic pulmonary fibrosis, organizing pneumonia (e.g., bronchiolitis obliterans), drug-induced pneumonitis, idiopathic pneumonitis, or evidence of active pneumonitis on screening chest CT scan.

History of radiation pneumonitis in the radiation field (fibrosis) is permitted.

18. Positive test for HIV. All patients will be tested for HIV prior to inclusion into the study; patients who test positive for HIV will be excluded from the clinical study.
19. Patients with active hepatitis B (chronic or acute; defined as having a positive hepatitis B surface antigen [HBsAg] test at screening) or hepatitis C.
    Patients with past hepatitis B virus (HBV) infection or resolved HBV infection (defined as the presence of hepatitis B core antibody [HBcAb] and absence of HBsAg) are eligible only if they are negative for HBV DNA (vaccinated patients are excluded).
    Patients positive for hepatitis C virus (HCV) antibody are eligible only if PCR is negative for HCV RNA.
20. Active tuberculosis.
21. Severe infections within 4 weeks prior to be included in the study, including but not limited to hospitalization for complications of infection, bacteraemia, or severe pneumonia.
22. Received therapeutic oral or IV antibiotics within 2 weeks prior to be included in the study.
    Patients receiving prophylactic antibiotics (e.g., for prevention of a urinary tract infection or to prevent chronic obstructive pulmonary disease exacerbation) are eligible.
23. Significant cardiovascular disease, such as New York Heart Association cardiac disease (Class II or greater), myocardial infarction, or cerebrovascular accident within 3 months prior to inclusion, unstable arrhythmias, or unstable angina.
    Patients with known coronary artery disease, congestive heart failure not meeting the above criteria, or left ventricular ejection fraction < 50% must be on a stable medical regimen that is optimized in the opinion of the treating physician, in consultation with a cardiologist if appropriate.
24. Major surgical procedure other than for diagnosis within 28 days prior to inclusion or anticipation of need for a major surgical procedure during the course of the study.
25. Prior allogeneic bone marrow transplantation or solid organ transplant.
26. Administration of a live, attenuated vaccine within 4 weeks before inclusion or anticipation that such a live attenuated vaccine will be required during the study.
27. Any other diseases, metabolic dysfunction, physical examination finding, or clinical laboratory finding giving reasonable suspicion of a disease or condition that contraindicates the use of an investigational drug or that may affect the interpretation of the results or renders the patient at high risk from treatment complications.
28. Patients with illnesses or conditions that interfere with their capacity to understand follow and/or comply with study procedures.

Exclusion Criteria Related to Medications:

29. Any approved anti-cancer therapy, including hormonal therapy, within 3 weeks prior to initiation of study treatment.
30. Treatment with any other investigational agent with therapeutic intent within 28 days prior to initiation of study treatment.
31. Treatment with systemic immunosuppressive medications (including but not limited to corticosteroids, cyclophosphamide, azathioprine, methotrexate, thalidomide, and anti−tumor necrosis factor [anti-TNF] agents) within 2 weeks prior to inclusion.
    Patients who have received acute, low-dose (≤ 10 mg oral prednisone or equivalent), systemic immunosuppressant medications may be enrolled in the study.

The use of corticosteroids (≤ 10 mg oral prednisone or equivalent) for chronic obstructive pulmonary disease, mineralocorticoids (e.g., fludrocortisone) for patients with orthostatic hypotension, and low-dose supplemental corticosteroids for adrenocortical insufficiency is allowed.

Exclusions Related to Bevacizumab:

32. Inadequately controlled hypertension (defined as systolic blood pressure > 150 mmHg and/or diastolic blood pressure > 100 mmHg).
    Anti-hypertensive therapy to achieve these parameters is allowable.
33. Prior history of hypertensive crisis or hypertensive encephalopathy.
34. Significant vascular disease (e.g., aortic aneurysm requiring surgical repair or recent peripheral arterial thrombosis) within 6 months prior to inclusion.
35. History of haemoptysis (≥ one-half teaspoon of bright red blood per episode) within 1 month prior to inclusion.
36. Evidence of bleeding diathesis or coagulopathy (in the absence of therapeutic anticoagulation).
37. Current or recent (within 10 days of inclusion) use of aspirin (> 325 mg/day) or treatment with dipyramidole, ticlopidine, clopidogrel, and cilostazol.
38. Current use of full-dose oral or parenteral anticoagulants or thrombolytic agents for therapeutic purposes that has not been stable for > 2 weeks prior to inclusion.
    The use of full-dose oral or parenteral anticoagulants is permitted as long as the INR or aPTT is within therapeutic limits (according to the medical standard of the enrolling institution) and the patient has been on a stable dose of anticoagulants for at least 2 weeks prior to inclusion.
    Prophylactic anticoagulation for the patency of venous access devices is allowed, provided the activity of the agent results in an INR < 1.5 × ULN and aPTT is within normal limits within 14 days prior to inclusion.
    Prophylactic use of low-molecular-weight heparin (i.e., enoxaparin 40 mg/day) is permitted.
39. Core biopsy or other minor surgical procedure, excluding placement of a vascular access device, within 7 days prior to the first dose of Bevacizumab.
40. History of abdominal or tracheoesophageal fistula or gastrointestinal perforation within 6 months prior to inclusion.
41. Clinical signs of gastrointestinal obstruction or requirement for routine parenteral hydration, parenteral nutrition, or tube feeding.
42. Evidence of abdominal free air not explained by paracentesis or recent surgical procedure.
43. Serious, non-healing wound, active ulcer, or untreated bone fracture.
44. Proteinuria, as demonstrated by urine dipstick or > 1.0 g of protein in a 24-hour urine collection.
    All patients with ≥ 2+ protein on dipstick urinalysis at baseline must undergo a 24-hour urine collection and must demonstrate ≤ 1 g of protein in 24 hours.
45. Known sensitivity to any component of Bevacizumab.
46. Clear tumor infiltration into the thoracic great vessels is seen on imaging.
47. Clear cavitation of pulmonary lesions is seen on imaging.

Treatment:

Atezolizumab: 1200mg, IV infusion
Bevacizumab: 15 mg/kg, IV infusion

The treatment will start within 1-5 days from enrollment. The treatment will be administered at 21-day intervals (3 weeks ±3days). On Day 1 of each cycle, all eligible patients will receive drug infusions in the following order:

Atezolizumab → Bevacizumab

Atezolizumab may continue beyond disease progression per RECIST v1.1 until loss of clinical benefit, unacceptable toxicity, patient or physician decision to discontinue, or death. If the principal investigator would like to continue beyond progression a permission from the trial chair/sponsor have to be granted. For all patients, tumor response data collection will continue until disease progression, even if the patient stops study treatment prior to disease progression.

Bevacizumab will be administered until progression disease, unacceptable toxicity, patient or physician decision to discontinue or death.

Statistical considerations:

The primary endpoint is Progression Free Survival (PFS) at 12 months according to Response Evaluation Criteria in Solid tumors (RECIST) v1.1.

For one arm, we estimate to achieve a PFS at 12 months of 40% (vs 18% from Chemotherapy, previous published studies), with a 90% power and alpha 5%, one-sided test. The test statistic for survival probability is based on the non-parametric estimate of the survival distribution. Additionally, estimating a 10% of errors, withdrawals or others, it will be necessary to recruit 40 patients in order to get the study objectives

If the withdrawals are higher than 5%, so that in order to reach the proposed sample size, if a patient initially enrolled in the study does not fulfil the inclusion criteria, they will be replaced by a new one subject that fulfil them, this replacement will ensure that the sample size will be the one calculated initially.

<u>Data analysis and statistical methods</u>

A descriptive analysis will be made of the variables calculating absolute and relative frequencies of qualitative variables and mean (standard deviation) or median (25 and 75 percentiles), minimum and maximum values for quantitative variables.

For the primary endpoint analysis, survivor function estimated by Kaplan-Meier method will be used, as well as the survival curves. The 95% confidence interval will be estimated.

For the bivariate analysis, appropriate parametric and nonparametric tests will be used depending on whether the data are normally or non-normally distributed. For comparison of quantitative variables, Student's t test for independent data, the Mann-Whitney U test, analysis of variance or the Kruskal-Wallis H test will be used.

Total trial duration: 4.5 years (1.5 years of recruitment, approximately 1.5 years of treatment and 1.5 years of follow up)

Translational research:

Before enrollment, tissue samples will be analyzed by different methods as IHC and FoundationOne CDx™ (F1CDx). These exploratory analyses aim to study tumor-associated alterations to further understand disease pathobiology (including but not limited to mechanisms of disease progression, pseudo-progression, acquired resistance), to evaluate surrogate biomarkers and to potentially allow for the development of tissue-based diagnostic tests to help predict which patients may benefit from Atezolizumab in combination with Bevacizumab vs Atezolizumab alone. If the submitted tumor sample is insufficient or not valid and a biopsy is not feasible to be performed to evaluate the TMB, a blood sample can be sent instead.
A permission from the trial chair/sponsor must be requested in case no tumor sample is available for a possible patient and blood sample want to be sent to analyze the TMB.

After enrollment, blood and stool samples will be send to the central laboratory at Hospital Puerta de Hierro de Majadahonda to perform additional pharmacogenomic exploratory analysis (see section 3.6)

2. Trial schedule

	Pre-Screening	Screening/ Baseline (-28 days before enrollment)	Bevacizumab and Atezolizumab Cycles[16]	Atezolizumab Cycles (beyond progression disease)[17]	End of Treatment[18]	Follow up[19]
Informed consent[1]	X	X				
Medical history and demographic data		X				
Physical examination:<br>Height<br>Performance status (ECOG)<br>Weight, Temperature, Blood pressure		X<br>X<br>X	X<br>X[16]	X<br>X		X<br>X<br>First follow up
Hematology[2, 3]		X	X	X		X<br>First follow up
Biochemistry[2, 4] (Including renal and hepatic function)		X	X	X		X<br>First follow up
Coagulation[2, 5]		X	X	X		X<br>First follow up
Thyroid function[2, 6]		X	X<br>At day 1 of Cycle 2 and every 2 cycles	X<br>Every 2 cycles		X<br>First follow up
HIV / Hepatitis B/C[7]		X				
Pregnancy test[8]		X	X<br>At day 1 of Cycle 2 and every 2 cycles	X<br>Every 2 cycles		X[8]<br>First follow up
Urine dipstick[9]		X	X			X<br>First follow up
12-lead ECG		X				X<br>First follow up
Adverse events[10] (Toxicity evaluation)		X<br>To evaluate baseline status	Continuous	Continuous	Continuous	X<br>First follow up[10]
Concomitant medications[11]		X	Continuous	Continuous	Continuous	X<br>First follow up
Tumor assessment[12]		X	X	X		X[12]
Patient Reported Outcomes (PROs): QLQ30-LC13[13]		X	X<br>At day 1 of Cycle 3 and every 3 cycles		X[13]	X[13]
Blood sample (Foundation one)	X[15]				X[14]	X[14]
FFPE tumor material (Foundation one)[15]	X					
Blood samples (pharmacogenomic exploratory analysis)[20]		X	X			X
Stool sample collection[21]		X				

[1] Before any trial specific evaluations or interventions. The patient has to sign a specific pre-screening informed consent to send the tumor block for pre-screening. If the patient has a TMB ≥10 analyzed in tumor or TMB ≥16 analyzed in blood, then the patient will sign the Informed consent (IC) of the study

[2] Laboratory testing prior to each dose administration: within 14 days in baseline visit and within 3 days prior to day 1 administration of each cycle. If baseline testing is performed more than 3 days before first study dose, they must be repeated on day 1 cycle 1.

[3] *Hematology*: WBC count, neutrophils, haemoglobin, platelet count

[4] *Biochemistry*: Ca, Mg, Na, K, Glucose, ALT, AST, LDH, total bilirubin, alkaline phosphatase, creatinine and clearance of creatinine calculated by Cockcroft-Gault formula, total protein, albumin, amylase, lipase.

[5] *Coagulation*: PT, INR and APTT

[6] *Thyroid function:* thyroid-stimulating hormone (TSH). Total or free T3 and total or free T4 must be analyzed if TSH result is out of ULN or LLN.

[7] *HIV / Hepatitis B/C:* The following baseline local laboratory assessments should be done within 28 days prior to first dose: Hepatitis B and C testing (HBV sAg, HBV Ab and HCV Ab) and HIV test. In patients with positive hepatitis B/C results, HBV DNA/HCV RNA must be performed (Hepatitis B vaccinated patients are excluded).

[8] *Pregnancy Tests:* A serum or urine pregnancy testing is required within 3 days prior to study enrollment, then every 6 weeks and within 30 days of last treatment dose (more frequently if required by local standard).

[9] *Urine test*: A urine testing is required for proteinuria within 3 days prior to study enrollment and at day 1 of each cycle of Bevacizumab and Atezolizumab (+/- 3 days).

[10] *Adverse events* must be recorded and followed from the day of the signs the patient Informed consent for the study to 30 days from last dose of administration. SAEs and AESIs must be recorded and followed from the subject written consent to 30 days from last dose of administration.

[11] *Concomitant medications* must be recorded and followed from 7 days prior to day 1 cycle 1 to 30 days from last dose of administration.

[12] *Tumor assessment:*

- **Chest and abdomen CT SCAN** with oral/IV contrast (unless contraindicated) must be done at baseline within 28 days before enrollment (+ 12 days). During treatment the tumor assessments will be done every 12 weeks (+/- 7 days) from cycle 1 day 1. CT SCAN must be performed until progression disease or until last dose of Atezolizumab in case of continuing beyond progression. The CT SCAN schedule will be maintained even if a delay in administration occurs.
- **An MRI or CT brain** with oral/IV contrast (unless contraindicated) must be done at baseline within 28 days before enrollment (+ 12 days).
- A full-body PET-TAC, pelvis or the neck CT scan and Bone scans should be performed if clinically indicated at baseline and at subsequent response evaluations.

Note that it is not mandatory to perform the tumor assessment in first follow up visit if the end of treatment reason is other than progression disease and a tumor assessment is been performed in the previous 12 weeks.

During follow up, tumor assessment will be maintained every 12 weeks (+/- 7 days) in case end of treatment reason is other than disease progression. Tumor assessments must be performed until progression disease.

[13] *PROs (QLQ30-LC13)* must be performed at:

- Baseline
- Day 1 of Cycle 3 and every 3 cycles until discontinuation of treatment

- At progression disease, when it occurs.

In the event of a delay in administration, the PROs schedule will be also delayed to be performed on day 1 of the specified cycles.

Even if the progression disease occurs within 9 weeks after of the last completed PRO, the progression PRO must be performed.

[14] Foundation one blood samples will be drawn at progression (two 10 ml tubes).

[15] Tumor sample is required for pre-screening before enrollment. A minimum of 10 slides 4-5 um thick, with at least 20% tumor cellularity will be necessary for FoundationOne CDx™ (F1CDx) Panel. If the submitted tumor sample is insufficient or not valid and a biopsy is not feasible to be performed to evaluate the TMB, a blood sample can be sent instead. A permission from the trial chair/sponsor must be requested in case no tumor sample is available for a possible patient, and blood sample want to be sent to analyze the TMB.

[16] Cycles will be administered every 3 weeks (±3 days) until progression or other reason to discontinue. Day 1 of cycle 1 treatment will start within 1-5 days from enrollment. If a patient must stop one of the two drugs due to toxicity it is allowed to continue treatment with the other drug as long as patient does not present progression disease. If progression disease is shown is only permitted continue treatment beyond progression with atezolizumab.

Blood pressure must be taken always before the drug administration. Blood pressure should be routinely measured before every infusion after a resting period of 10 min. If baseline record is performed more than 3 days before first study dose, they must be repeated on day 1 cycle 1

[17] Atezolizumab may continue beyond disease progression per RECIST v1.1 until loss of clinical benefit, unacceptable toxicity, patient or physician decision to discontinue, or death. If the principal investigator would like to continue beyond progression a permission from the trial chair/sponsor have to be granted.

[18] *End of treatment* is defined as the day of discontinuation of the treatment.

[19] A first follow up visit must be done 30 days (±3 days) after the last dose of treatment (Bevacizumab +Atezolizumab or Atezolizumab beyond progression) or immediately before the initiation of any other anticancer therapy.
The subsequent follow up visits can be matched at least with the CT SCAN schedule or more frequently as per local standard.

[20] Pharmacogenomic exploratory analysis blood samples will be drawn at:

- Baseline or before day 1 of cycle 1 treatment
- First CT-scan evaluation (cycle 5 if there is no delay in treatment administration)
- Second CT-scan evaluation (cycle 9 if there is no delay in treatment administration)
- Fourth CT-scan evaluation (cycle 17 if there is no delay in treatment administration)
- Sixth CT-scan evaluation (cycle 25 if there is no delay in treatment administration)
- Progression disease, either if it occurs during the bevacizumab + atezolizumab treatment or in follow up phase

[21] Stool samples will be collected at baseline.

3. Background and rationale

3.1. Disease background and treatments in advanced NSCLC patients

Lung cancer is the leading cause of cancer-related death in industrialized countries[1]. Non-small cell lung cancer (NSCLC) accounts for 85% of cases of lung cancer and is categorized into a variety of histological subtypes being non-squamous tumors the most common subtype[2].

Despite recent improvements in treatment, the prognosis for patients with advanced NSCLC remains dismal, with a median OS of approximately 12.5 months[3].

Inhibition of programmed cell death protein-ligand 1/programmed cell death protein 1 (PD-L1/PD-1) signalling has been shown to induce durable responses in some patients with different tumors, and expression of PD-L1 by tumor cells (TCs) in several tumor types (including NSCLC) correlates with response to therapy[4]. Immunotherapy has demonstrated that therapies focused on enhancing T-cell responses against cancer may result in a significant survival benefit in patients with Stage IV cancer[5,6,7].

Immunotherapeutic targeting of PD-L1 or PD-1 is revolutionizing the treatment of metastatic NSCLC. Currently, two anti-PD-1 therapeutic antibodies (Pembrolizumab and Nivolumab) and one anti-PD-L1 (Atezolizumab) have been approved for NSCLC treatment. Both agents are recommended as second-line agents in advanced NSCLC after prior progression on platinum-containing chemotherapy.

Atezolizumab is a humanized anti-PD-L1 monoclonal antibody that inhibits its interaction with its receptors, PD-1 and B7.1 (CD80, B7-1), reinvigorating anticancer immunity. The OAK study evaluated the efficacy of Atezolizumab compared to Docetaxel in 1.225 patients previously treated with locally-advanced or metastatic NSCLC. The PD-L1 targeted therapy by Atezolizumab resulted in a clinically relevant improvement of overall survival *versus* Docetaxel regardless of PD-L1 expression or histology, with a favourable safety profile. The median OS in this study was 13.8 months (95% CI 11.8-15.7) in the Atezolizumab arm compared to 9.6 months (95% CI 8.6-11.2) in the Docetaxel arm (HR=0.73, 95% CI 0.62-0.87; p=0.0003)[8].

The GO28625 (NCT01846416, FIR) phase 2 study enrolled 137 PD-L1-selected NSCLC patients regardless previous treatments. The primary analysis for efficacy-evaluable patients, confirmed ORR after the first-line treatment with Atezolizumab was 28.6% (95% CI: 3.7-71.0) in IHC TC3 or IC3 patients and 25.8% (95% CI: 11.9-44.6) in IHC TC2/3 or IC2/3 patients[9].

The efficacy and safety of Atezolizumab was also evaluated in another phase 2 single-arm study where 142 PD-L1-selected advanced NSCLC patients (NCT02031458, BIRCH) were treated with Atezolizumab monotherapy. The primary endpoint ORR in the ITT population was 25%, in TC3 or IC3 subgroup was 34% and 18% in TC2 and IC2. Median PFS and OS for the entire population were 7.3 and 23.5 months respectively. These results indicate that Atezolizumab monotherapy has durable efficacy in the first line setting[10].

Currently, first-line treatment for advanced NSCLC without presence of genetic aberrations, such as sensitizing mutations of epidermal growth factor receptor (EGFR) or translocations of anaplastic lymphoma kinase (ALK), are chemotherapy and Pembrolizumab, approved based on KEYNOTE-024 study. This study randomized patients with advanced NSCLC and PD-L1 expression (at least 50% of tumor cells), showing median progression-free survival (PFS) of 10.3 months (95% CI 6.7-not reached) versus 6.0 months (95% CI 4.2-6.2) (HR 0.50; 95% CI 0.37-0.68; P<0.001) for the Pembrolizumab and chemotherapy groups respectively[11] .

Currently there is a clinical trial (NCT02409342, IMpower110) of Atezolizumab as first line monotherapy in PD-L1 selected NSCLC patients that is expected to add the first line approval to Roche´s portfolio. Thus, all the above data support the rational of cancer immunotherapy in first line PD-L1 positive NSCLC[12]. In addition, there is a growing body of evidence that suggests that pro-angiogenic factors can modulate the immune response and may serve as mechanisms of scape. Therefore, the combination of cancer immunotherapy with antiangiogenic agents makes sense.

The vascular endothelial growth factor (VEGF) is the most important pro-angiogenic protein and a key regulator of physiological angiogenesis. It is also implicated in pathological angiogenesis associated with tumor growth. Indeed, increased levels of VEGF have been found in lung cancer; moreover, the overexpression of VEGF is associated with a poor prognosis[13,14]. Actually, VEGF plays a role in cancer immune evasion through modification of the endothelial cells in the tumor microenvironment, where VEGF may reduce lymphocyte adhesion and migration from vessels to the tumor site contributing to decreased immune cell recruitment[15]. Given the immunosuppressive role of VEGF and angiogenesis within tumors, it is not surprising that there is evidence supporting how antiangiogenic agents stimulate the immune response and enhance the efficacy of immunotherapies[16].

Recent findings indicating an intertwined regulation of VEGF signalling and immunosuppression in the tumor microenvironment suggest that the combination of anti-VEGF agents and immune checkpoint blockade could have synergistic antitumor activity, along with favourable tolerability[16].

Bevacizumab is a recombinant, humanized therapeutic anti-VEGF antibody that inhibits tumor angiogenesis which may correct the immunosuppressive function exerted by VEGF, increasing the infiltration of T effector cells in cancer[17].

So far, the more advanced studies to address the combination of cancer immunotherapy (CIT)-antiangiogenic agents such as Bevacizumab, there is a phase 3 study of Paclitaxel, Carboplatin, Bevacizumab and Atezolizumab (NCT02366143, IMpower150). IMpower150 is the first phase III immunotherapy-based combination study to demonstrate a statistically significant and clinically meaningful improvement in PFS in all-comer 1L NSQ mNSCLC, providing a potential new standard of care for patients, showing a median PFS of 8.3 moths vs 6.8 moths (HR 0.62; 95% CI: 0.52, 0.74; P < 0.0001) for the Atezolizumab, chemotherapy and Bevacizumab arm (ABCP) and Carboplatin, Paclitaxel and Bevacizumab arm (BCP) respectively. In terms of PFS 12 months the results were 37% in the Atezolizumab arm vs 18% un the CT + Bevacizumab arm. Median overall survival among the patients was longer in the ABCP group than in the BCP group (19.2 months vs. 14.7 months; hazard ratio for death, 0.78; 95% CI, 0.64 to 0.96; P = 0.02)[18].

The phase 3 study of Atezolizumab in combination with Bevacizumab compared to Sunitinib single agent (IMmotion151), as first-line therapy in untreated metastatic Renal Cell Carcinoma (RCC) patients. This clinical trial showed a significant benefit of 3.5 months in the PFS favouring the CIT-Bevacizumab combination (11.2 m vs. 7.7 m) with a tolerable safety profile in PD-L1+ patients[19].

These data confirm the role of Bevacizumab as an immune modulating agent that may provide a successful combination strategy in different tumor types.

Increased mutation rate is a well-characterized feature of human cancer. Abnormal activity in several cellular pathways, including DNA damage repair and DNA replication, can increase the overall rate of somatic mutations in tumors, as can exposure to mutagens such as ultraviolet light and tobacco smoke[20]. Defects in DNA damage repair lead to the accumulation of mutations caused by replicative errors and environmental damage[21].

The core DNA mismatch repair protein complex is composed of two cooperative dimers: the PMS2 protein dimerizes with MLH1 to form the complex MutL-alpha, which cooperates with the MSH2-MSH6 dimer, MutS-alpha, to repair single base pair mismatches and small insertion–deletion loops[22]. PMS2 promoter mutations are present in ~10% of melanoma samples and ~8% of squamous cell carcinomas[23].

Perturbations in mismatch repair gene expression, both loss and overexpression, can be deleterious to genomic stability, and loss of function mutations in mismatch repair pathway genes are known to correlate with high Tumor Mutational Burden (TMB) in tumors, overall mutations in these genes accounted for less than 10% of cases with high TMB[23].

As such, tumors with defective DNA repair mechanisms are more likely to benefit from immunotherapy. DNA replication is another key pathway in which defects can lead to increased somatic mutation rate.

Loss of function mutations in TP53 are very common in cancer and are a somatic marker of elevated mutation rate[24]. Mutations in a number of other genes have also been linked to increase TMB. KRAS-mutant lung cancers account for approximately 25% of non-small cell lung carcinomas, thus representing an enormous burden of cancer worldwide. KRAS mutations are clear drivers of tumor growth and are characterized by a complex biology involving the interaction between mutant KRAS, various growth factor pathways, and tumor suppressor genes. KRAS mutations are classically associated with a significant smoking history[25]. Then, it is an emerging biomarker for response to immunotherapy. The CheckMate 026 trial is a randomized trial comparing the treatment efficacy of Nivolumab versus chemotherapy in first line therapy of advanced non-small cell lung cancer based on the programmed death-ligand 1 expression. However, there was no strong correlation between treatment response and PD-L1 expression. Therefore, researchers performed a sub analysis to assess if the TMB could determine Nivolumab treatment response in 312 patients with evaluable TMB data. The results demonstrated that patients with a high mutation burden had a better progression free survival (9.7 vs 5.8 months; HR, 0.62) and objective response rate (46.8% vs 28.3%) compared with the control arm. These findings imply that tumor mutation burden may be considered as a biomarker for immunotherapy[26].

Regarding TMB data, the CheckMate227 study is an open-label, phase 3 trial, that examined

progression-free survival with nivolumab plus ipilimumab versus chemotherapy among patients with stage IV or recurrent NSCLC that was not previously treated with chemotherapy with a high tumor mutational burden (≥10 mutations per megabase). Median PFS among patients with a high tumor mutational burden was significantly longer with nivolumab plus ipilimumab than with chemotherapy, 7.2 months (95% confidence interval [CI], 5.5 to 13.2) versus 5.5 months (95% CI, 4.4 to 5.8) (HR, 0.58; 97.5% CI, 0.41 to 0.81; P<0.001)[27].

Unlike the majority of genetic panels used within the standard of practice in oncology, even those using Next Generation Sequencing (NGS), Foundation Medicine ® (FMI) is a "Comprehensive Genomic Profiling" test, able to detect all cancer-relevant genes with all classes of alterations across the entire coding region, being also a validated performance[36]. Moreover, is a hybridization capture-based NGS method which, contrary to the more common Hot Spot Multi-gene panels, do not miss any relevant gene or genetic alteration[37]. Besides, FMI has two formats, both of them validated in top tier peer-reviewed journal: "FoundationOne ®" for solid tumors[37]. Finally, current service has been implemented for detection of Microsatellite Instability (MSI) and Tumor Mutational Burden (TMB).

3.2. Immunotherapy

Several studies in patients with NSCLC suggested an association of increased immune cell infiltration into tumors with improved survival. In recent years, improved identification of antigenic targets, the addition of immunoadjuvants, and the production of more efficient delivery systems have resulted in more efficient vaccines, able to elicit a potent immune response, leading to the development of immunotherapy for the treatment of NSCLC[28].

3.3. Background on Atezolizumab

Atezolizumab (MPDL3280A) is a humanized immunoglobulin (Ig) G1 monoclonal antibody consisting of two heavy chains (448 amino acids) and two light chains (214 amino acids) and is produced in Chinese hamster ovary cells. Atezolizumab was engineered to eliminate Fc-effector function via a single amino acid substitution on position 298 of the heavy chain, which results in a non-glycosylated antibody that has minimal binding to Fc receptors and prevents Fc-effector function at expected concentrations in humans.

Atezolizumab blocks the interaction between Programmed death-ligand 1 (PD-L1 or B7-H1) and Programmed death-1 (PD-1) and B7.1 (CD80), both of which are negative regulators of T-lymphocyte activation[29]. Binding of PD-L1 to its receptors suppresses T-cell migration, proliferation and secretion of cytotoxic mediators and restricts tumor cell killing. Blocking PD-L1 enhances anticancer immunity by restoring antitumor T-cell activity and T-cell priming.

Clinical Pharmacokinetics and Immunogenicity

On the basis of available preliminary PL data (0.03-20 mg/kg), atezolizumab appeared to show linear pharmacokinetics at doses ≥ 1mg/kg. For the 1-mg/kg and 20-mg/kg dose groups, the mean apparent clearance and the mean volume of distribution under steady-state conditions had a range of 3.11 to 4.14 mL/kg and 48.1 to 67.0 mL/kg, respectively, which is consistent with the expected profile of an IgG1 antibody in humans.

The development of anti-therapeutic antibodies (ATAs) has been observed in patients in all dose cohorts and was associated with changes in pharmacokinetics for some patients in the lower dose cohorts (0.3, 1, and 3 mg/kg). The development of detectable ATAs has not had a significant impact on pharmacokinetics for doses from 10 to 20 mg/kg. Patients dosed at the 10-, 15- and 20-mg/kg dose levels have maintained the expected target trough levels of drug despite detection of ATAs. To date, no clear relationship among ATAs detection and adverse events or infusion reactions or efficacy has been observed.

Summary of Nonclinical Studies

The nonclinical strategy of the atezolizumab program was to demonstrate in vitro and in vivo activity, to determine in vivo pharmacokinetic (PK) behaviour, to demonstrate an acceptable safety profile and to identify a Phase I starting dose. The safety, pharmacokinetics and toxicokinetics of atezolizumab were investigated in mice and cynomolgus monkeys to support intravenous (IV) administration and to aid in defining the appropriate starting dose in humans. The nonclinical pharmacokinetics and toxicokinetics observed for atezolizumab supported entry into clinical studies and were consistent with the anticipated pharmacologic activity of down-modulating the PD-L1/PD-1 pathway.

Summary of Clinical Studies

Safety and efficacy data are summarized below from the following studies:

- Study PCD4989g: A Phase Ia, multicentre, first-in-human, open-label, dose-escalation study evaluating the safety, tolerability, immunogenicity, PK, exploratory pharmacodynamics and preliminary evidence of biologic activity of atezolizumab administered as a single agent by IV infusion every 3 weeks (q3w) to patients with locally advanced or metastatic solid malignancies or hematologic malignancies.
- Study GO28753 (POPLAR): A randomized, Phase II, open-label study assessing the clinical benefit of atezolizumab as a single agent versus docetaxel in PD-L1 unselected patients with locally advanced or metastatic NSCLC that has progressed during or following treatment with a platinum-containing regimen.
- Study OAK: A randomized, Phase III, open-label study assessing the clinical benefit of atezolizumab as a single agent versus docetaxel in PD-L1 unselected patients with previously treated advanced NSCLC that has progressed during or following treatment with a platinum-containing regimen.
- Study GP28328: A Phase Ib study of the safety and pharmacology of atezolizumab administered with bevacizumab and/or with chemotherapy in patients with advanced solid tumors.

Currently atezolizumab is approved by FDA and EMA in patients with advanced NSCLC who progressed to previous therapies including platinum-based chemotherapy.

Atezolizumab Single-Agent Safety Data on Patients with Advanced NSCLC

In the Phase I PCD4989g, in which atezolizumab was used as a single agent in patients with locally advanced or metastatic solid tumors or hematologic malignancies no maximum tolerated (MTD), no dose-limiting toxicities (DLTs) and no clear dose-related trends in the incidence of adverse events was determined. In this study, 520 out of 558 (93%) safety-evaluable patients experienced at least one adverse event, including 376 (67%) patients who experienced one treatment-related adverse event. Commonly reported events (≥10% of all patients) included fatigue, decreased appetite, nausea, pyrexia, constipation and cough. Grade 3-4 adverse events based on the National Cancer Institute Common Terminology Criteria for Adverse Events version 4.0 [NCI CTCAE v4.0] were reported in 239 (43%) patients, of which 66 (12%) were considered related. Grade 3 and 4 adverse events considered related by the investigator included dyspnoea, pneumonitis, increased ALT, increase AST, increased GGT, lymphocyte count decreased, cardiac tamponade, asthenia, autoimmune hepatitis, pneumonia, influenza and hypoxia.

In the randomized Phase II POPLAR Study (GO28753), the frequency of patients who reported any adverse events regardless of attribution was 96% for both arms[30]. A higher number of Grade ≥ 3 adverse events were observed in the docetaxel arm (53% vs 40%), explained mainly by the difference in adverse events due to bone marrow suppression. This difference was more evident in Grade 3-4 treatment-related adverse events (39% vs 11%). The most common atezolizumab Grade 3 adverse events were pneumonia (2%) and increased AST (2%). No atezolizumab-related Grade 4 adverse events were reported. Immune-related adverse events of any grade with atezolizumab were increased AST (4%), increased ALT (4%), pneumonitis (3%), colitis (1%) and hepatitis (1%). Fewer patients discontinued treatment with atezolizumab than with docetaxel (8% vs 22% respectively). There were six (4%) Grade 5 adverse events in atezolizumab were cardiac failure, pneumonia, ulcer haemorrhage, pneumothorax, pulmonary embolism and embolism.

In the randomized Phase III OAK Study, the frequency of patients who reported any adverse event regardless of attribution was similar in both arms: 94% in atezolizumab arm and 96% in docetaxel arm[31]. A higher number of Grade ≥ 3 adverse events were observed in the docetaxel arm (54%) compared with atezolizumab arm (37%). This difference was greater in Grade 3-4 treatment-related adverse events (43% vs 15%). The most common atezolizumab-related adverse events were fatigue (14%), nausea (9%), decreased appetite (9%) and asthenia (8%). Immune-related adverse events reported with atezolizumab included pneumonitis (1%), hepatitis (<1%) and colitis (<1%).

A pooled safety analysis conducted in 843 patients who received atezolizumab in 4 studies (PCD4989g [N = 76]; BIRCH [N = 520]; FIR [N = 105]; POPLAR [N = 142]) (Lukas et al. WCLC 2016). Twenty-seven (3%) of 843 patients had asymptomatic untreated brain metastases or stable previously treated brain metastases at baseline. The incidence of treatment-related neurological AEs was 4 (15%) in patients with baseline brain metastases, including the most common treatment-related AE of headache in 2 (7%) and 27 (3%) patients, respectively. The most common all-cause AEs

in patients with baseline brain metastases were fatigue, nausea, and vomiting (7 [26%] each). No treatment discontinuations occurred due to AEs.

Atezolizumab in Combination with Platinum-Based Chemotherapy Safety Data on patients with Advanced NSCLC

The Study GP28328 is a Phase Ib study of atezolizumab in combination with bevacizumab or cytotoxic chemotherapy in patients with multiple tumor types including NSCLC, triple-negative breast cancer and colorectal cancer. Patients with advanced NSCLC were included in the following arms: Arm C (atezolizumab + carboplatin + paclitaxel), Arm E (atezolizumab + carboplatin + pemetrexed) and Arm E (atezolizumab + carboplatin + nab-paclitaxel). These combinations have been generally well tolerated and no DLTs have been reported during the dose-escalation stage in any study arm. A total of 141 of 144 (98%) patients reported at least one adverse event while receiving study drug. Most of these events were Grade 2 and 3 in severity. The five most commonly adverse events across the study arms (≥10%) included fatigue, nausea, diarrhoea, decreased appetite and pyrexia. The adverse events were consistent with the known safety profile of each agent and no additive effects were observed when atezolizumab was administered with chemotherapy.

Atezolizumab Single-Agent Efficacy Data on Patients with Advanced NSCLC

In the Phase I PCD4989g Study, the efficacy evaluable population included 88 patients with locally advanced or metastatic NSCLC and represented a heavily pre-treated patient population (97% of the patients had received ≥ 2 prior systemic therapies and 77% had received ≥ 4 prior systemic therapies). Overall, responses were observed in 20 out of 88 (23%) patients with NSCLC and included responses in patients with squamous and non-squamous NSCLC (4 in 21 and 16 in 67 patients respectively).

In the POPLAR Study, the primary OS analysis was conducted when 173 deaths had occurred. Most patients had received one prior therapy (65%), had non-squamous histology (66%) and ECOG performance status of 1 (68%). Atezolizumab showed significant improvement in OS compared with docetaxel (12.6 vs 9.7 months; HR = 0.73, 95% CI 0.53-0.99; p = 0.04). OS benefit was associated with tumor PD-L1 overexpression. PFS was similar among both arms (2.7 vs 3 months respectively) and objective responses with atezolizumab were durable, with a median duration of 14.3 months compared with 7.2 months for docetaxel.

In the OAK Study, the primary efficacy analysis population comprised the first 850 patients. Most patients had received one prior therapy (75%), had non-squamous histology (74%) and ECOG performance status of 1 (63%). Atezolizumab showed significant improvement in OS compared with docetaxel (13.8 vs 9.6 months; HR = 0.73, 95% CI 0.62-0.87; p = 0.003). Based on the last data cut off with a minimum follow-up of 26 months, the 2-year OS rate in patients who received atezolizumab was 31% versus 21% in patients treated with docetaxel (Satouchi et al. WCLC 2017). Although the benefit in terms of OS was associated with tumor PD-L1 overexpression, patients with low or undetectable PD-L1 expression (IC0/TC0) also had improved OS with atezolizumab. A post-hoc

subgroup analysis showed that patients with stable previously treated BM also had significant benefit in OS and PFS from atezolizumab (Gadgeel et al. WCLC 2016).

Atezolizumab in Combination with Platinum-Based Chemotherapy Efficacy Data on patients with Advanced NSCLC

In the Study GP28328, patients with advanced NSCLC received atezolizumab q3w in combination with platinum-based chemotherapy: carboplatin + paclitaxel (Arm C), carboplatin + pemetrexed (Arm D) and carboplatin + nab-paclitaxel (Arm E). All patients had histologically or cytologically documented Stage IIIB or IV or recurrent NSCLC and had not received prior chemotherapy for advanced disease. The median age was 65 years and 79% had non-squamous histology. In the first data cut off (February 10th 2015), 41 patients were evaluable for efficacy and the ORR in all three arms was 63%. In the last update of the study, 76 patients were evaluable for efficacy and the ORR according to the three arms was: Arm C (n=25) 46%; Arm D (n=25) 68%; Arm E (n=26) 36% (Liu et al. ASCO 2017). For patients treated with carboplatin + pemetrexed (Arm D), median PFS was 8.4 months (4.7-11) and OS rate at 12 months was 68%.

Refer to the local prescribing information for additional details on nonclinical and clinical studies.

Rationale for Atezolizumab dose

The fixed dose of 1200 mg (equivalent to an average body weight-base dose of 15mg/kg) intravenously was selected on the basis of both nonclinical studies and available clinical data from Study PCD4989g. The target exposure for atezolizumab was projected on the basis of nonclinical tissue distribution data in tumor-bearing mice, target-receptor occupancy in the tumor, the observed atezolizumab interim pharmacokinetics and other factors.

Antitumor activity has been observed across doses from 1 mg/kg to 20 mg/kg. The MTD of atezolizumab was not reached and DLTs have been observed at any dose in Study PCD4989g. Currently available PK and ATA data suggest that the 15-mg/kg atezolizumab q3w regimen (or fixed-dose equivalent) for Phase II and Phase III studies would be sufficient to both maintain Ctrough ≥ 6 µg/mL and further safeguard against both interpatient variability and the potential effect of ATAs that may lead to subtherapeutic levels of atezolizumab relative to the 10-mg/kg atezolizumab q3w regimen.

In this trial, atezolizumab will be given as an intravenous fixed dose of 1200 mg every 3 weeks (q3w) until unacceptable toxicity or disease progression, as assessed by the investigator. Atezolizumab treatment could continue beyond disease progression if the investigator deems the patient to have clinical benefit.

3.4. Background on Bevacizumab

Bevacizumab is a recombinant humanized monoclonal IgG1 antibody that blinds to and inhibits the interaction of VEGF-A to its receptors (Flt-1 and KDR) on the surface of endothelial cells. The

interaction of VEGF with its receptors leads to endothelial cell proliferation and new blood vessel formation in *in vitro* models of angiogenesis.

Neutralising the biological activity of VEGF regresses the vascularisation of tumors, normalises remaining tumor vasculature, and inhibits the formation of new tumor vasculature, thereby inhibiting tumor growth.

Administration of Bevacizumab to xenotransplant models of colon in nude mice caused reduction of microvascular growth and inhibition of metastatic disease progression[32,33].

Bevacizumab is indicated for the first line treatment of unresectable, locally advanced, recurrent or metastatic non-squamous non-small cell lung cancer. The recommended dose of bevacizumab is 15mg/Kg by IV infusion once every 3 weeks (21 days).

3.5. Rationale for trial design

Further understanding the factors associated with increased TMB is important for better understanding this key driver of cancer progression and for understanding the molecular mechanisms which lead to TMB.

FoundationOne CDx™ (F1CDx) is performed exclusively as a laboratory service using DNA extracted from formalin-fixed, paraffin-embedded (FFPE) tumor samples. The assay employs a single DNA extraction method from routine FFPE biopsy or surgical resection specimens, 50-1000 ng of which will undergo whole-genome shotgun library construction and hybridization-based capture of all coding exons from 309 cancer-related genes, one promoter region, one non-coding (ncRNA), and select intronic regions from 34 commonly rearranged genes, 21 of which also include the coding exons. In total, the assay detects alterations in a total of 324 genes. Using the Illumina® HiSeq 4000 platform, hybrid capture–selected libraries are sequenced to high uniform depth (targeting >500X median coverage with >99% of exons at coverage >100X). Sequence data is then processed using a customized analysis pipeline designed to detect all classes of genomic alterations, including base substitutions, indels, copy number alterations (amplifications and homozygous gene deletions), and selected genomic rearrangements (e.g., gene fusions). Additionally, genomic signatures including microsatellite instability (MSI) and tumor mutational burden (TMB) are reported.

FoundationOne®Liquid, its next-generation liquid biopsy test for solid tumors. Using a blood sample, FoundationOne Liquid analyses 70 genes known to drive cancer growth, and reports the genomic biomarker for microsatellite instability (MSI),1 to help inform the use of checkpoint inhibitor immunotherapies and multiple targeted therapies, as well as clinical trials for patients with advanced cancer.

FoundationOne Liquid is a hybrid capture-based, next-generation sequencing in vitro diagnostic device for the detection of substitutions, insertion and deletion alterations (indels), copy number alterations (CNAs) and select gene rearrangements using circulating cell-free DNA (cfDNA) isolated from plasma derived from peripheral whole blood. The FoundationOne Liquid test expands upon the previous version of the Company's liquid biopsy test, FoundationACT®, which has been analytically validated across the four main classes of genomic alterations. Evaluation of the platform using

multiple validation methods across a broad range of tumor types demonstrated high sensitivity2 and positive predictive value3, even at the low allele frequencies often observed in clinical samples

Based on the above synergistic effects, Atezolizumab plus Bevacizumab might provide significant TMB selected NSCLC patients. For this reason, the Fundación GECP finds of great interest the following protocol: A phase 2 study evaluating the efficacy of Atezolizumab in combination with Bevacizumab as first line therapy for advanced or metastatic high-intermediate TMB (TMB≥10 mutations/MB analyzed in tumor or TMB≥16 analyzed in blood) non-squamous NSCLC patients.

At the European Congress of Medical Oncology (ESMO) of this year 2020, the results of the WJOG @Be[38] -A phase II study of Atezolizumab with Bevacizumab for Non-Squamous Non-small-cell lung cancer with high PD-L1 expression by Seto et al. In this study, in a population with PD-L1 positive of more than 50%, not TMB, but with the same treatment as the TELMA study, they found an ORR of 64.1% (95% CI, 47.18-78.80), with a median PFS of 15.9 months (95% CI, 5.65-15.93), being the PFS at one year of 54.9% (95% CI, 35.65-70.60) and an overall survival at one year of 70.6% (95% CI, 50.5-83.4) and a median duration of response 10.4 months.

There are few studies on this population and no others with this treatment combination. From the date of the design of the TELMA study, we have known data from the study of the Ipilimumab-Nivolumab combination for patients with TMB> 10mut / megabase in which they found a PFS at one year of 43% compared to 13% for the control arm.

The same study in a subsequent analysis and in that population PDL1> 50% obtained a PFS at one year of 41% for the combination vs 15% for the control arm with chemotherapy.

At this point, it was considered that primary objective must be modified to 12 months PFS because current data on the efficacy of immunotherapy in populations with a high probability of response, either by identification by TMB or by PDL1 expression, inform us of a greater benefit than expected at the time of design. On the other hand, the selection of patients based on the latest generation NGS technology and the identification of concomitant mutations, makes recruitment very slow and difficult to achieve in adequate time.

3.6. Rationale for additional pharmacogenomic exploratory research (stool and blood samples)

An interesting way to assess the status of the antitumor immune response is the deep sequencing of the TCR and BCR locus. During T cell maturation, their TCR locus suffer somatic rearrangements allowing T cells to recognize different antigens including tumor antigens. Cha et al., showed on melanoma and prostate patients treated with ipilimumab that massive sequencing on TCR locus from PBMCs allow them to correlate T cell repertoire with response to immunotherapy treatment [a]. Later on, Akyüz et al., on another study with 18 patients treated with anti-PD1 found a clear pattern of diversification of T cell clones correlated with the control of the disease [b]. Recently, on 9 patients with NSCLC, Forde et al. have described that the number of T cells clones in tumor or in peripheral

blood increased after anti PD-1 treatment [c]. Importantly, Casarrubios et. al. have shown that the baseline tissue TCR repertoire evenness and top 1% clonal space are associated to pathological complete responses after chemo-immunotherapy (d).

In addition to T cells, B cells play a key role in the antitumor immune response accounting for 20% of TILs in NSCLC and participating in both antigenic presentation and the production of specific antitumor antibodies (e). Recently, several authors have addressed the study of tertiary lymphoid structures (TLS), associating their presence with antitumor immunity and increased overall survival in patients with lung cancer and melanoma (f. Although detailed study is needed to elucidate their role in this context, it has been suggested that B cells present in tertiary structures of solid tumors suffer somatic hypermutation and proliferation in germinal centers acting as antigen presenting cells that favor the expansion of memory T cells, contributing positively to the antitumor response (g.

Therefore, the massive sequencing of the TCR and BCR locus is positioned as an interesting technique for the evaluation of the antitumor immune response.

Besides the characterization of TCR and BCR repertoires, it is interesting to immunophenotype and study the peripheral blood mononuclear cells to determine the levels and activation of distinct regulatory, memory, cytotoxic and helper subpopulations. The impact of the anti-PD-L1 + anti-VEGF combination on PBMCs immunophenotype and its capacity as a source of predictive biomarkers is currently unknown. However, different groups have demonstrated the value of lymphocyte populations in the treatment with immunotherapy in advanced stages (h).

Human gut microbes outnumber host eukariotic cells by 10-fold. These a priori commensal cells modify human health through several mechanisms, being the alteration of systemic immune responses one of the most relevant. Recently, some studies have shown that gut microbiota could mediate diverse responses to immunotherapy and might serve as a potential predictive biomarker. In patients with melanoma receiving anti–PD-1 therapy, significant difference in the diversity and composition of gut microbiome was observed between the responders and nonresponders to the therapy [i]. The relationship between fecal microbiome and response to ICI therapy has been observed also for NSCLC in different cohorts [j] [k].

4. Objectives and endpoints

4.1. Primary objective and endpoint

- To evaluate the efficacy of Atezolizumab in combination with Bevacizumab as measured by Progression Free Survival (PFS) at 12 months according to Response Evaluation Criteria in Solid Tumors (RECIST) Version 1.1.

  PFS after enrollment is defined as the time from enrollment to the first occurrence of disease progression or death from any cause, whichever occurs first.

4.2. Secondary objectives and endpoint

- To evaluate the Overall response rate (ORR) of Atezolizumab in combination with Bevacizumab as measured by investigator-assessed overall response rate (ORR) according to RECIST v1.1.

  ORR defined as percentage of patients reaching complete or partial response.

- To evaluate the efficacy of Atezolizumab in combination with Bevacizumab as measured by investigator-assessed duration of response (DOR) according to RECIST v1.1.

  DOR defined as the time from the first occurrence of a documented objective response to disease progression or death from any cause, whichever occurs first.

- To evaluate the efficacy of Atezolizumab in combination with Bevacizumab as measured by investigator-assessed time to response (TTR) according to RECIST v1.1.

  TTR defined as the time from the start of the treatment to the first objective tumor response observed for patients who achieved CR or PR.

- To evaluate the efficacy of Atezolizumab in combination with Bevacizumab as measured by investigator-assessed ORR, PFS and DOR according to immune-related response criteria; irRC.

- To evaluate the efficacy of Atezolizumab in combination with Bevacizumab as measured by investigator ORR, PFS and DOR according to immune-related response criteria (irRC) in patients with TMB ≥16.

- To evaluate the PFS rate at 1 and 2 years of Atezolizumab in combination with Bevacizumab.

- To evaluate the Overall Survival (OS) rate at 1 and 2 years of Atezolizumab in combination with Bevacizumab.

  OS defined as the length of time from either the date of diagnosis or the start of the treatment that patients diagnosed with the disease are still alive.

- To evaluate ORR and PFS according PD-L1 expression of Atezolizumab in combination with Bevacizumab.

- To evaluate the safety and tolerability of Atezolizumab in combination with Bevacizumab.

  Occurrence and severity of adverse events, with severity determined by NCI CTCAE v5.0 criteria.

- To evaluate patient-reported outcomes (PROs) of lung cancer symptoms, patient functioning, and health-related quality of life (HRQoL) as measured by the European Organisation for Research and treatment of Cancer (EORTC) Quality-of-life Questionnaire

Core 30 (QLQ C30) and its Lung Cancer Module (QLQ LC13) of Atezolizumab in combination with Bevacizumab.

Change from baseline in quality of life, as assessed through use of the EORTC C30 and LC13 at baseline, at cycle 3, every 3 cycles until progression and at progression.

4.3. Exploratory objectives

Foundation one:

- To determine the tumor mutational burden (TMB) and determine its association with all 4 types of genomic alteration (Indels, mutation, CNV, rearrangement) in 324 tumor related genes, MSI and determine by FoundationOne CDx™ (F1CDx), and other biomarkers such as tumor PD-L1 expression.
- To investigate genomics alteration change measure in blood after progression measured with FoundationOne®Liquid, in respect with genomics alteration measure by FoundationOne CDx™ (F1CDx) in tissue including genomics signatures (MSI and TMB) at progression.
- To evaluate the clinical utility of the TMB results/reports in patients with TMB<10, mutations/MB describing druggable alterations or driver mutation that influence treatment selection.
- To evaluate overall survival (OS) and ORR of atezolizumab in combination with bevacizumab based on the TMB analysis in blood and in tumor)

Additional pharmacogenomic exploratory analysis:

- To evaluate the basal peripheral blood TCR and BCR repertoire and their changes during treatment, as well as, their correlation with clinical variables associated with treatment efficacy (ORR, PFS, OS, DOR).
- To study the levels of peripheral blood immune cells and soluble factors from plasma samples considering their changes during treatment, as well as, their correlation with clinical variables associated with treatment efficacy (ORR, PFS, OS, DOR).
- To determine the pre-treatment fecal microbiome of all patients included in the study and its predictive value in terms of ORR, PFS, OS and DOR.

5. Trial design, duration and termination

This is an open-label, non-randomized, phase II, multi-centre clinical trial. Chemotherapy-naïve patients high-intermediate TMB (TMB≥10 mutations/MB analyzed in tumor or TMB≥16 mutations/MB analyzed in blood) and with locally advanced or metastatic non-squamous non-small cell lung cancer patients will be selected.

Patients enrolled in the study will receive Atezolizumab 1200mg + Bevacizumab 15mg/Kg every 21 days (3 weeks ±3days).

Atezolizumab may continue beyond disease progression per RECIST v1.1 until loss of clinical benefit, unacceptable toxicity, patient or physician decision to discontinue, or death. If the principal investigator would like to continue beyond progression a permission from the trial chair/sponsor have to be granted. For all patients, tumor response data collection will continue until disease progression, even if the patient stops study treatment prior to disease progression.

Bevacizumab will be administered until progression disease, unacceptable toxicity, patient or physician decision to discontinue or death.

Patient accrual is expected to be completed within 1.5 years excluding a run-in-period of 4-6 months. Treatment and follow-up are expected to extend the study duration to a total of 4.5 years. Patients will be followed 1.5 years after the end of treatment independently of the cause of end of treatment. The study will end once survival follow-up has concluded.

The trial will end with the preparation of the final report, scheduled for 5.5 years after the inclusion of the first patient approximately.

Rationale for Atezolizumab treatment Beyond progression (TBP)

Cancer immunotherapy in second- or third-line setting of treatment can have a positive impact on OS that exceeds response rate or PFS effects, termed post progression prolongation of survival (PPPS). This effect can also result from unconventional response due to tumor immune infiltration or delayed response, reducing reliability of RECIST v1.1 (RECIST) progression as an indicator of treatment failure. A posthoc analysis evaluated clinical benefit from TBP, defined by post PD tumor regression, OS and safety (Gandara et al. ASCO 2017). Among 332 patients who received atezolizumab and experienced progression, 51% (n = 168) continued atezolizumab TBP; 7% (12/168) achieved subsequent response in target lesions and 49% (83/168) had stable target. Median OS was 12.7 months (95% CI 9.3 - 14.9) post PD for pts on atezolizumab TBP. TBP with atezolizumab was not associated with increased safety risk.

In this clinical trial, patients will be fully informed of the risk of continuing study treatment in spite of apparent radiographic progression when they maintain clinical benefit, and investigators should make a careful assessment of the potential benefit of doing so, considering radiographic data, biopsy results and the clinical status of the patient.

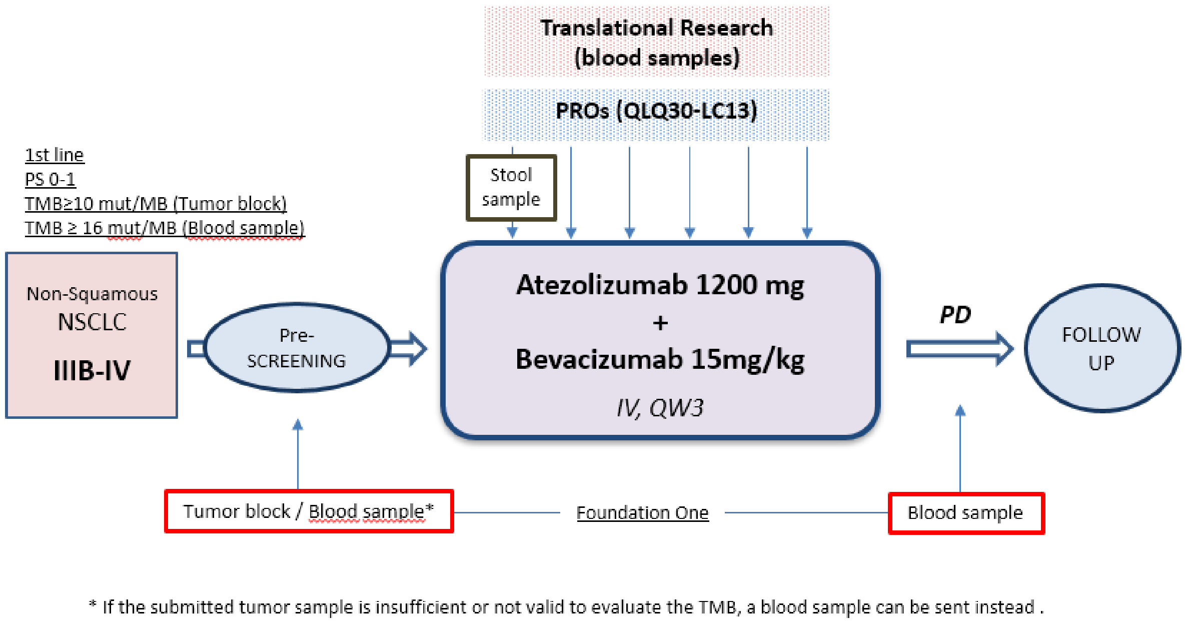

6. Patient selection

Written Informed Consent (IC) must be signed and dated by the patient and the investigator prior to any trial-related intervention for trial treatment and biomaterial submission to the central laboratory.

Patients should only be selected and consented for enrollment if they fulfil the following criteria:

6.1. Inclusion criteria

1. Male or female, aged ≥ 18 years old.
2. ECOG performance status of 0 or 1.
3. Histologically or cytologically confirmed, Stage IIIB - IV non-squamous NSCLC according to 8th version of the International Association for the Study of Lung Cancer Staging Manual in Thoracic Oncology.
4. No prior treatment for Stage IIIB - IV non-squamous NSCLC.
5. Patients who have received prior neo-adjuvant, adjuvant chemotherapy, radiotherapy, or chemo-radiotherapy with curative intent for non-metastatic disease must have experienced a treatment-free interval of at least 6 months from enrollment since the last chemotherapy, radiotherapy, or chemo-radiotherapy.

6. Patients with a treated asymptomatic CNS metastasis are eligible, provided they meet all of the following criteria:
    a. Only supratentorial and cerebellar metastases allowed (i.e. no metastases to midbrain, pons, medulla or spinal cord).
    b. No ongoing requirement for corticosteroids as therapy for CNS disease.
    c. No stereotactic radiation within 7 days or whole-brain radiation within 14 days prior to enrollment.
    d. No evidence of interim progression between the completion of CNS-directed therapy and the screening radiographic study.

   Patients with new asymptomatic CNS metastases detected at the screening scan must receive radiation therapy and/or surgery for CNS metastases. Following treatment, these patients may then be eligible without the need for an additional brain scan prior to inclusion, if all other criteria are met.
7. Patients with high-intermediate Tumor Mutational Burden analyzed by Foundation Medicine (≥10 mutations/ MB) performed by a Foundation Medicine laboratory on previously obtained archival tumor tissue or tissue obtained from a biopsy at pre-screening (sample must fulfil minimal sample requirements of 20% tumor cellularity and a minimum surface of 25mm^2). TMB≥16 mutations/ MB is required if the analysis is performed in blood sample.
8. Measurable disease, as defined by RECIST v1.1.

   Previously irradiated lesions can only be considered as measurable disease if disease progression has been unequivocally documented at that site since radiation and the previously irradiated lesion is not the only site of disease.
9. Adequate hematologic and organ function defined by the following laboratory. results obtained within 14 days prior to enrollment:
    - Neutrophils ≥ 1500 cells/µL without granulocyte colony-stimulating factor support.
    - Lymphocyte count ≥ 500/µL.
    - Platelet count ≥ 100,000/µL without transfusion.
    - Haemoglobin ≥ 9.0 g/dL. Patients may be transfused to meet this criterion.
    - INR or aPTT ≤ 1.5 × upper limit of normal (ULN). This applies only to patients who are not receiving therapeutic anticoagulation; patients receiving therapeutic anticoagulation should be on a stable dose.
    - AST, ALT, and alkaline phosphatase ≤ 2.5 × ULN, with the following exceptions:

      Patients with documented liver metastases: AST and/or ALT ≤ 5 × ULN.

      Patients with documented liver or bone metastases: alkaline phosphatase ≤ 5 × ULN.
    - Serum bilirubin ≤ 1.25 × ULN. Patients with known Gilbert disease who have serum bilirubin level ≤ 3 × ULN may be enrolled.
    - Serum creatinine ≤ 1.5 × ULN or creatinine clearance of ≥45ml/min (based on the Cockcroft Gault formula).

10. All patients are notified of the investigational nature of this study and signed a written

informed consent in accordance with institutional and national guidelines, including the Declaration of Helsinki prior to any trial-related intervention.

11. For female patients of childbearing potential, agreement (by patient and/or partner) to use a highly effective form(s) of contraception that results in a low failure rate (< 1% per year) when used consistently and correctly, and to continue its use for 5 months after the last dose of Atezolizumab and/or 6 months after the last dose of Bevacizumab, whichever is later. Such methods include: combined (oestrogen and progestogen containing) hormonal contraception, progestogen-only hormonal contraception associated with inhibition of ovulation together with another additional barrier method always containing a spermicide, intrauterine device (IUD): intrauterine hormone-releasing system (IUS), bilateral tubal occlusion, vasectomized partner (on the understanding that this is the only one partner during the whole study duration), and sexual abstinence.
12. For male patients with female partners of childbearing potential, agreement (by patient and/or partner) to use a highly effective form(s) of contraception that results in a low failure rate [< 1% per year] when used consistently and correctly, and to continue its use for 6 months after the last dose of Bevacizumab. Male patients should not donate sperm during this study and for at least 6 months after the last dose of Bevacizumab.
13. Oral contraception should always be combined with an additional contraceptive method because of a potential interaction with the study drugs. The same rules are valid for male patients involved in this clinical study if they have a partner of childbirth potential. Male patients must always use a condom.
14. Women who are not postmenopausal (≥ 12 months of non−therapy-induced amenorrhea) or surgically sterile must have a negative serum pregnancy test result within 8 days prior to initiation of study drug.

6.2. Exclusion criteria

Cancer-Specific Exclusions:

1. Patients with a sensitizing mutation or an amplification in the epidermal growth factor receptor (EGFR) gene.
2. Patients with an anaplastic lymphoma kinase (ALK) fusion oncogene.
3. Patients with an STK-11 Ligand alteration.
4. Patients with MDM2 amplification.
5. Patients with ROS1 translocations.
6. Active or untreated CNS metastases as determined by CT or magnetic resonance imaging (MRI) evaluation during screening and prior radiographic assessments.
7. Spinal cord compression not definitively treated with surgery and/or radiation or previously diagnosed and treated spinal cord compression without evidence that disease has been clinically stable for > 2 weeks prior to enrollment.

8. Leptomeningeal disease.
9. Uncontrolled tumor-related pain.
   - Patients requiring pain medication must be on a stable regimen at study entry.
   - Symptomatic lesions amenable to palliative radiotherapy (e.g., bone metastases or metastases causing nerve impingement) should be treated prior to initiation of study drug.
   - Patients should be recovered from the effects of radiation. There is no required minimum recovery period.
   - Asymptomatic metastatic lesions whose further growth would likely cause functional deficits or intractable pain (e.g., epidural metastasis that is not currently associated with spinal cord compression) should be considered for locoregional therapy, if appropriate, prior to initiation of study drug.
10. Uncontrolled pleural effusion, pericardial effusion, or ascites requiring recurrent drainage procedures (once monthly or more frequently).
    - Patients with indwelling catheters (e.g., PleurX®) are allowed.
11. Uncontrolled or symptomatic hypercalcemia (> 1.5 mmol/L ionized calcium or Ca > 12 mg/dL or corrected serum calcium > ULN).
12. Malignancies other than NSCLC within 5 years prior to enrollment, with the exception of those with a negligible risk of metastasis or death (e.g., expected 5-year OS > 90%) treated with expected curative outcome (such as adequately treated carcinoma in situ of the cervix, basal or squamous-cell skin cancer, localized prostate cancer treated with radiotherapy or surgically with curative intent, ductal carcinoma in situ treated surgically with curative intent).

General Medical Exclusions:

13. Women who are pregnant, lactating, or intending to become pregnant during the study.
14. History of severe allergic, anaphylactic, or other hypersensitivity reactions to chimeric or humanized antibodies or fusion proteins.
15. Known hypersensitivity or allergy to biopharmaceuticals produced in Chinese hamster ovary cells or any component of the Atezolizumab formulation.
16. History of autoimmune disease, including but not limited to myasthenia gravis, myositis, autoimmune hepatitis, systemic lupus erythematosus, rheumatoid arthritis, inflammatory bowel disease, vascular thrombosis associated with antiphospholipid syndrome, Wegener's granulomatosis, Sjögren's syndrome, Guillain-Barré syndrome, multiple sclerosis, vasculitis, or glomerulonephritis.
    - Patients with a history of autoimmune-related hypothyroidism on a stable dose of thyroid replacement hormone are eligible for this study.
    - Patients with controlled Type 1 diabetes mellitus on a stable dose of insulin regimen are eligible for this study.

Patients with eczema, psoriasis, lichen simplex chronicus, or vitiligo with dermatologic manifestations only (e.g., patients with psoriatic arthritis would be excluded) are permitted provided that they meet the following conditions:

- Rash must cover less than 10% of body surface area (BSA).
- Disease is well controlled at baseline and only requiring low-potency topical steroids.
- No acute exacerbations of underlying condition within the previous 12 months (not requiring PUVA [psoralen plus ultraviolet A radiation], methotrexate, retinoids, biologic agents, oral calcineurin inhibitors, high-potency or oral steroids).

17. History of idiopathic pulmonary fibrosis, organizing pneumonia (e.g., bronchiolitis obliterans), drug-induced pneumonitis, idiopathic pneumonitis, or evidence of active pneumonitis on screening chest CT scan.

    History of radiation pneumonitis in the radiation field (fibrosis) is permitted.
18. Positive test for HIV. All patients will be tested for HIV prior to inclusion into the study; patients who test positive for HIV will be excluded from the clinical study.
19. Patients with active hepatitis B (chronic or acute; defined as having a positive hepatitis B surface antigen [HBsAg] test at screening) or hepatitis C.

    Patients with past hepatitis B virus (HBV) infection or resolved HBV infection (defined as the presence of hepatitis B core antibody [HBcAb] and absence of HBsAg) are eligible only if they are negative for HBV DNA (vaccinated patients are excluded).

    Patients positive for hepatitis C virus (HCV) antibody are eligible only if PCR is negative for HCV RNA.
20. Active tuberculosis.
21. Severe infections within 4 weeks prior to be included in the study, including but not limited to hospitalization for complications of infection, bacteraemia, or severe pneumonia.
22. Received therapeutic oral or IV antibiotics within 2 weeks prior to be included in the study.

    Patients receiving prophylactic antibiotics (e.g., for prevention of a urinary tract infection or to prevent chronic obstructive pulmonary disease exacerbation) are eligible.
23. Significant cardiovascular disease, such as New York Heart Association cardiac disease (Class II or greater), myocardial infarction, or cerebrovascular accident within 3 months prior to inclusion, unstable arrhythmias, or unstable angina.

    Patients with known coronary artery disease, congestive heart failure not meeting the above criteria, or left ventricular ejection fraction < 50% must be on a stable medical regimen that is optimized in the opinion of the treating physician, in consultation with a cardiologist if appropriate.
24. Major surgical procedure other than for diagnosis within 28 days prior to inclusion or anticipation of need for a major surgical procedure during the course of the study.
25. Prior allogeneic bone marrow transplantation or solid organ transplant.

26. Administration of a live, attenuated vaccine within 4 weeks before inclusion or anticipation that such a live attenuated vaccine will be required during the study.
27. Any other diseases, metabolic dysfunction, physical examination finding, or clinical laboratory finding giving reasonable suspicion of a disease or condition that contraindicates the use of an investigational drug or that may affect the interpretation of the results or renders the patient at high risk from treatment complications.
28. Patients with illnesses or conditions that interfere with their capacity to understand follow and/or comply with study procedures.

Exclusion Criteria Related to Medications:

29. Any approved anti-cancer therapy, including hormonal therapy, within 3 weeks prior to initiation of study treatment.
30. Treatment with any other investigational agent with therapeutic intent within 28 days prior to initiation of study treatment.
31. Treatment with systemic immunosuppressive medications (including but not limited to corticosteroids, cyclophosphamide, azathioprine, methotrexate, thalidomide, and anti−tumor necrosis factor [anti-TNF] agents) within 2 weeks prior to inclusion.

    Patients who have received acute, low-dose (≤ 10 mg oral prednisone or equivalent), systemic immunosuppressant medications may be enrolled in the study.

    The use of corticosteroids (≤ 10 mg oral prednisone or equivalent) for chronic obstructive pulmonary disease, mineralocorticoids (e.g., fludrocortisone) for patients with orthostatic hypotension, and low-dose supplemental corticosteroids for adrenocortical insufficiency is allowed.

Exclusions Related to Bevacizumab:

32. Inadequately controlled hypertension (defined as systolic blood pressure > 150 mmHg and/or diastolic blood pressure > 100 mmHg).

    Anti-hypertensive therapy to achieve these parameters is allowable.
33. Prior history of hypertensive crisis or hypertensive encephalopathy.
34. Significant vascular disease (e.g., aortic aneurysm requiring surgical repair or recent peripheral arterial thrombosis) within 6 months prior to inclusion.
35. History of haemoptysis (≥ one-half teaspoon of bright red blood per episode) within 1 month prior to inclusion.
36. Evidence of bleeding diathesis or coagulopathy (in the absence of therapeutic anticoagulation).
37. Current or recent (within 10 days of inclusion) use of aspirin (> 325 mg/day) or treatment with dipyramidole, ticlopidine, clopidogrel, and cilostazol.
38. Current use of full-dose oral or parenteral anticoagulants or thrombolytic agents for therapeutic purposes that has not been stable for > 2 weeks prior to inclusion.

The use of full-dose oral or parenteral anticoagulants is permitted as long as the INR or aPTT is within therapeutic limits (according to the medical standard of the enrolling institution) and the patient has been on a stable dose of anticoagulants for at least 2 weeks prior to inclusion.

Prophylactic anticoagulation for the patency of venous access devices is allowed, provided the activity of the agent results in an INR < 1.5 × ULN and aPTT is within normal limits within 14 days prior to inclusion.

Prophylactic use of low-molecular-weight heparin (i.e., enoxaparin 40 mg/day) is permitted.

39. Core biopsy or other minor surgical procedure, excluding placement of a vascular access device, within 7 days prior to the first dose of Bevacizumab.
40. History of abdominal or tracheoesophageal fistula or gastrointestinal perforation within 6 months prior to inclusion.
41. Clinical signs of gastrointestinal obstruction or requirement for routine parenteral hydration, parenteral nutrition, or tube feeding.
42. Evidence of abdominal free air not explained by paracentesis or recent surgical procedure.
43. Serious, non-healing wound, active ulcer, or untreated bone fracture.
44. Proteinuria, as demonstrated by urine dipstick or > 1.0 g of protein in a 24-hour urine collection.

    All patients with ≥ 2+ protein on dipstick urinalysis at baseline must undergo a 24-hour urine collection and must demonstrate ≤ 1 g of protein in 24 hours.
45. Known sensitivity to any component of Bevacizumab.
46. Clear tumor infiltration into the thoracic great vessels is seen on imaging.
47. Clear cavitation of pulmonary lesions is seen on imaging.

7. Patient pre-screening and enrollment

The patients will be eligible for the study after the tumor mutation burden (TMB) determination. This analysis will be done at the pre-screening phase of the study. The patient pre-screening form (appendix 8) has to be sent to the Fundación GECP headquarters scanned by email to the clinical monitor or by fax (+34 93 419 17 68). A pre-screening number will be assigned and returned to the participating hospital by fax or email. After the TMB determination if the patient has a TMB≥10 mut/MB analyzed in tumor or TMB≥16 analyzed in blood and fulfil all the eligibility criteria, the patient will be enrolled in the study.

Note that written informed consent has to be obtained from the patient prior to any trial-specific intervention. The patient has to sign a specific pre-screening informed consent to send the tumor block for pre-screening (TMB analysis). If the submitted tumor sample is insufficient or not valid and a biopsy is not feasible to be performed to evaluate the TMB, a blood sample can be sent instead.

A permission from the trial chair/sponsor must be requested in case no tumor sample is available for a possible patient and blood sample want to be sent to analyze the TMB.

If the patient has a TMB ≥10 analyzed in tumor TMB≥16 analyzed in blood then the patient will sign the Informed consent of the study.

This trial will use a web-based enrollment system (eCRF-Electronic Case Report Form). Each participating centre will access the system directly to enrol the patients.

Patient eligibility needs to be checked before enrollment of the patient in the Fundación GECP web-based system (eCRF). The dates of the patient signature of the Informed Consent of the study and the specific informed consent to pathology material submission for pre-screening are required to be filled in the eligibility checklist of the eCRF.

The treatment has to start preferably the date of enrollment or within 1-5 days from the date of enrollment.

8. Trial procedures

8.1. Overview of treatment sequence

8.1.1. Pre-screening phase

During pre-screening phase, the following procedures must be done:

1. Sign the specific pre-screening informed consent.

2. Sent patient pre-screening form to Fundación GECP to obtain pre-screening number for the patient.

3. Foundation one: the tumor sample must be shipped to central laboratory. Sample must fulfil minimal sample requirements of 20% tumor cellularity and a minimum surface of $25mm^2$.

4. If the submitted tumor sample is insufficient or not valid and a biopsy is not feasible to be performed to evaluate the TMB, a blood sample can be sent instead. A permission from the trial chair/sponsor must be requested in case no tumor sample is available for a possible patient and blood sample want to be sent to analyze the TMB.

Once the central lab has the TMB analysis results, the Fundación GECP staff will send back the patient pre-screening form with the outcome for the enrollment.

8.1.2. Screening/Baseline visit:

Once a patient pre-screening form is received with a positive enrollment outcome (TMB ≥10 mut/MB analyzed in tumor or TMB≥16 analyzed in blood), the patient must sign the informed consent of the study, the eligibility criteria must be reviewed, and the following procedures must be performed 28 days before enrollment:

1. Demographic data including age and gender and medical relevant history, including comorbidities and allergies, surgeries, cancer history (including prior cancer therapies and procedures), reproductive status and smoking history.

2. Complete physical examination including: Height, weight, ECOG, Temperature and blood pressure.

3. Laboratory testing must be performed within 14 days before the enrollment and includes:
   - **Hematology and coagulation**: WBC count, neutrophils, haemoglobin, platelet count, PT, INR and APTT.
   - **Biochemistry**: Ca, Mg, Na, K, Glucose, ALT, AST, LDH, total bilirubin, alkaline phosphatase, creatinine and clearance of creatinine calculated by Cockcroft-Gault formula, total protein, albumin, amylase, lipase.
   - **Thyroid function**: thyroid-stimulating hormone (TSH). Total or free T3 and total or free T4 must be analyzed if TSH result is out of ULN or LLN.
   - **HIV / Hepatitis B/C test**: The following local laboratory assessments should be done <u>within 28 days prior to first dose</u>: Hepatitis B and C testing (HBV sAg, HBV Ab and HCV Ab) and HIV test. In patients with positive hepatitis B/C results, HBV DNA/HCV RNA must be performed (Hepatitis B vaccinated patients are excluded).
   - **Pregnancy Tests**: A serum or urine pregnancy testing is required within <u>3 days prior to study enrollment</u>.
   - **Urine test**: A urine testing (dipstick is allowed) is required for proteinuria within <u>3 days prior to study enrollment</u>.

4. Electrocardiograms: A 12-lead ECG is required at screening and as clinically indicated. ECGs should be obtained on the same machine whenever possible. Lead placement should be as consistent as possible. ECG recordings should be performed after the patient has been resting in a supine position for at least 10 minutes. For safety monitoring purposes, the investigator must review, sign, and date all ECG tracings. Paper copies of ECG tracings will be kept as part of the patient's permanent study file at the site. Any morphologic waveform changes or other ECG abnormalities must be documented on the eCRF.

5. Baseline symptoms within the sign of study's Informed Consent will be recorded.

6. Medications (e.g. prescription drugs, over-the-counter drugs, vaccines, herbal or homeopathic remedies, nutritional supplements) from 7 days prior to initiation of study treatment (day 1 cycle 1) will be recorded.

7. Tumor assessment must be done within 28 days before enrollment (+ 12 days):

   - **Chest and abdomen CT SCAN** with oral/IV contrast (unless contraindicated) to determine measurable disease according to RECIST v1.1 (at least one lesion outside of irradiated areas that can be measured in at least one dimension as ≥ 10 mm, or ≥ 15 mm in case of lymph nodes).
   - **An MRI or CT brain** with oral/IV contrast (unless contraindicated) must be done at baseline within 28 days before enrollment (+ 12 days).
   - A full-body PET-TAC, pelvis or the neck CT scan and bone scans should be performed if clinically indicated.

8. Patient Reported Outcomes (PRO): QLQ30-LC13 (see appendix 5).
9. Pharmacogenomic exploratory analysis: blood samples will be drawn before day 1 of cycle 1 treatment
10. Stool sample will be collected

8.1.3. Bevacizumab and Atezolizumab cycles

Day 1 of cycle 1 treatment will start within 1-5 days from enrollment. Cycles will be administered every 3 weeks (±3 days) until progression or other reason to discontinue. If a patient must stop one of the two drugs due to toxicity it is allowed to continue treatment with the other drug as long as patient does not present progression disease. If progression disease is shown is only permitted continue treatment beyond progression with atezolizumab.

On Day 1 of each cycle, all eligible patients will receive drug infusions in the following order:

1. *Atezolizumab: 1200mg, IV infusion*
2. *Bevacizumab: 15 mg/kg, IV infusion*

Patients should receive treatments according to the local standard of care and manufacturer's instruction. A suggested infusion times for treatment administration:

	Atezolizumab	Bevacizumab
Infusion rate	Over 60 (±15) min (for the first	Over 90 (±15) min (for the first infusion);

	infusion);<br>30 (± 10) min for subsequent<br>infusions if tolerated	shortening to<br>60 (± 10) then<br>30 (± 10) min for subsequent infusions if tolerated

The following procedures must be performed on day 1 of each cycle (±3 days):

1. Physical examination, including: weight, ECOG, Temperature and blood pressure.
2. Blood pressure must be taken always before the drug administration. Blood pressure should be routinely measured before every infusion after a resting period of 10 min. If baseline record is performed more than 3 days before first study dose, they must be repeated on day 1 cycle 1.
3. Laboratory testing prior to each dose administration within 3 days prior to day 1 administration of each cycle. If baseline testing is performed more than 3 days before first study dose, they must be repeated on day 1 cycle 1:
    - **Hematology and coagulation**: WBC count, neutrophils, haemoglobin, platelet count, PT, INR and APTT.
    - **Biochemistry**: Ca, Mg, Na, K, Glucose, ALT, AST, LDH, total bilirubin, alkaline phosphatase, creatinine and clearance of creatinine calculated by Cockcroft-Gault formula, total protein, albumin, amylase, lipase.
    - **Thyroid function**: thyroid-stimulating hormone (TSH). Total or free T3 and total or free T4 must be analyzed if TSH result is out of ULN or LLN. It is required at cycle 2 and then every 2 cycles (more frequently if clinical indicated).
    - **Pregnancy Tests**: A serum or urine pregnancy testing is required at cycle 2 and then every 2 cycles (more frequently if required by local standard).
    - **Urine test**: A urine testing (dipstick is allowed) is required for proteinuria.
4. Adverse events: Changes from baseline symptoms (new or worsened clinically significant ones) should be recorded as adverse events on the Adverse Event form of the eCRF. For laboratory parameters, only grade 2 events will be reported as an AE. All the other events not related to laboratory values will be reported from grade 1.

5. Concomitant medications: Changes from baseline should be recorded on the eCRF.
6. Tumor assessment will be done every 12 weeks (± 7 days) from cycle 1 day 1. CT SCAN must be performed until progression disease or until last dose of Atezolizumab in case of continuing beyond progression. The CT SCAN schedule will be maintained even if a delay in administration occurs.
   - **Chest and abdomen CT SCAN** with oral/IV contrast (unless contraindicated) to determine measurable disease according to RECIST v1.1 (at least one lesion outside of irradiated areas that can be measured in at least one dimension as ≥ 10 mm, or ≥ 15 mm in case of lymph nodes).
   - A full-body PET-TAC, pelvis or the neck CT scan, MRI or CT brain and bone scans should be performed if clinically indicated.
7. PRO (QLQ30-LC13) must be performed always before dose administration, starting on day 1 of cycle 3 and then every 3 cycles. In the event of a delay in administration, the PROs schedule will be also delayed to be performed on day 1 of the specified cycles.
8. Pharmacogenomic exploratory analysis: blood samples will be drawn at:
   - First CT-scan evaluation (cycle 5 if there is no delay in treatment administration)
   - Second CT-scan evaluation (cycle 9 if there is no delay in treatment administration)
   - Fourth CT-scan evaluation (cycle 17 if there is no delay in treatment administration)
   - Sixth CT-scan evaluation (cycle 25 if there is no delay in treatment administration)
   - Progression disease, if it occurs during the bevacizumab + atezolizumab treatment

8.1.4. Atezolizumab cycles (beyond progression):

Atezolizumab may continue beyond disease progression per RECIST v1.1 until loss of clinical benefit, unacceptable toxicity, patient or physician decision to discontinue, or death. If the principal investigator would like to continue beyond progression a permission from the trial chair/sponsor have to be granted.

Cycles will be administered every 3 weeks (±3 days). Patient will receive:

Atezolizumab: 1200mg, IV infusion

The following procedures must be performed on day 1 of each cycle (±3 days):

1. Physical examination, including: weight, ECOG, Temperature and blood pressure (always before the drug administration).
2. Laboratory testing prior to each dose administration within 72 hours prior to day 1 administration of each cycle:

   - **Hematology and coagulation**: WBC count, neutrophils, hemoglobin, platelet count, PT, INR and APTT.
   - **Biochemistry**: Ca, Mg, Na, K, Glucose, ALT, AST, LDH, total bilirubin, alkaline phosphatase, creatinine and clearance of creatinine calculated by Cockcroft-Gault formula, total protein, albumin, amylase, lipase.
   - **Thyroid function**: thyroid-stimulating hormone (TSH). Total or free T3 and total or free T4 must be analyzed if TSH result is out of ULN or LLN. It is required at cycle 2 and then every 2 cycles (more frequently if clinical indicated).
   - **Pregnancy Tests**: A serum or urine pregnancy testing is required at cycle 2 and then every 2 cycles (more frequently if required by local standard).

3. Adverse events: Changes from baseline symptoms (new or worsened clinically significant ones) should be recorded as adverse events on the Adverse Event form of the eCRF. For laboratory parameters, only grade 2 events will be reported as an AE. All the other events not related to laboratory values will be reported from grade 1.
4. Concomitant medications: Changes from baseline should be recorded on the eCRF.
5. Tumor assessment will be done every 12 weeks (± 7 days) from cycle 1 day 1. The CT SCAN schedule will be maintained even if a delay in administration occurs:

   a. **Chest and abdomen CT SCAN** with oral/IV contrast (unless contraindicated) to determine measurable disease according to RECIST v1.1 (at least one lesion outside of irradiated areas that can be measured in at least one dimension as ≥ 10 mm, or ≥ 15 mm in case of lymph nodes).

   b. A full-body PET-TAC, pelvis or the neck CT scan, MRI or CT brain and Bone scans should be performed if clinically indicated.

8.1.5. End of treatment and first follow up visit

End of treatment is defined as the day of discontinuation of the treatment. If the **reason for the end of treatment is progression** the following procedures must be performed between the end of treatment and the first follow up visit:

1. Patient Reported Outcomes (PRO): QLQ30-LC13 (see appendix 5). Even if the progression disease occurs within 9 weeks after of the last completed PRO, the progression PRO must be performed.
2. Blood samples for translational research must be drawn and shipped to central lab.

A first follow up visit must be done 30 days (±3 days) after the last dose of treatment (Bevacizumab+Atezolizumab or Atezolizumab beyond progression) or immediately before the initiation of any other anticancer therapy.

The following procedures must be performed:

1. Physical examination, including: weight, ECOG, Temperature and blood pressure.
2. Laboratory testing:
    - **Hematology and coagulation**: WBC count, neutrophils, hemoglobin, platelet count, PT, INR and APTT.
    - **Biochemistry**: Ca, Mg, Na, K, Glucose, ALT, AST, LDH, total bilirubin, alkaline phosphatase, creatinine and clearance of creatinine calculated by Cockcroft-Gault formula , total protein, albumin, amylase, lipase.
    - **Thyroid function**: thyroid-stimulating hormone (TSH). Total or free T3 and total or free T4 must be analyzed if TSH result is out of ULN or LLN.
    - **Pregnancy Tests**: A serum or urine pregnancy testing is required.
    - **Urine test**: A urine testing (dipstick is allowed) is required for proteinuria.
3. Electrocardiograms: A 12-lead ECG is required at screening and as clinically indicated. ECGs should be obtained on the same machine whenever possible. Lead placement should be as consistent as possible. ECG recordings should be performed after the patient has been resting in a supine position for at least 10 minutes. For safety monitoring purposes, the investigator must review, sign, and date all ECG tracings. Paper copies of ECG tracings will be kept as part of the patient's permanent study file at the site. Any morphologic waveform changes or other ECG abnormalities must be documented on the eCRF.
4. Adverse events: Changes from baseline abnormalities (new or worsened clinically significant ones) should be recorded as adverse events on the Adverse Event eCRF until this visit. For laboratory parameters, only grade 2 events will be reported as an AE. All the other events not related to laboratory values will be reported from

grade 1. SAEs and AESIs must be recorded and followed from the subject written informed consent signature to 30 days from last dose of treatment.

5. Concomitant medications: Changes from baseline should be recorded on the eCRF until this visit (30 days from last dose of treatment).

6. Tumor assessment will be done every 12 weeks (± 7 days) from cycle 1 day 1. In case end of treatment reason is other than disease progression the CT SCAN schedule will be maintained every 12 weeks.:

- **Chest and abdomen CT SCAN** with oral/IV contrast (unless contraindicated) to determine measurable disease according to RECIST v1.1 (at least one lesion outside of irradiated areas that can be measured in at least one dimension as ≥ 10 mm, or ≥ 15 mm in case of lymph nodes).
- A full-body PET-TAC, pelvis or the neck CT scan, MRI or CT brain and Bone scans should be performed if clinically indicated.

It is not mandatory to perform the tumor assessment in first follow up visit if the end of treatment reason is other than progression disease and a tumor assessment is been performed in the previous 12 weeks.

8.1.6. Follow up visits

The subsequent follow up visits can be matched at least with the CT SCAN schedule or more frequently as per local standard.

The following procedures must be performed:

1. Physical examination, including ECOG.
2. Tumor assessment will be done every 12 weeks (± 7 days) from cycle 1 day 1 until progression. In case end of treatment reason is other than disease progression the CT SCAN schedule will be maintained every 12 weeks.

- **Chest and abdomen CT SCAN** with oral/IV contrast (unless contraindicated) to determine measurable disease according to RECIST v1.1 (at least one lesion outside of irradiated areas that can be measured in at least one dimension as ≥ 10 mm, or ≥ 15 mm in case of lymph nodes).
- A full-body PET-TAC, pelvis or the neck CT scan, MRI or CT brain and Bone scans should be performed if clinically indicated.

If the patient withdraws the study due to progression the subsequent follow up visits can be matched at least with the CT SCAN schedule planned as per local standard.

3. At the moment of progression (if applicable):

   - Patient Reported Outcomes (PRO): QLQ30-LC13 (appendix 5). Even if the progression disease occurs within 9 weeks after of the last completed PRO, the progression PRO must be performed.

   - Foundation one: Blood samples for translational research must be drawn and shipped to the central laboratory.

4. Pharmacogenomic exploratory analysis_ blood samples will be drawn at:

   - Progression disease, if it occurs during the follow up phase

8.1.7. Quality of Life (QoL)

The quality of life (QoL) of patients will be assessed using EORTC QLQ-C30[34] and the lung cancer module (QLQ-LC13) questionnaires. The EORTC QLQ-C30[34] is a self-administered cancer specific questionnaire with multi-dimensional scales. It consists of both multi-item scales and single item measures, including five functioning domains, a global quality of life domain, three symptom domains and six single items. For each domain or single item measure a linear transformation will be applied to standardize the raw score to range between 0 and 100.
The QLQ-LC13 lung cancer module[35] includes questions assessing lung cancer-associated symptoms (cough, hemoptysis, dyspnea, and site-specific pain), treatment-related side effects (sore mouth, dysphagia, peripheral neuropathy and alopecia) and pain medication. The validity and reliability have been studied by the EORTC Study Group on Quality of Life. The EORTC QLQ-C30 and module will be scored according to the EORTC QLQ-C30 Scoring Manual and analyzed accordingly.
Changes of the quality of life scores while on treatment versus baseline will be examined using analysis and descriptive statistics. Specific time points will be checked to explore treatment side effect on patients QoL, and the longtime benefit of the study treatment.
Please see Sections 8.1.2, 8.1.3, 8.1.4, 8.1.5 and 8.1.6 to know the QoL tests schedules.

9. Investigational Medicinal Product

Atezolizumab and Bevacizumab are the Investigational Medicinal Products (IMP) used in this trial. Roche will provide the IMPs at no cost for this trial.

Complete details of the trial drug logistics, distribution, packaging, labelling, storage and handling as well as accountability are described in the ***TELMA drug supply manual*** that will be provided in the Pharmacy site file to all the participant sites.

Description of the Investigational Medicinal Products

Atezolizumab (MPDL3280A) is a humanized immunoglobulin (Ig) G1 monoclonal antibody consisting of two heavy chains (448 amino acids) and two light chains (214 amino acids) and is produced in

Chinese hamster ovary cells. Atezolizumab was engineered to eliminate Fc-effector function via a single amino acid substitution on position 298 of the heavy chain, which results in a non-glycosylated antibody that has minimal binding to Fc receptors and prevents Fc-effector function at expected concentrations in humans.

Bevacizumab is a recombinant humanized monoclonal IgG1 antibody that blinds to and inhibits the interaction of VEGF-A to its receptors (Flt-1 and KDR) on the surface of endothelial cells. The interaction of VEGF with its receptors leads to endothelial cell proliferation and new blood vessel formation in *in vitro* models of angiogenesis. Neutralizing the biological activity of VEGF regresses the vascularization of tumors, normalizes remaining tumor vasculature, and inhibits the formation of new tumor vasculature, thereby inhibiting tumor growth.

9.1. Packaging and labelling

Roche will provide the IMP (Atezolizumab and Bevacizumab) at no cost for this study. Clinical supplies will be affixed with a clinical label in accordance with regulatory requirements.

The atezolizumab (MPDL3280A) drug product is provided as a sterile liquid in 20-mL glass vials. The vial is designed to deliver 20 mL (1200 mg) of atezolizumab solution but may contain more than the stated volume to enable delivery of the entire 20 mL volume. For further details on the formulation and handling of atezolizumab, see the Pharmacy Manual and Investigator's Brochure.

The Avastin® (bevacizumab) drug product is provided as a concentrate for solution for infusion (400mg/16ml) per vial. For further details on the formulation and handling of bevacizumab, see the summary of product characteristics (SmPC).

9.2. Receipt and storage of the IMP

Accurate records of the IMP received at, dispensed from, returned to, and disposed of by the study site should be recorded on the Drug Accountability Log. In the event that the IMPs are destroyed a certification of destruction form should be generated and retained in the Pharmacy Site File.

The IMPs must be stored in a secure area according to local regulations. The investigator must ensure that the IMPs are stored refrigerated at 2-8°C, protected from light and freezing, see the ***TELMA drug supply manual*** for details.

9.3. Unused trial drug supplies

If the IMPs are to be destroyed, it is the investigator's responsibility to ensure that arrangements have been made for disposal and that procedures for proper disposal have been established according to applicable regulations, guidelines, and institutional procedures. Appropriate records of the disposal must be maintained. Provide a certificate of destruction to Fundación GECP upon disposal. See the ***TELMA drug supply manual*** for details.

10. Trial treatments description

10.1. ATEZOLIZUMAB

Patients will receive 1200 mg of atezolizumab administered by IV infusion every 21 days (+/- 3 days) in a monitored setting where there is immediate access to trained personnel and adequate equipment/medicine to manage potentially serious reactions. Guidelines for treatment interruption or discontinuation and the management of specific adverse events are provided in Appendix 7.

Atezolizumab infusions will be administered per the instructions outlined in Table 1.

Table 1 Administration of First and Subsequent Infusions of Atezolizumab

First Infusion	Subsequent Infusions
• No premedication administered for atezolizumab specifically is permitted<br>• Record patient's vital signs within 60 minutes before starting infusion.<br>• Infuse atezolizumab (1200 mg in a 250 mL 0.9% NaCl IV bag) over 60 (± 15) minutes.<br>• If clinically indicated, record patient's vital signs during the infusion at 15, 30, 45, and 60 minutes (± 5-minute windows are allowed for all timepoints).<br>• If clinically indicated, record patient's vital signs at 30 (± 10) minutes after the infusion.<br>• Patients will be informed about the possibility of delayed post-infusion symptoms and instructed to contact their study physician if they develop such symptoms.	• If patient experienced infusion-related reaction during any previous infusion, premedication with antihistamines may be administered for Cycles ≥ 2 at the discretion of the treating physician.<br>• Record patient's vital signs within 60 minutes before starting infusion.<br>• If the patient tolerated the first infusion well without infusion-associated adverse events, the second infusion may be delivered over 30 (± 10) minutes.<br>• If no reaction occurs, subsequent infusions may be delivered over 30 (± 10) minutes.<br>Continue to record vital signs within 60 minutes before starting infusion. Record vital signs during and after the infusion, if clinically indicated.<br>• If the patient had an infusion-related reaction during the previous infusion, the subsequent infusion must be delivered over 60 (± 15) minutes.<br>Record patient's vital signs every 15 (± 5) minutes during the infusion if clinically indicated or patient experienced symptoms during the previous infusion.

NaCl = sodium chloride; Vital signs: heart rate, respiratory rate, blood pressure, and temperature.

Refer to ***TELMA drug supply manual*** for detailed instructions on drug preparation, storage, and administration.

10.2. BEVACIZUMAB

Patients will receive 15mg/Kg of Avastin® (bevacizumab) administered by IV infusion every 21 days in a monitored setting where there is immediate access to trained personnel and adequate equipment/medicine to manage potentially serious reactions (see Section 11.2).

Refer to ***TELMA drug supply manual*** for detailed instructions on drug preparation, storage, and administration.

11. Dose modifications criteria

Reasons for dose modifications, the supportive measures taken, and the outcomes will be documented in the patient's chart and recorded on the eCRF.

11.1. General Notes Regarding Dose Modification

- When several toxicities with different grades of severity occur at the same time, the dose modifications should be according to the highest grade observed.

- If, in the opinion of the investigator, a toxicity is considered to be due solely to one component of the study treatment (i.e., atezolizumab or bevacizumab) and the dose of that component is delayed or modified in accordance with the guidelines below, other components may be administered if there is no contraindication.

- When treatment is temporarily interrupted because of toxicity caused by bevacizumab or atezolizumab, the treatment cycles will be restarted such that the atezolizumab and bevacizumab infusions remain synchronized.

The investigator may use discretion in modifying or accelerating the dose modification guidelines described below depending on the severity of toxicity and an assessment of the risk versus benefit for the patient, with the goal of maximizing patient compliance and access to supportive care.

11.2. ATEZOLIZUMAB dose delay criteria

There will be no dose reduction for atezolizumab in this study. Patients may temporarily suspend study treatment for up to 105 days beyond the last dose if they experience an adverse event that requires a dose to be withheld. If atezolizumab is withheld because of adverse events for more than 105 days beyond the last dose, then the patient will be discontinued from atezolizumab treatment and will be followed for safety and efficacy.

If, in the judgment of the investigator, the patient is likely to derive clinical benefit from atezolizumab after a hold beyond 105 days, study drug may be restarted with the approval of trial chair/sponsor. If a patient must be tapered off steroids used to treat adverse events, atezolizumab may be withheld for additional time beyond 105 days from the last dose until steroids are discontinued or reduced to

prednisone dose (or dose equivalent) ≤ 10 mg/day. The acceptable length of interruption will depend on agreement between the investigator and the trial chair/sponsor.

Dose interruptions for reason(s) other than adverse events, such as surgical procedures, may be allowed with trial chair/sponsor approval. The acceptable length of interruption will depend on agreement between the investigator and the trial chair/sponsor. Given the mechanism of action of atezolizumab, events associated with inflammation and/or immune-related adverse events (irAEs) have been closely monitored during the atezolizumab clinical program. Guidelines for the management of patients who experience specific adverse events associated with atezolizumab (i.e., pulmonary, hepatic, gastrointestinal, endocrine, ocular, infusion-related [IRR], pancreatic, dermatologic, neurologic, or autoimmune events) are provided in Section 6 (Guidance for the Investigator) of the Atezolizumab Investigator's Brochure.

Systemic Immune Activation

Systemic immune activation is a rare condition characterized by an excessive immune response. Given the mechanism of action of atezolizumab, systemic immune activation is considered a potential risk when given in combination with other immunomodulating agents. Systemic immune activation should be included in the differential diagnosis for patients who, in the absence of an alternate etiology, develop a sepsis-like syndrome after administration of atezolizumab, and the initial evaluation should include the following:

CBC with peripheral smear
PT, PTT, fibrinogen, and D-dimer
Ferritin
Triglycerides
AST, ALT, and total bilirubin
LDH
Complete neurologic and abdominal examination (assess for hepatosplenomegaly)

If systemic immune activation is still suspected after the initial evaluation, contact the trial chair/sponsor for additional recommendations.

11.3. BEVACIZUMAB dose delay/reduction criteria

The dose of bevacizumab is calculated based on the body weight of the patient [kg]. If the body weight changes by 10% or more in the course of the treatment, dose adjustment is recommended for further infusions. If the body weight changes by less than 10%, no dose adjustment is needed.

- Blood pressure should be routinely measured before every infusion after a resting period of 10 min.
- Proteinuria should be routinely measured before every infusion of bevacizumab using dipstick.

- No routine premedication (e.g. to prevent infusion reaction) is recommended for bevacizumab.

The initial infusion of bevacizumab should be given over 90 minutes. If the initial infusion is well tolerated, the second infusion can be given over 60 minutes. If the second infusion is again well tolerated, all subsequent infusions can be given over 30 minutes.
In case of relevant toxicity, bevacizumab should be paused or discontinued.

Bevacizumab should be given every 3 weeks +/-3 days. If the administration was pre- or postponed up to 3 days the patient should (if possible) return to schedule and the next dose.
should be given 21 days after the last administration.

Management of bevacizumab related toxicities

No reductions in bevacizumab dose are allowed in this study.

Treatment with bevacizumab should be temporarily interrupted if one of the following adverse events occurs despite optimal supportive care, when not attributable to the disease under investigation, where the investigator considers the AE of concern to be specifically associated with bevacizumab:

- Any intolerable adverse event regardless of grade
- Any adverse events CTCAE V 5.0 grade ≥3 (despite optimal supportive care)

If toxicity resolves or reverts to CTCAE V5.0 grade ≤1 within 21 days of onset and the patient is showing clinical benefit, treatment with bevacizumab may be restarted using the rules below for dose modifications.

If toxicity does not resolve to CTCAE V 5.0 grade ≤1 after 42 days, then the patient must be permanently discontinued from bevacizumab.

The bevacizumab-related AEs hypertension, proteinuria, thromboembolism and hemorrhage including any CNS bleeding, as well as any grade 3 or 4 bevacizumab related AEs, should be managed as described below.

Event	CTCAE Version 5.0 Grade	Action to be Taken
Allergic reactions<br>or<br>Infusion-related reactions<br>Or<br>Anaphylaxis	Grade 1	Systemic intervention not indicated – continue bevacizumab
	Grade 2	Oral intervention indicated – slow infusion to 50% or interrupt if clinically indicated (re-start infusion at 50% and increase in 50% increments if well tolerated). Infusion can be

		re-started at the full rate for subsequent infusions.
	Grade 3	Bronchospasm (allergy-related oedema/angioedema; hypotension); hospitalization for clinical sequelae; intravenous intervention indicated – discontinue bevacizumab
	Grade 4	Life-threatening consequences; urgent intervention indicated - discontinue bevacizumab
Thromboembolic Event (arterial)	Any Grade	Discontinue bevacizumab
Thromboembolic Event (Venous)	Grade 3	Hold bevacizumab treatment. If the planned duration of full-dose anticoagulation is <2weeks, bevacizumab should be held until the full-dose anticoagulation period is over. The use of direct oral anticoagulants is not recommended.<br>If the planned duration of full-dose anticoagulation is >2 weeks, bevacizumab may be resumed during full-dose anticoagulation IF all of the criteria below are met:<br>- The patient must not have pathological conditions that carry high risk of bleeding (e.g. tumor involving major vessels or other conditions)<br>- The patient must not have had hemorrhagic events > grade 2 while on study<br>- The patient must be on stable dose of heparin, low molecular weight heparin, or have an in-range INR (usually 2-3) on a stable dose of warfarin prior to restarting bevacizumab.<br>If thromboemboli worsen/recur upon resumption of study therapy, discontinue bevacizumab
	Grade 4	Discontinue bevacizumab
Hypertension	[Treat with anti-hypertensive medication as needed. The goal of BP control should be consistent with general medical practice]	
	Grade 1<br>(SBP 120-139 mmHg or DBP80-89 mm Hg)	Consider increased BP monitoring; start anti-hypertensive medication if appropriate
	Grade 2 asymptomatic	Begin (or modify baseline anti-HTN therapy) anti-hypertensive therapy and continue bevacizumab

	(SBP 140-159 mmHg or DBP 90-99 mm Hg)	
	• Grade 2 symptomatic<br>(SBP 140-159 mmHg or DBP 90-99 mm Hg)	• Start or adjust anti-hypertensive medication
	• Grade 3<br>• (≥ SBP 160 mmHg or ≥ DBP 100 mmHg	• Modify existing anti-HTN therapy (more than one drug or more intensive therapy than previously indicated.<br>• Hold bevacizumab until symptoms resolve AND BP < 160/90mmHg
	Grade 4<br>(e.g. Hypertensive crisis or malignant hypertension)	Discontinue bevacizumab
Heart Failure or left ventricular dysfunction	Grade 3	Discontinue bevacizumab
	Grade 4	Discontinue bevacizumab
Proteinuria*	1+ proteinuria<br>(≥ ULN - <1.0g/24h)	Continue bevacizumab
	2+ and 3+ proteinuria<br>(1.0 - <3.5g/24h)	2+ - administer bevacizumab and obtain 24-hour urine protein before next administration<br>3+ - obtain 24-hour urine protein and administer bevacizumab if <2.0 g/24h
	4+ proteinuria<br>(≥ 3.5g/24h)	Obtain 24-hour urine protein and administer bevacizumab only when <2.0 g/24h
Nephrotic syndrome		Grade 3 or 4 Discontinue bevacizumab
Haemorrhage (CNS)	Any grade	Discontinue bevacizumab
Haemorrhage (haemoptysis)	Grade 1	Trace haemoptysis; continue bevacizumab
	Grade 2 - 4	≥2.5 mL bright red blood per episode; discontinue bevacizumab
Haemorrhage (other)	Grade 3 - 4	Discontinue bevacizumab
RPLS (Reversible Posterior Leukoencephalopathy syndrome or PRES (Posterior Reversible Encephalopathy Syndrome)		Discontinue bevacizumab
Wound dehiscence requiring medical or surgical intervention		Discontinue bevacizumab
Perforation (GI, or any other organ)		Discontinue bevacizumab
Fistula (GI, pulmonary or any other organ)		Discontinue bevacizumab

Obstruction of GI tract	G2 requiring medical intervention	Hold bevacizumab until complete resolution
	G3-4	Hold bevacizumab until complete resolution<br>If surgery is required, patient may restart bevacizumab after full recovery from surgery, and at investigator's discretion
Febrile neutropenia	Grade 3	Continue bevacizumab
	Grade 4	Hold bevacizumab until resolution or return to baseline
Platelet count decreased	Grades 1 - 3	Continue bevacizumab
	Grade 4	Hold bevacizumab until resolution or return to baseline
Other Unspecified bevacizumab-related AEs (except controlled nausea/vomiting).	Grade 3	Hold bevacizumab until symptoms resolve to ≤ grade 1 or baseline
	Grade 4	Discontinue bevacizumab<br>Upon consultation with the study chair/medical monitor, resumption of bevacizumab may be considered if a patient is benefiting from therapy, and the G4 toxicity is transient, has recovered to ≤ grade 1 (or baseline) and unlikely to recur with retreatment.

*Institutional protocols acceptable

Infusion of bevacizumab should be interrupted in patients who develop dyspnea or clinically significant hypotension. Patients who experience an NCI CTCAE Grade 3 or 4 allergic reaction/hypersensitivity, adult respiratory distress syndrome, or bronchospasm (regardless of grade) will be discontinued from bevacizumab treatment.

Bevacizumab infusion should be slowed to ≤ 50% or interrupted for patients who experience any infusion-associated symptoms not specified above. If the infusion is interrupted, it may be resumed at ≤ 50% of the rate prior to the reaction after the patient's symptoms have adequately resolved and increased in 50% increments up to the full rate if well tolerated. Infusions may be restarted at the full rate during the next cycle.

<u>Surgery and wound healing complications</u>

The appropriate interval between the last dose of bevacizumab and major surgery is unknown. Because bevacizumab has a half-life of approximately 21 days, elective surgery should be delayed whenever possible, but if necessary, bevacizumab should be withheld for ≥ 28 days prior to the procedure.

Bevacizumab may adversely affect the wound healing process. Bevacizumab therapy should not be initiated for at least 28 days following major surgery or until the surgical wound is fully healed. In patients who experience wound healing complications during bevacizumab treatment, bevacizumab

should be withheld until the wound is fully healed. Bevacizumab therapy should be withheld 4 weeks prior to elective surgery.

Necrotising fasciitis, including fatal cases, has rarely been reported in patients treated with bevacizumab. This condition is usually secondary to wound healing complications, gastrointestinal perforation or fistula formation. Bevacizumab therapy should be discontinued in patients who develop necrotising fasciitis, and appropriate treatment should be promptly initiated.

12. Prohibited and permitted concomitant treatments

Concomitant therapy consists of any medication (e.g., prescription drugs, over-the-counter drugs, vaccines, herbal or homeopathic remedies, nutritional supplements) used by a patient in addition to protocol-mandated treatment from 7 days prior to initiation of study drug to the study completion/discontinuation visit. All such medications should be reported to the investigator and recorded on the Concomitant Medications form in the eCRF.

<u>Prohibited Concomitant Therapy</u>

Any concomitant therapy intended for the treatment of cancer, whether health authority–approved or experimental, is prohibited for various time periods prior to starting study treatment, depending on the anti-cancer agent, and during study treatment until disease progression is documented and patient has discontinued study treatment. This includes, but is not limited to, chemotherapy, hormonal therapy, immunotherapy, radiotherapy, investigational agents, or herbal therapy (unless otherwise noted).

The following medications are prohibited while on study, unless otherwise noted:

- Denosumab; patients who are receiving denosumab prior to enrollment must be willing and eligible to receive a bisphosphonate instead while in the study.
- Any live or attenuated vaccine (e.g., FluMist®) within 4 weeks prior to enrollment or during treatment or within 30 days following the last atezolizumab dose
- Use of steroids to premedicate patients for whom CT scans with contrast are contraindicated (i.e., patients with contrast allergy or impaired renal clearance); in such patients, non-contrast CT scans of the chest and non-contrast CT scans or MRIs of the abdomen and pelvis should be performed.
- Metamizole (dipyrone) is prohibited in treating atezolizumab-associated IRRs because of its potential for causing agranulocytosis.

The concomitant use of herbal therapies is not recommended because their pharmacokinetics, safety profiles, and potential drug-drug interactions are generally unknown. However, their use for patients on study is allowed at the discretion of the investigator provided that there are no known

interactions with any study treatment. As noted above, herbal therapies intended for the treatment of cancer are prohibited.

Permitted Concomitant Therapy

Premedication with antihistamines may be administered for any atezolizumab infusions after Cycle 1. The following therapies should continue while patients are on study:

- Oral contraceptives.
- Hormone-replacement therapy.
- Prophylactic or therapeutic anticoagulation therapy, such as low molecular weight heparin (preferred) or warfarin at a stable dose level.
- Palliative radiotherapy (e.g., treatment of known bony metastases) provided it does not interfere with the assessment of tumor target lesions (e.g., the lesion being irradiated is not the only site of disease, as that would render the patient not evaluable for response by tumor assessments according to RECIST v1.1). It is not a requirement to withhold atezolizumab during palliative radiotherapy.
- Local therapy (e.g., surgery, stereotactic radiosurgery, whole brain radiotherapy) as outlined below:

  Patients experiencing a mixed response requiring local therapy for control may still be eligible to continue study treatment after Medical Monitor approval has been obtained. Patients who receive local therapy directed at a target lesion will no longer be evaluable for radiographic response but will remain evaluable for progression.

- Inactive influenza vaccinations.
- Megestrol administered as an appetite stimulant.
- Inhaled corticosteroids for chronic obstructive pulmonary disease.
- Mineralocorticoids (e.g., fludrocortisone).
- Low-dose corticosteroids for patients with orthostatic hypotension or adrenocortical insufficiency (e.g oral hidroaltesona 20 mg per day).
- G-CSF is allowed as per local standard.

In general, investigators should manage a patient’s care with supportive therapies as clinically indicated per local standards. Patients who experience infusion-associated symptoms may be treated symptomatically with acetaminophen, ibuprofen, diphenhydramine, and/or famotidine or another H2-receptor antagonist per standard practice. Serious infusion-associated events manifested by dyspnea, hypotension, wheezing, bronchospasm, tachycardia, reduced oxygen saturation, or respiratory distress should be managed with supportive therapies as clinically indicated (e.g., supplemental oxygen and β2-adrenergic agonists).

Cautionary Concomitant Therapy

Systemic corticosteroids and TNF-α inhibitors may attenuate potential beneficial immunologic effects of treatment with atezolizumab. Therefore, in situations where systemic corticosteroids or TNF-α inhibitors would be routinely administered, alternatives, including antihistamines, should be considered first by the treating physician. If the alternatives are not feasible, systemic corticosteroids and TNF-α inhibitors may be administered at the discretion of the treating physician except in the case of patients for whom CT scans with contrast are contraindicated (i.e., patients with contrast allergy or impaired renal clearance).

Systemic corticosteroids are recommended, with caution at the discretion of the treating physician, for the treatment of specific adverse events when associated with atezolizumab therapy. Guidelines for the management of immune-mediated adverse events are described in Appendix 7.

13. Adverse events and reporting

13.1. Definition of an Adverse Event (AE) and AE's reporting

The main criterion for tolerability is the occurrence of toxicities and Adverse Events (AEs). An AE is defined as any untoward medical occurrence that occurs from the subject's written consent to participate in the study through **30** days after the final administration of the IMP, regardless of whether it is considered related or not to the study drug. After informed consent has been obtained, but prior to initiation of study drug, only serious adverse events caused by a protocol-mandated intervention (e.g., invasive procedures such as biopsies, discontinuation of medications) should be reported.

An adverse event can therefore be any of the following:

Any unfavourable and unintended sign (including an abnormal laboratory finding) (see **NOTE 1** below), symptom, or disease temporally associated with the use of a medicinal product, whether or not considered related to the medicinal product.

Any new disease or exacerbation of an existing disease (a worsening in the character, frequency, or severity of a known condition), except events that are clearly consistent with the expected pattern of the disease progression. These data will be captured as efficacy assessment data only. If there is any uncertainty as to whether an event is due to disease progression, it should be reported as an adverse event.

Recurrence of an intermittent medical condition (e.g., headache) not present at baseline.

Any deterioration in a laboratory value or other clinical test (e.g., ECG, X-ray) that is associated with symptoms or leads to a change in study treatment or concomitant treatment or discontinuation from study drug.

Adverse events that are related to a protocol-mandated intervention, including those that occur prior to assignment of study treatment (e.g., screening invasive procedures such as biopsies).

Adverse events that occur during or within 24 hours after study drug administration and are judged to be related to study drug infusion should be captured as a diagnosis (e.g., "infusion-related reaction" or "anaphylactic reaction") on the Adverse Event eCRF. If a patient experiences both a local and systemic reaction to the same dose of study drug, each reaction should be recorded separately on the Adverse Event eCRF. Associated signs and symptoms should be recorded on the dedicated Infusion-Related Reaction eCRF.

A pre-existing medical condition should be recorded as an adverse event only if the frequency, severity, or character of the condition worsens during the study (e.g., “more frequent headaches”).

NOTE 1: Abnormal laboratory and vital signs values:

A laboratory test result must be reported as an adverse event if it is a change from baseline and meets any of the following criteria:

Is accompanied by clinical symptoms,

Results in a change in study treatment (e.g., dosage modification, treatment interruption, or treatment discontinuation),

Results in a medical intervention (e.g., potassium supplementation for hypokalaemia) or a change in concomitant therapy,

Is clinically significant in the investigator’s judgment.

If a clinically significant laboratory abnormality is not a sign of a disease or syndrome, the abnormality itself should be recorded on the Adverse Event eCRF, along with a descriptor indicating if the test result is above or below the normal range (e.g., "elevated potassium").

A vital sign result must be reported as an adverse event if it is a change from baseline and meets any of the following criteria:

Is accompanied by clinical symptoms,

Results in a change in study treatment (e.g., dosage modification, treatment interruption, or treatment discontinuation),

Results in a medical intervention or a change in concomitant therapy,

Is clinically significant in the investigator’s judgment.

If a clinically significant vital sign abnormality is a sign of a disease or syndrome (e.g., high blood pressure), only the diagnosis (i.e., hypertension) should be recorded on the Adverse Event eCRF.

The finding of an elevated ALT or AST (> 3 × baseline value) in combination with either an elevated total bilirubin (> 2 × ULN) or clinical jaundice in the absence of cholestasis or other causes of hyperbilirubinemia is considered to be an indicator of severe liver injury. Therefore, investigators must report as an adverse event the occurrence of either of the following:

Treatment-emergent ALT or AST > 3 × baseline value in combination with total bilirubin > 2 × ULN (of which ≥ 35% is direct bilirubin),

Treatment-emergent ALT or AST > 3 × baseline value in combination with clinical jaundice.

This abnormal laboratory values related to the liver function should be recorded on the Adverse Event eCRF and reported immediately as a SAE (AESI).

The severity will be classified according to the NCI CTCAE Version 5.0. The CTCAE is available for downloading on the internet (see appendix 1).

The AE severity grade provides a qualitative assessment of the extent or intensity of an AE, as determined by the investigator or as reported by the patients. The severity grade does not reflect the clinical seriousness of the event, only the degree or extent of the affliction or occurrence (e.g., severe nausea, mild seizure), and does not reflect the relationship to study drug.

Severity grade for other adverse events not covered in the toxicity grading scale:

1 = Grade 1	Mild
2 = Grade 2	Moderate
3 = Grade 3	Severe
4 = Grade 4	Life-threatening
5 = Grade 5	Fatal

However, depending on the SAE term, there is some AE that do not have all the grades (e.g., the SAE term Alopecia only has the Grade 1 and 2).

The causal relationship to study drug is determined by the physician and should be used to assess all AE. The casual relationship can be one of the following:

Related: There is a reasonable causal relationship between study drug administration and the AE.

Not related: There is not a reasonable causal relationship between study drug administration and the AE.

The term "reasonable causal relationship" means there is evidence to suggest a causal relationship.

About the AEs that have to be recorded in the eCRF note that:

- Baseline symptoms will be recorded on the eCRF and changes and updates in grade as well as resolution of an AE during treatment have to be reported,
- An AE has to be reported for each SAE notified,
- AEs should not be reported in a narrative description.

AEs can be spontaneously reported or elicited during open-ended questioning, examination, or evaluation of a subject (in order to prevent reporting bias, subjects should not be questioned regarding the specific occurrence of one or more AEs).

Any AE must be reported by an entry in the eCRF within 5 days of awareness, even if they do not meet the whole information. The diagnosis of the event should be recorded as AE term. If there is a secondary AE separated in time from the initiating event, it should be recorded as an independent event (e.g., if vomiting results in severe dehydration, both events should be reported separately).

13.2. Definition of Serious Adverse Event (SAE) and Suspected Unexpected Serious Adverse Reaction (SUSAR)

A Serious Adverse Event (SAE) is defined in general as any undesirable medical occurrence/adverse drug experience that occurs from the subject's written consent to participate in the study through **30** days after the final administration of the IMP, regardless of whether it is considered related or not to the study drug and that, at any dose, results in any of the following:

- results in death (fatal),
- is life-threatening (defined as an event in which the participant was at risk of death at the time of the event; it does not refer to an event which hypothetically might have caused death if it was more severe),
- requires inpatient hospitalization or causes prolongation of existing hospitalization (see **NOTE 2** below),
- results in persistent or significant disability/incapacity,
- is a congenital anomaly/birth defect,
- is an important medical event. Defined as a medical event(s) that may not be immediately life-threatening or result in death or hospitalization but, based upon appropriate medical and scientific judgment, may jeopardize the subject or may require intervention (e.g., medical, surgical, laboratory abnormal parameters…) to prevent one of the other serious outcomes listed in the definition above (see **NOTE 3** below),

After informed consent has been obtained, but prior to initiation of study drug, only serious adverse events caused by a protocol-mandated intervention (e.g., invasive procedures such as biopsies, discontinuation of medications) should be reported.

Seriousness is based on patient/event outcome or action criteria usually associated with events that pose a threat to a patient's life or functioning. Seriousness (not severity) serves as a guide for defining regulatory reporting obligations

NOTE 2: The following hospitalizations are not considered SAEs:

- A visit to the emergency room or other hospital department < 24 hours, that does not result in admission, except if it is considered as an important medical event or life- threatening,
- elective surgery, planned prior to signing consent,
- routine health assessment requiring admission for baseline/trending of health status (e.g., routine colonoscopy),
- admissions as per protocol for a planned medical/surgical procedure,
- admission for administration of anticancer therapy in the absence of any other SAEs,
- admission encountered for another life circumstance that carries no bearing on health status and requires no medical/surgical intervention (e.g., lack of housing, economic inadequacy, caregiver respite, family circumstances, administrative reason),
- progression of disease. By convention, clinical events related to the primary cancer being studied or to the primary cancer progression are not to be reported as SAEs, even if they meet any of the seriousness criteria from the standard SAE definition, unless the event is more severe than expected and therefore the investigator considers that their clinical significance deserves reporting.

NOTE 3: Any laboratory abnormalities should be documented as a SAE if:

- Intensive treatment in an emergency room or at home for allergic bronchospasm is needed,
- Blood dyscrasias or convulsions that do not result in hospitalization,
- Any untoward event related, and unexpected, to the protocol procedures,
- The laboratory test result is clinically significant or meets the definition of a SAE,
- The laboratory abnormality required the participant to have study drug discontinued or interrupted,
- The laboratory abnormality required the subject to receive specific corrective therapy.

A SAE report should be completed for any event where doubt exists regarding its seriousness. Although overdose, pregnancy, AESI (e.g., potential drug-induced liver injury (DILI)), important medical event, and cancer (secondary malignancy) are not always serious by regulatory definition, these events must be handled as SAEs. If applicable, SAEs must be collected that relate to any later protocol-specified procedure (e.g., a follow-up skin biopsy).

If the investigator believes that a SAE is not related to study drug but is potentially related to the conditions of the study (such as withdrawal of previous therapy or a complication of a study procedure), the relationship should be specified in the narrative section of the SAE Report Form and an important medical event would be the seriousness.

All deaths that occur during the reporting period that are clearly the result of disease progression should not be reported as a SAE, but they should be collected as an AE. All deaths where it is not due to disease progression and deaths with an unknown cause should always be reported as a SAE.

Death should be considered as an outcome and not the event, the AE causing the death must be reported as the SAE term. The term "sudden death" should be used only for the occurrence of an abrupt and unexpected death due to presumed cardiac causes in a patient with or without preexisting heart disease, within 1 hour after the onset of acute symptoms or, in the case of an unwitnessed death, within 24 hours after the patient was last seen alive and stable. If the cause of death is unknown and cannot be ascertained at the time of reporting, "Death due to Unknown Cause" should be recorded on the Adverse Event eCRF. If the cause of death later becomes available (e.g., after autopsy), the event should be replaced by the established cause of death.

If the SAE is considered as related to the study drug by the Principal Investigator, it is a Serious Adverse Reaction. The SAR is assessed by the Fundación GECP (SLCG/GECP) to know if it is expected. A SAR that is not listed as a known toxicity of the investigational drug in the summary of product characteristics (Investigator Brochure or Data Sheet) will be considered as a Suspected Unexpected Serious Adverse Reaction (SUSAR). The sponsor will inform to the Principal Investigator that this SAE has been considered as a SUSAR. In Spain, Health authority (AEMPS: Agencia Española del Medicamento y Productos Sanitarios) will also be informed about all SUSARs via EudraVigilance. Also, the autonomous regions will be informed about all SUSARs according to the current Spanish clinical trial regulation.

13.3. Adverse events of Special Interest for Atezolizumab (Immediately reportable to the Sponsor)

Adverse events of special interest are required to be reported by the investigator to the Fundación GECP (SLCG/GECP) immediately) (no more than 24 hours after learning of the event; see Section 13.5 for reporting instructions), despite not being serious. Adverse events of special interest for this study include the following:

- Cases of potential drug-induced liver injury that include an elevated ALT or AST in combination with either an elevated bilirubin or clinical jaundice, as defined by Hy's Law,
- Suspected transmission of an infectious agent by the study treatment, as defined below,
- Any organism, virus, or infectious particle (e.g., prion protein transmitting transmissible spongiform encephalopathy), pathogenic or non-pathogenic, is considered an infectious agent. A transmission of an infectious agent may be suspected from clinical symptoms or

laboratory findings that indicate an infection in a patient exposed to a medicinal product. This term applies only when a contamination of study treatment is suspected.

- Pneumonitis,
- Colitis,
- Endocrinopathies: diabetes mellitus, pancreatitis, adrenal insufficiency, hyperthyroidism, and hypophysitis,
- Hepatitis, including AST or ALT > 10 × ULN,
- Systemic lupus erythematosus,
- Neurological disorders: Guillain-Barré syndrome, myasthenic syndrome or myasthenia gravis, and meningoencephalitis,
- Events suggestive of hypersensitivity, infusion-related reactions, cytokine-release syndrome, influenza-like illness, systemic inflammatory response syndrome, and systemic immune activation,
- Nephritis,
- Ocular inflammatory toxicity (e.g., uveitis, retinitis, optic neuritis),
- Myositis,
- Myopathies, including rhabdomyolysis,
- Grade ≥ 2 cardiac disorders (e.g., atrial fibrillation, myocarditis, pericarditis),
- Vasculitis,
- Autoimmune haemolytic anaemia,
- Severe cutaneous reactions (e.g., Stevens-Johnson syndrome, dermatitis bullous, toxic epidermal necrolysis).

13.4. Overdose and secondary malignancies

An overdose is defined as the accidental or intentional administration of any dose of a product that is considered both excessive and medically important. All occurrences of overdose must be reported as a SAE, indicating “life-threatening” as the seriousness.

Secondary malignancies are not always serious by regulatory definition, these events must be handled as SAEs.

13.5. SAEs and SUSARs reporting

All SAEs that occur within **30** days of the last administered study medication must be collected. Any SAE must be reported by submitting the completed Initial SAE Form in the online eCRF within 24 hours of awareness, even if they do not meet any of the seriousness criteria.

In case of the eCRF is unavailability, the submission could be done by sending the SAE form preferably by email to pharmacovigilance.slcg@gecp.org or by fax to the Fundación GECP (SLCG/GECP) Pharmacovigilance Office: +34 93 419 17 68. An appropriate SAE form (see appendix 3), should be used to report SAEs to Fundación GECP (SLCG/GECP). The paper SAE form has to be sent within the

timeline period for reporting of 24 hours of awareness. **Once the eCRF system is available again, the SAE has to be completed and submitted by the site into the eCRF**.

If only limited information is initially available, a follow-up report is required to complete the information as soon as possible. (Note: Follow-up SAE reports should include the same SAE term(s) initially reported.)

The SAE outcome must be reported within 14 days after initial reporting by submitting the Follow up SAE Form in the online eCRF. In case the SAE is reported as ongoing after 14 days, a follow-up report has to be submitted again with the final outcome.

If an ongoing SAE changes in its grade, relationship to study drug, seriousness, SAE term or if new information becomes available, a new follow-up SAE report should be sent within 24 hours to the Fundación GECP (SLCG/GECP) using the same procedure after described.

Any SUSAR occurred during the trial will be notified under Spanish clinical regulation to the principal investigators, to the Health Authority (AEMPS) via Eudravigilance within the timelines specified in the Royal Decree 1090/2015 on clinical trials regarding the recording, evaluation and reporting of adverse events. Also, the autonomous regions will be informed about all SUSARs according to the current Spanish clinical trial regulation.

For any doubt please contact Fundation GECP (SLCG/GECP) pharmacovigilance office by this email: pharmacovigilance.slcg@gecp.org or by the telephone: +(34) 93 430 20 06.

Also, Fundación GECP (SLCG/GECP) will ensure that all SAEs, SUSARs, AESIs and pregnancy reports in the clinical database (eCRF) are reported to the authorization holder y during the conduct of the study including periodic reconciliation.

13.6. Pregnancy

If, following initiation of the investigational product, it is subsequently discovered that a study participant is pregnant or may have been pregnant at the time of investigational product exposure, within 5 months after the last dose of atezolizumab and/or bevacizumab, or within 6 months after the last dose of atezolizumab and/or bevacizumab, the investigational product will be permanently discontinued in an appropriate manner (e.g., dose tapering if necessary for participant).

Any pregnancy that occurs in a female partner of a male study participant should also be reported to the Fundación GECP (SLCG/GECP). Male patients will be instructed through the Informed Consent Form to immediately inform the investigator if his partner becomes pregnant during the chemotherapy treatment period or within 6 months after the last dose of chemotherapy. In order for Fundación GECP (SLCG/GECP) or designee to collect any pregnancy surveillance information from the female partner, the female partner must sign an informed consent form for disclosure of this information.

In both cases, the investigator must immediately notify to the Fundación GECP (SLCG/GECP) Pharmacovigilance Office by sending the Pregnancy form (see appendix 4) preferably by email to pharmacovigilance.slcg@gecp.org or by fax to the Fundación GECP (SLCG/GECP) Pharmacovigilance Office: +34 93 419 17 68.

Follow-up information regarding the course of the pregnancy, including perinatal and neonatal outcome and, where applicable, offspring information, study discontinuation must be reported following the before procedure described during at least 1 year after child-bearing.

Any serious adverse events associated with the pregnancy (e.g., an event in the fetus, an event in the mother during or after the pregnancy, or a congenital anomaly/birth defect in the child) or abortion should be reported as a SAE.

13.7. Other safety considerations

Any significant worsening noted during interim or final physical examinations, electrocardiograms, X-rays, and any other potential safety assessments, whether or not these procedures are required by the protocol, should also be recorded as a non-serious or serious AE, as appropriate, and reported accordingly.

13.8. Adverse events related to the treatment and associated risks

ATEZOLIZUMAB

The PD-L1/PD-1 pathway is involved in peripheral tolerance; therefore, such therapy may increase the risk of immune-mediated adverse events (ir-AE), specifically the induction or enhancement of autoimmune conditions. Adverse events with potentially immune-mediated causes, including rash, hypothyroidism, hepatitis/transaminitis, colitis, pneumonitis, myositis, and myasthenia gravis, have been observed in the Phase Ia study PCD4989g.

Although most immune-mediated adverse events observed with immunomodulatory agents have been mild and self-limiting, such events should be recognized early and treated promptly to avoid potential major complications.

Given the mechanism of action of atezolizumab, events associated with inflammation and/or immune-related adverse events (irAEs) have been closely monitored during the atezolizumab clinical program. These include potential dermatologic, hepatic, endocrine, gastrointestinal and respiratory events. Refer to the Atezolizumab Investigator's Brochure for details on irAEs that were observed in patients treated with atezolizumab.

BEVACIZUMAB

Warnings and precautious for bevacizumab include perforation or fistula; surgery and wound healing complications; hemorrhage; arterial thromboembolic events; venous thromboembolic events;

hypertension; posterior reversible encephalopathy syndrome (PRES); proteinuria; infusion reactions; ovarian failure. The most common adverse reactions observed in bevacizumab patients at a rate >10% and at least twice the control arm rate, are epistaxis, headache, hypertension, rhinitis, proteinuria, taste alteration, dry skin, rectal hemorrhage, lacrimation disorder, back pain and exfoliative dermatitis (see SPC for details).

In a phase II study of erlotinib in combination with bevacizumab as first line therapy for EGFRm NSCLC patients, no new safety signals were identified between erlotinib plus bevacizumab group and erlotinib alone group, with hypertension (60% vs 10%) and proteinuria (8% vs 0%) being the more frequently observed grade 3 AEs in the combination group compared against erlotinib alone group.

Based on the identified and potential risks associated with bevacizumab, this trial protocol incorporates mandatory safety monitoring procedures and guidance to assist with early diagnosis and rapid management of potential drug-related symptoms.

The following NCI CTC grade 3 - 5 adverse reactions have been reported:

Very common (≥10%): Febrile neutropenia, leucopenia, thrombocytopenia, neutropenia, anorexia, peripheral sensory neuropathy, dysarthria, headache, dysguesia, eye disorder, lacrimeation increased, hypertension, thromboembolism, dyspnoea, rhinitis, rectal haemorrhage, stomatitis, constipation, diarrhoea, nausea, vomiting, abdominal pain, wound healing complications, exfoliative dermatitis, dry skin, skin discoloration, arthralgia, proteinuria, ovarian failure, asthenia, fatigue, pyrexia, pain, mucosal inflammation, weight decreased.

Common (1 – <10%): Sepsis, abscess, cellulitis, infection, urinary tract infection, anaemia, lymphopenia, hypersensitivity, infusion reaction, dehydration, cerebrovascular accident, syncope, somnolence, headache, congestive heart failure, supraventricular tachycardia, arterial thromboembolism, deep vein thrombosis, haemorrhage, pulmonary haemorrhage, haemoptysis, pulmonary embolism, dysphonia, hypoxia, epistaxis, gastrointestinal perforation, intestinal perforation, ileus, intestinal obstruction, recto-vaginal fistulae, gastrointestinal disorder, proctalgia, palmar-plantar erythrodysaesthesia syndrome, fistula, muscular weakness, myalgia, back pain, pelvic pain, lethargy.

14. Biological material and translational research

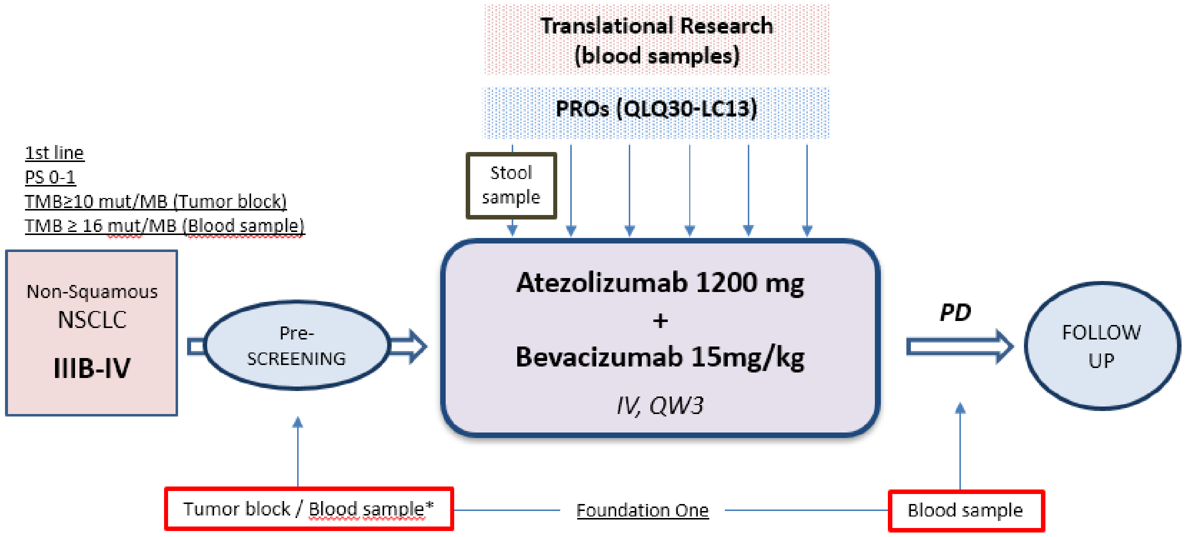

* If the submitted tumor sample is insufficient or not valid to evaluate the TMB, a blood sample can be sent instead .

Biological Sample Collection (Foundation one):

1. **FFPE tumor tissue**: At pre-screening. Primary tumor and/or the recurrence/metastatic biopsy will be obtained from a primary tumor or metastatic site. A minimum of 10 slides 4-5 um thick, with at least 20% tumor cellularity will be necessary for FoundationOne CDx™ (F1CDx).

   Tissue samples will be analyzed by different methods as IHC and Hybrid-capture NGS based comprehensive genome sequencing by Foundation Medicine. These exploratory analyses aim to study tumor-associated alterations to further understand disease pathobiology (including but not limited to mechanisms of disease progression, pseudo-progression, acquired resistance), to evaluate surrogate biomarkers and to potentially allow for the development of tissue-based diagnostic tests to help predict which patients may benefit from Atezolizumab in combination with Bevacizumab vs Atezolizumab alone.

   - Comprehensive genomic profile (CGP): High-intermediate tumor mutational burden (TMB) is an emerging biomarker of sensitivity to immune checkpoint inhibitors and has been shown to be significantly associated with response to PD-1 and PD-L1 blockade immunotherapy. CGP assay targeting ~0.8 Mb of coding genome measure with F1CDx (Foundation Medicine) can accurately assess TMB compared with sequencing the whole exome. F1CDx® is a comprehensive genomic profile that applies next- generation sequencing in a unique manner to identify all 4 types of genomic alterations across all genes known to be unambiguous drivers of solid tumous with high accuracy. The test simultaneously sequences the coding region of 324 cancer-

related genes plus introns from 29 genes often rearranged or altered in cancer to a typical median depth of coverage of greater than 500X.

If the submitted tumor sample is insufficient or not valid and a biopsy is not feasible to be performed to evaluate the TMB, a blood sample can be sent instead. A permission from the trial chair/sponsor must be requested in case no tumor sample is available for a possible patient and blood sample want to be sent to analyze the TMB.

2. **Blood samples for ctDNA**: At progression, two 10 ml tubes of blood sample will be needed, to perform ctDNA analysis with Foundation One Liquid panel.

FoundationACT is a blood-based circulating tumor DNA (ctDNA) assay for solid tumors that identifies clinically relevant genomic alterations driving the growth of a patient's cancer. Test results provide information about potential targeted therapies. Foundation One Liquid is validated to detect all 4 classes of genomic alterations and to analyse more than 70 of the most commonly mutated genes in solid tumors using a blood sample.
In a retrospective analysis suggest that Nivolumab improves ORR and PFS compared with platinum doublet chemotherapy in patients with high TMB (Peters et al. Abs CTMS02 AACR 2017).

CGP of lung cancer to simultaneously determine TMB, MSI status, PD-L1 amplification, and the presence of driver alterations may provide clinically useful predictors of response to ICPI and other targeted therapies (Chalmers et al. Genome Medicine 2017; Spigel et al. J Clin Oncol 2016).

<u>Biological Sample Collection (Pharmacogenomic exploratory analysis):</u>

Blood samples for the pharmacogenomic exploratory analysis will be drawn at:

- Baseline or before day 1 of cycle 1 treatment
- First CT-scan evaluation (cycle 5 if there is no delay in treatment administration)
- Second CT-scan evaluation (cycle 9 if there is no delay in treatment administration)
- Fourth CT-scan evaluation (cycle 17 if there is no delay in treatment administration)
- Sixth CT-scan evaluation (cycle 25 if there is no delay in treatment administration)
- Progression disease, either if it occurs during the bevacizumab + atezolizumab treatment or in follow up phase 21

Also, stool samples will be collected at baseline. Also, data at baseline of PDL1 status provided by the sites will be analyzed.

Please, refer to the ***TELMA Samples Manual*** for more information.

15. Case report forms and documentation

eCRFs will only be available on-line at the Remote Data Entry (RDE) facility of Fundación GECP for patient enrollment.

Only the patient pre-screening form will be used in a paper form and will be send scanned by email or by fax (+34 93 419 17 68) to obtain the pre-screening number.

Also, another exception will be a paper SAE form and pregnancy form that will be used only in case of eCRF system unavailability.

16. Statistical considerations

Three populations will be considered for different analyses:

- Per protocol population (PP): Per protocol population will consider patients that will receive a minimum of two cycles of atezolizumab and Bevacizumab or that have at least the first tumor response evaluation carried out. Patients without any radiological evaluation who may die during the first 12 weeks will also be considered PP population. Patients without the first tumor response evaluation carried out but with a progression disease (also clinical progression) at 6 weeks will also be considered into PP population.
- Intention to treat analysis (ITT): Intention to treat analysis will include all patients that will be registered into the clinical trial.
- Safety population (SFP): Safety population will include all patients that will be exposed to study treatment (atezolizumab + bevacizumab), whatever will be the quantity received

The primary endpoint is Progression Free Survival at 12 months (PFS) according to Response Evaluation Criteria in Solid Tumors (RECIST) v1.1.

For one arm, we estimate to achieve a PFS at 12 months of 40% (vs 18% from Chemotherapy, previous published studies), with a 90% power and alpha 5%, one-sided test. The test statistic for survival probability is based on the non-parametric estimate of the survival distribution. Additionally, estimating a 10% of errors, withdrawals or others, it will be necessary to recruit 40 patients in order to get the study objectives.

If the withdrawals are higher than 5%, so that in order to reach the proposed sample size, if a patient initially enrolled in the study does not fulfil the inclusion criteria, they will be replaced by a new one subject that fulfil them, this replacement will ensure that the sample size will be the one calculated initially.

<u>Data analysis and statistical methods</u>

A descriptive analysis will be made of the variables calculating absolute and relative frequencies of qualitative variables and mean(standard deviation) or median (25 and 75 percentiles), minimum and maximum values for quantitative variables.

For the primary endpoint analysis, survivor function estimated by Kaplan-Meier method will be used, as well as the survival curves.. The 95% confidence interval will be estimated.

For the bivariate analysis, appropriate parametric and nonparametric tests will be used depending on whether the data are normally or non-normally distributed. For comparison of quantitative variables, Student's t test for independent data, the Mann-Whitney U test, analysis of variance or the Kruskal-Wallis H test will be used.

17. Criteria for termination of the trial

17.1. General criteria for termination of the trial

The trial may be discontinued early in parts or completely if the information on the product leads to doubt as to the benefit/risk ratio, by decision of Fundación GECP or Trial Steering Committee, or at the suggestion of the IDMC.

The trial can be terminated at any time if the authorization and approval to conduct the study is withdrawn by ethics committee or regulatory authority decision, insufficient accrual, emerging new data impacting the scientific value of the trial or on ethical grounds.

17.2. Discontinuation of protocol treatment and from the study for individual patients

Patients may be withdrawn from the protocol treatment and from the study in the following situations:

- Occurrence of unacceptable toxicities. Stopping protocol treatment is determined by medical judgment of the treating physician.
- Disease progression per investigator assessment according to RECIST v1.1 and loss of clinical benefit
- Symptomatic deterioration attributed to clinical disease progression
- Inter-current severe illnesses which would in the judgment of the investigator affect assessments of the clinical status to a significant degree and require discontinuation of protocol therapy
- Request by the patient. Patients have the right to refuse further trial treatment at any time during the trial. Such patients will remain in the trial and will be transferred to the follow-up phase.

- If a patient refuses to have follow-up examinations and tests needed to determine whether the treatment is safe and effective
- Protocol non-compliance or study termination by sponsor or site closure by the sponsor (poor protocol adherence, inaccurate or incomplete data recording…)
- Pregnancy
- Patient non-compliance, defined as a failure to comply with the protocol requirements as determined by the protocol

The decision for discontinuation of protocol treatment and the study of individual patients is taken by the treating physician based on his medical evaluation and taking into account the patient's individual situation.

Specific reasons for trial discontinuation where further collection of data is not allowed are: withdrawal of consent, patient lost to follow up, death.

All possible measures will be undertaken to maintain the investigation program and to continue the follow-up even if the treatment was prematurely concluded and/or if the patient did not attend the follow-up visits at the participating institution.

The primary reason for the treatment and study discontinuation should be documented on the appropriate eCRF.

18. Ethics aspects, regulatory approval, and Patient Informed Consent

The Investigator will ensure that this study is conducted in full conformance with the principles of the "Declaration of Helsinki" or with the laws and regulations of the country in which the research is conducted, whichever affords the greater protection to the individual. The study must fully adhere to the principles outlined in "Guideline for Good Clinical Practice" ICH6 Tripartite Guideline (January 1997) or local Spanish law if it affords greater protection to the patient. For studies conducted in the EU/EEA countries, the Investigator will ensure compliance with the EU Clinical Trial Directive (2001/20/EC).

18.1. Ethical Review Board/Ethics Committee/Health Authority

All protocols and the patient informed consent forms must have the approval of a properly constituted committee or committees responsible for approving clinical trials. The Ethic Committee decision must contain approval of the designated investigator, the protocol (identifying protocol title and version number), and of the patient informed consent.

The Ethic Committee written, signed approval letter/form must contain approval of the designated investigator, the protocol (identifying protocol title and version number), and of the patient informed consent.

Any modifications made to the protocol must be submitted to the appropriate Ethic Committee for information or approval in accordance with local procedures and regulatory requirements and to Health Authorities if required.

Once approved or acknowledged by the appropriate ERB/IRB and by the Health Authorities (if required), the investigator shall implement the protocol modifications.

If applicable, in addition to the approval of the Ethics Committee according to national legislation, the protocol, other protocol related documents including patient information and informed consent and other documents as required locally must be submitted to and be approved by the health authority.

18.2. Informed consent

Both Informed consent (pre-screening IC and IC for the study) for each patient will be obtained prior to initiating any trial procedures in accordance with the "Patient Information and Informed Consent". Once signed and dated, a copy of the informed consent must be given to each patient and the original copy must be retained in the investigator's site file. The informed consent form must be available in the case of data audits.

The "Declaration of Helsinki" recommends that consent be obtained from each potential patient in biomedical research trials after the aims, methods, anticipated benefits, and potential hazards of the trial, and discomfort it may entail, are explained to the individual by the physician. The potential patient should also be informed of her/his right to not participate or to withdraw from the trial at any time. The patient should be told that material from her/his tumor and blood and serum samples will be stored and potentially used for additional studies not described in this protocol.

If the patient is in a dependent relationship to the physician or gives consent under duress, the informed consent should be obtained by an independent physician. It is preferable that the patient who gives the consent signs the form but if it is not possible or if the patient is legally incompetent (i.e., a minor, or mentally incompetent), informed consent must be obtained from the parent, legal guardian, or legal representative in accordance with the law of the country in which the trial is to take place.

By signing this protocol, the investigator agrees to conduct the trial in accordance with Good Clinical Practice and the "Declaration of Helsinki".

The template Patient Information Sheet and Informed Consent has been written according to ICH guidelines which state the Informed Consent should adhere to GCP and to the ethical principles that have origin in the "Declaration of Helsinki".

19. Governance and administrative issues

19.1. Study documentation

The investigator must maintain adequate and accurate records to enable the conduct of the study to be fully documented, including, but not limited to, the protocol, protocol amendments, Informed Consent Forms, and documentation of IRB/EC and governmental approval.

19.2. Protocol non-compliances/deviations

The investigator should document and explain any protocol non-compliances/deviations. The investigator should promptly report any non-compliances/deviations that might have an impact on patient safety and data integrity to the Sponsor and to the IRB/EC in accordance with established IRB/EC policies and procedures. The Sponsor will review all protocol non-compliances/deviations and assess whether any represent a serious breach of Good Clinical Practice guidelines and require reporting to health authorities. All protocol non-compliances/deviations will be recorded by the monitoring team.

19.3. Protocol amendment

Any protocol amendments will be prepared by the Sponsor. Protocol amendments will be submitted to the IRB/EC and to regulatory authorities in accordance with local regulatory requirements. Approval must be obtained from the IRB/EC and regulatory authorities (as locally required) before implementation of any changes, except for changes necessary to eliminate an immediate hazard to patients or changes that involve logistical or administrative aspects only (e.g., change in Medical Monitor or contact information).

19.4. Final report

A final clinical trial report will be written and distributed to the Ethic committees and Health Authorities as required by applicable regulatory requirements.

19.5. Independent Data Monitoring Committee

The trial will be presented periodically for review to the Fundación GECP BICR (blinded independent central review).

19.6. Publication

Authorship on the final manuscript or publications or provisional extracts will be decided in accordance with the Fundación GECP publication and authorship guidelines (SOP GECP: Política de publicaciones y auditorías).
None of the participants will present data to his centre in isolation from the rest of the results of the study and will need to seek approval from the sponsor.

19.7. Financial disclosure

Investigators will provide the sponsor with enough, accurate financial information in accordance with local regulations to allow the Sponsor to submit complete and accurate financial certification or disclosure statements to the appropriate health authorities. Investigators are responsible for providing information on financial interests during the course of the study and for 1 year after completion of the study.

19.8. Clinical trial insurance

Spanish legislation demands cover with a civil responsibility policy for subjects participating in a clinical trial. Fundación GECP will contract the appropriate liability insurance for this trial. Patients who suffer injuries due to the trial should report them immediately to their physician.

19.9. Quality assurance

Fundación GECP conducts trials according to the ICH Good Clinical Practice (GCP) guidelines. The Trial Data Manager reviews each eCRF as per monitoring plan schedule.

Fundación GECP will conduct periodic triggered visits to the participating sites to ensure proper trial conduct, verify compliance with GCP, and perform source data verification.

The Investigator should ensure that source documents are made available to appropriately qualified personnel from Fundación GECP, or to ethics committee and health authority inspectors after appropriate notification.

At regular intervals during the clinical trial, the centre will be contacted, through monitoring visits, letters or telephone calls, by a representative of the Monitoring Team to review study progress, investigator and patient compliance with clinical trial protocol requirements and any emergent problems. These monitoring visits will include but not be limited to review of the following aspects: patient informed consent, patient recruitment and follow-up, SAE documentation and reporting, AEs with pre-specified monitoring documentation and reporting, AE documentation, dispensing IMP, compliance with protocol, drug accountability, concomitant therapy use, quality of data and storage of blood and serum samples.

19.10. Data protection

The samples and data collected will be coded to protect patient confidentiality. Each patient will have a pre-screening number at the pre-screening phase to identify the biological material and a unique identifier assigned by the eCRF of the study once enrolled. Sites are responsible to keep a patient log locally in order to be able to link the pre-screening number and the unique identifier to the record of the patient.

No identifiable / personal data will be stored in the trial database or the tissue repositories in the central laboratories.

Regulatory authorities and pertinent Ethics Committees (IRB/ERB) may have access to patient data on-site. Fundación GECP audit or monitoring personnel will also have access to such data on-site.

In Spain to ensure the patient confidentiality of data applies from the 25th of May 2018 the Reglamento (UE) 2016/679 del Parlamento europeo y del Consejo de 27 de abril de 2016 de Protección de Datos (RGPD).

19.11. Study monitoring team

The Fundación GECP will be responsible for monitoring the trial at Spanish sites.

The clinical monitor is obliged to rigorously follow the study. For this, the clinical monitor will regularly visit the study centers and the investigators as well as maintain necessary written and telephone communications.

The clinical monitor will assess the data collected in the acquisition forms and compare them with the original data of the clinical history and other original documents in conjunction with the study investigator.

The contact persons will be:

Montse Cairó
Fundación GECP CRA
Meridiana 358, 6ª planta
08027 Barcelona
Tel. 93 430 20 06
Fax. 93 419 17 68
Email: mcairo@gecp.org

Ana Verdú
Fundación GECP Lead CRA
Meridiana 358, 6ª planta
08027 Barcelona
Tel. 93 430 20 06
Fax. 93 419 17 68
Email: averdu@gecp.org

19.12. Record retention

The centre must retain all essential documents according to ICH GCP. This includes copies of the patient trial records, which are considered as source data, patient informed consent statement,

laboratory printouts, drug inventory and destruction logs, and all other information collected during the trial. These documents are to be stored until at least 25 years after the termination of the trial.

In the event that the Principal Investigator retires or changes employment, custody of the records may be transferred to another competent person who will accept responsibility for those records. Written notice of such transfer must be given to the Fundación GECP and the local Ethics Committee at least 1 month in advance.

20. References

1. Ferlay J, Soerjomataram I, Dikshit R, Eser S, Mathers C, Rebelo M, et al. Cancer incidence and mortality worldwide: Sources, methods and major patterns in GLOBOCAN 2012. Int J Cancer. 2015 Mar 1;136(5): E359–86.
2. Youlden DR, Cramb SM, Baade PD. The International Epidemiology of Lung Cancer: Geographical Distribution and Secular Trends. J Thorac Oncol. 2008 Aug;3(8):819–31.
3. Alan Sandler, M.D., Robert Gray, Ph.D., Michael C. Perry, M.D., Julie Brahmer, M.D., Joan H. Schiller, M.D., Afshin Dowlati, M.D., Rogerio Lilenbaum, M.D., and David H. Johnson, M.D. N Engl J Med 2006;355:2542-50.
4. Suzanne L. Topalian, M.D., F. Stephen Hodi, M.D., Julie R. Brahmer, M.D., Scott N. Gettinger, M.D.,David C. Smith, M.D., David F. McDermott, M.D., John D. Powderly, M.D., Richard D. Carvajal, M.D., Jeffrey A. Sosman, M.D., Michael B. Atkins, M.D., Philip D. Leming, M.D., David R. Spigel, M.D., Scott J. Antonia, M.D., Ph.D., Leora Horn, M.D., Charles G. Drake, M.D., Ph.D., Drew M. Pardoll, M.D., Ph.D.,Lieping Chen, M.D., Ph.D., William H. Sharfman, M.D., Robert A. Anders, M.D., Ph.D., Janis M. Taube, M.D., Tracee L. McMiller, M.S., Haiying Xu, B.A., Alan J. Korman, Ph.D., Maria Jure-Kunkel, Ph.D., Shruti Agrawal, Ph.D., Daniel McDonald, M.B.A., Georgia D. Kollia, Ph.D., Ashok Gupta, M.D., Ph.D., Jon M. Wigginton, M.D., and Mario Sznol, M.D. N Engl J Med 2012;366:2443-54.
5. F. Stephen Hodi, M.D., Steven J. O'Day, M.D., David F. McDermott, M.D., Robert W. Weber, M.D., Jeffrey A. Sosman, M.D., John B. Haanen, M.D., Rene Gonzalez, M.D., Caroline Robert, M.D., Ph.D., Dirk Schadendorf, M.D., Jessica C. Hassel, M.D., Wallace Akerley, M.D., Alfons J.M. van den Eertwegh, M.D., Ph.D., Jose Lutzky, M.D., Paul Lorigan, M.D., Julia M. Vaubel, M.D., Gerald P. Linette, M.D., Ph.D., David Hogg, M.D., Christian H. Ottensmeier, M.D., Ph.D., Celeste Lebbe, M.D., Christian Peschel, M.D., Ian Quirt, M.D., Joseph I. Clark, M.D., Jedd D. Wolchok, M.D., Ph.D., Jeffrey S. Weber, M.D., Ph.D., Jason Tian, Ph.D., Michael J. Yellin, M.D., Geoffrey M. Nichol, M.B., Ch.B., Axel Hoos, M.D., Ph.D., and Walter J. Urba, M.D., Ph.D. N Engl J Med 2010;363:711-23.
6. Philip W. Kantoff, Thomas J. Schuetz, Brent A. Blumenstein, L. Michael Glode, David L. Bilhartz, Michael Wyand, Kelledy Manson, Dennis L. Panicali, Reiner Laus, Jeffrey Schlom, William L. Dahut, Philip M. Arlen, James L. Gulley, and Wayne R. Godfrey. J Clin Oncol 28:1099-1105.

7. Daniel S. Chen and Ira Mellman. Immunity, 2013 – Volume 39, Issue 1, 25 July 2013, pages 1-10.

8. Achim Rittmeyer, Fabrice Barlesi, Daniel Waterkamp, Keunchil Park, Fortunato Ciardiello, Joachim von Pawel, Shirish M Gadgeel, Toyoaki Hida, Dariusz M Kowalski, Manuel Cobo Dols, Diego L Cortinovis, Joseph Leach, Jonathan Polikoff , Carlos Barrios, Fairooz Kabbinavar, Osvaldo Arén Frontera, Filippo De Marinis, Hande Turna, Jong-Seok Lee, Marcus Ballinger, Marcin Kowanetz, Pei He, Daniel S Chen, Alan Sandler, David R Gandara, for the OAK Study Group. Published online December 12, 2016 http://dx.doi.org/10.1016/S0140-6736(16)32517-X.

9. Jamie Chaft, Bo Chao, Wallace Akerley, Michael S. Gordon, Scott J. Antonia, Jason Callahan, Alan Sandler, Roel Funke, Larry Leon, Jill Fredrickson, Marcin Kowanetz, Scott Gettinger. *Evaluation of PD-L1 expression in metachronous tumor samples and FDG-PET as a predictive biomarker in Ph2 study (FIR) of atezolizumab (MPDL3280A).* WCLC 2015, Oral.

10. Marina C. Garassino, Naiyer A. Rizvi, Benjamin Besse, Pasi A. Jänne, Daniel Christoph, Solange Peters, Chee Keong Toh, Takayasu Kurata, Enric Carcereny Costa, Marianna Koczywas, Enriqueta Felip, Jamie Chaft, Jiaheng Qiu, Marcin Kowanetz, Shelley Coleman, Simonetta Mocci, Alan Sandler, Scott Gettinger, Melissa L. Johnson. *Atezolizumab as 1L therapy for advanced NSCLC in PD-L1–selected patients: updated ORR, PFS and OS data from the BIRCH study.* WCLC 2016, Oral.

11. Martin Reck, M.D., Ph.D., Delvys Rodriguez-Abreu, M.D., Andrew G. Robinson, M.D., Rina Hui, M.B., B.S., Ph.D., Tibor Csőszi, M.D., Andrea Fulop, M.D., Maya Gottfried, M.D., Nir Peled, M.D., Ph.D., Ali Tafreshi, M.D., Sinead Cuffe, M.D., Mary O'Brien, M.D., Suman Rao, M.D., Katsuyuki Hotta, M.D., Ph.D., Melanie A. Leiby, Ph.D., Gregory M. Lubiniecki, M.D., Yue Shentu, Ph.D., Reshma Rangwala, M.D., Ph.D., and Julie R. Brahmer, M.D. N Engl J Med 375;19 nejm.org November 10, 2016.

12. Filippo De Marinis, Jacek Jassem, David R. Spigel, Sivuonthanh Lam, Simonetta Mocci, Alan Sandler, Ariel Lopez-Chavez, Yu Deng, Giuseppe Giaccone, Roy S. Herbst. *IMpower110: Phase III Trial Comparing 1L Atezolizumab with Chemotherapy in PD-L1–Selected Chemotherapy-Naive NSCLC Patients.* WCLC 2016, Poster # P3.02c-042.

13. Anticancer Res. 2001 Nov-Dec;21(6B):4373-82. Prognostic role of angiogenesis in non-small cell lung cancer. Giatromanolaki A1.

14. D. Br attström, M.Bergqv ist, P.Hesselius, A.Larsson, G.W agenius, O.Brodin. Lung Cancer (2004) 43, 55—62.

15. Caroline Bouzin, Olivier Feron. Drug Resistance Updates 10 (2007) 109–120.

16. Christian Manegold, MD, Anne-Marie C. Dingemans, MD, PhD, Jhanelle E. Gray, MD, Kazuhiko Nakagawa, MD, Marianne Nicolson, MD, Solange Peters, MD, PhD, Martin Reck, MD, PhD, Yi-Long Wu, MD, Odd Terje Brustugun, MD, PhD, Lucio Crinò, MD, Enriqueta Felip, MD, Dean

Fennell, MD, PhD, Pilar Garrido, MD, PhD, Rudolf M. Huber, MD, Aurélien Marabelle, MD, PhD, Marcin Moniuszko, MD, Françoise Mornex, MD, PhD, Silvia Novello, MD, PhD, Mauro Papotti, MD, Maurice Pérol, MD, Egbert F. Smit, MD, PhD, Kostas Syrigos, MD, Jan P. van Meerbeeck, MD, Nico van Zandwijk, MD, PhD, James Chih-Hsin Yang, MD, Caicun Zhou, MD, PhD, Everett Vokes, MD. Journal of Thoracic Oncology Vol. 12 No. 2: 194-207.

17. Huang Y, Chen X, Dikov MM, et al. Distinct roles of VEGFR-1 and VEGFR-2 in the aberrant hematopoiesis associated with elevated levels of VEGF. Blood 2007;110:624−31.

18. M.A. Socinski, R.M. Jotte, F. Cappuzzo, F. Orlandi, D. Stroyakovskiy, N. Nogami, D. Rodriguez-Abreu, D. Moro-Sibilot, C.A. Thomas, F. Barlesi, G. Finley, C. Kelsch, A. Lee, S. Coleman, Y. Deng, Y. Shen, M. Kowanetz, A. Lopez-Chavez, A. Sandler, and M. Reck, for the IMpower150 Study Group. N Engl J Med 2018;378:2288-301.

19. Robert Motzer, Thomas Powles, Michael Atkins, Bernard Escudier, David McDermott, Cristina Suarez, Sergio Bracarda, Walter M. Stadler, Frede Donskov, Jae Lyun Lee, Robert Hawkins, Alain Ravaud, Boris Alekseev, Michael Staehler, Motohide Uemura, Francis Donaldson, Shi Li, Mahrukh Huseni, Christina Schiff, Brian Rini. *IMmotion151: A Randomized Phase III Study of Atezolizumab Plus Bevacizumab vs Sunitinib in Untreated Metastatic Renal Cell Carcinoma.* GUCS 2018, Oral.

20. Ludmil B. Alexandrov, Young Seok Ju, Kerstin Haase, Peter Van Loo, Iñigo Martincorena, Serena Nik-Zainal, Yasushi Totoki, Akihiro Fujimoto, Hidewaki Nakagawa, Tatsuhiro Shibata, Peter J. Campbell, Paolo Vineis, David H. Phillips, Michael R. Stratton. Science VOL 354 ISSUE 6312.

21. Ludmil B. Alexandrov, Serena Nik-Zainal, David C. Wedge, Samuel A. J. R. Aparicio, Sam Behjati, Andrew V. Biankin, Graham R. Bignell, Niccolo` Bolli, Ake Borg, Anne-Lise Børresen-Dale, Sandrine Boyault, Birgit Burkhardt, Adam P. Butler, Carlos Caldas, Helen R. Davies, Christine Desmedt, Roland Eils, Jo´runn Erla Eyfjo¨rd, John A. Foekens, Mel Greaves, Fumie Hosoda, Barbara Hutter, Tomislav Ilicic, Sandrine Imbeaud, Marcin Imielinski, Natalie Ja¨ger, David T. W. Jones, David Jones, Stian Knappskog, Marcel Kool, Sunil R. Lakhani, Carlos Lo´pez-Otı´n, Sancha Martin, Nikhil C. Munshi, Hiromi Nakamura, Paul A. Northcott, Marina Pajic, Elli Papaemmanuil, Angelo Paradiso, John V. Pearson,Xose S. Puente, KeiranRaine, Manasa Ramakrishna, Andrea L. Richardson, Julia Richter, Philip Rosenstiel, Matthias Schlesner, Ton N. Schumacher, Paul N. Span, Jon W. Teague, Yasushi Totoki, Andrew N. J. Tutt, Rafael Valde´s-Mas, Marit M. van Buuren, Laura van 't Veer, Anne Vincent-Salomon, NicolaWaddell, Lucy R. Yates, Australian Pancreatic Cancer Genome Initiative, ICGC Breast Cancer Consortium, ICGC MMML-Seq Consortium, ICGC PedBrain, Jessica Zucman-Rossi, P. Andrew Futreal, Ultan McDermott, Peter Lichter, Matthew Meyerson, Sean M. Grimmond, Reiner Siebert, Elı´as Campo, Tatsuhiro Shibata, Stefan M. Pfister, Peter J. Campbell & Michael R. Stratton. Nature 500, 415–421 (2013).

22. Sarah A. Martin, Christopher J. Lord, and Alan Ashworth. Clin Cancer Res; 16(21); 5107–13. ©2010 AACR.

23. Zachary R. Chalmers, Caitlin F. Connelly, David Fabrizio, Laurie Gay, Siraj M. Ali, Riley Ennis, Alexa Schrock, Brittany Campbell, Adam Shlien, Juliann Chmielecki, Franklin Huang, Yuting He, James Sun, Uri Tabori, Mark Kennedy, Daniel S. Lieber, Steven Roels, Jared White, Geoffrey A. Otto, Jeffrey S. Ross, Levi Garraway, Vincent A. Miller, Phillip J. Stephens and Garrett M. Frampton. Genome Medicine (2017) 9:34.

24. Audrey Petitjean, Ewy Mathe, Shunsuke Kato, Chikashi Ishioka, Sean V. Tavtigian, Pierre Hainaut, and Magali Olivier. HUMAN MUTATION 28(6),622-629, 2007.

25. Jarushka Naidoo and Alexander Drilon. Advances in Experimental Medicine and Biology 893.

26. Peters. *Mutational burden determines sensitivity to PD-1 blockade in NSCLC* .AACR 2017, Oral.

27. M.D. Hellmann, T.-E. Ciuleanu, A. Pluzanski, J.S. Lee, G.A. Otterson, C. Audigier-Valette, E. Minenza, H. Linardou, S. Burgers, P. Salman, H. Borghaei, S.S. Ramalingam, J. Brahmer, M. Reck, K.J. O'Byrne, W.J. Geese, G. Green, H. Chang, J. Szustakowski, P. Bhagavatheeswaran, D. Healey, Y. Fu, F. Nathan, and L. Paz-Ares. This article was published on April 16, 2018, at NEJM.org. DOI: 10.1056/NEJMoa1801946.

28. Decoster L, Wauters I, Vansteenkiste JF. Vaccination therapy for non-small-cell lung cancer: review of agents in phase III development. Ann Oncol 2012; 23: 1387-1393

29. Herbst RS, Soria J-C, Kowanetz M, Fine GD, Hamid O, Gordon MS, et al. Predictive correlates of response to the anti-PD-L1 antibody MPDL3280A in cancer patients. Nature. 2014 Nov 27;515(7528):563–7.

30. Fehrenbacher L, Spira A, Ballinger M, Kowanetz M, Vansteenkiste J, Mazieres J, et al. Atezolizumab versus docetaxel for patients with previously treated non-small-cell lung cancer (POPLAR): a multicentre, open-label, phase 2 randomised controlled trial. Lancet. 2016 Apr 30;387(10030):1837–46.

31. Rittmeyer A, Barlesi F, Waterkamp D, Park K, Ciardiello F, von Pawel J, et al. Atezolizumab versus docetaxel in patients with previously treated non-small-cell lung cancer (OAK): a phase 3, open-label, multicentre randomised controlled trial. Lancet. 2017 Jan 21;389(10066):255–65.

32. Gerber HP, Kowalski J, Sherman D et al. Complete inhibition of rhabdomyosarcoma xenograft growth and neovascularization requires blockade of both tumor and host vascular endothelial growth factor. Cancer Res 2000; 60: 6253-6258.

33. Mordenti J, Thomsen K, Licko V et al. Efficacy and concentration-response of murine anti-VEGF monoclonal antibody in tumor-bearing mice and extrapolation to humans. Toxicol Pathol 1999; 27: 14-21.

34. Aaronson NK, Ahmedzai S, Bergman B et al. The European Organization for Research and Treatment of Cancer QLQ-C30: A quality-of-life instrument for use in international clinical trials in oncology. J Natl Cancer Inst 1993; 85: 365-76.

35. Bergman B, Aaronson NK, Ahmedzai S, et al. The EORTC QLQ-LC13: a modular supplement to the EORTC core quality of life questionnaire (QLQ-C30) for use in lung cancer clinical trials. Eur J Cancer 1994; 30A: 635-42.

36. Frampton GM, et al. Development and validation of a clinical cancer genomic profiling test based on massively parallel DNA sequencing. Nat Biotechnol. 2013 Nov;31(11):1023-31.

37. Meric-Bernstam F, et al. Feasibility of Large-Scale Genomic Testing to Facilitate Enrollment Onto Genomically Matched Clinical Trials. J Clin Oncol. 2015 Sep 1; 33(25): 2753–2762.

38. T. Seto, K. Nosaki, M. Shimokawa, R. Toyozawa, S. Sugawara, H. Hayashi, H. Murakami, T. Kato, S. Niho, H. Saka, M. Oki, H. Yoshioka, I. Okamoto, H. Daga, K. Azuma, H. Tanaka, K. Nishino, M. Satouchi, N. Yamamoto, K. Nakagawa. LBA55. ESMO 2020

20.1. References for translational research

[a] E. Cha, M. Klinger, Y. Hou, C. Cummings, A. Ribas, M. Faham, et al., Improved Survival with T Cell Clonotype Stability After Anti – CTLA-4 Treatment in Cancer Patients, Sci. Transl. Med. 6 (2014) 238ra70. doi:10.1126/scitranslmed.3008211.

[b] N. Akyüz, A. Brandt, A. Stein, S. Schliffke, T. Mährle, J. Quidde, et al., T-cell diversification reflects antigen selection in the blood of patients on immune checkpoint inhibition and may be exploited as liquid biopsy biomarker, Int. J. Cancer. 140 (2017) 2535–2544. doi:10.1002/ijc.30549.

[c] P.M. Forde, J.E. Chaft, K.N. Smith, V. Anagnostou, T.R. Cottrell, M.D. Hellmann, et al., Neoadjuvant PD-1 Blockade in Resectable Lung Cancer, N. Engl. J. Med. (2018) NEJMoa1716078. doi:10.1056/NEJMoa1716078.

[d] Casarrubios, M. *et al.* Pretreatment Tissue TCR Repertoire Evenness Is Associated with Complete Pathologic Response in Patients with NSCLC Receiving Neoadjuvant Chemoimmunotherapy. *Clin. Cancer Res.* **27**, 5878–5890 (2021).

[e] Stankovic, B. *et al.* Immune Cell Composition in Human Non-small Cell Lung Cancer. *Front. Immunol.* **9**, 3101 (2018).

[f] Cabrita, Rita, Martin Lauss, Adriana Sanna, Marco Donia, Mathilde Skaarup Larsen, Shamik Mitra, Iva Johansson, et al. 2020. "Tertiary Lymphoid Structures Improve Immunotherapy and Survival in Melanoma." Nature 577 (7791): 561–65. https://doi.org/10.1038/s41586-019-1914-8.

[g] Helmink BA, Reddy SM, Gao J, Zhang S, Basar R, Thakur R, et al. B cells and tertiary lymphoid structures promote immunotherapy response. Nature. 2020;577:549–55.

[h] Hernandez C, Arasanz H, Chocarro L, Bocanegra A, Zuazo M, Fernandez-Hinojal G, et al. Systemic blood immune cell populations as biomarkers for the outcome of immune checkpoint inhibitor therapies. Int J Mol Sci. 2020;21:1–13.

[i] V. Gopalakrishnan, C.N. Spencer, L. Nezi, A. Reuben, M.C. Andrews, T. V. Karpinets, et al., Gut microbiome modulates response to anti-PD-1 immunotherapy in melanoma patients, Science (80-.). 359 (2018) 97–103. doi:10.1126/science.aan4236.

[j] B. Routy, E. Le Chatelier, L. Derosa, C.P.M. Duong, M.T. Alou, R. Daillère, et al., Gut microbiome influences efficacy of PD-1-based immunotherapy against epithelial tumors, Science (80-.). 359 (2018) 91–97. doi:10.1126/science.aan3706.

[k] Y. Jin, H. Dong, L. Xia, Y. Yang, Y. Zhu, Y. Shen, et al., The Diversity of Gut Microbiome is Associated With Favorable Responses to Anti–Programmed Death 1 Immunotherapy in Chinese Patients With NSCLC, J. Thorac. Oncol. 14 (2019) 1378–1389. doi:10.1016/j.jtho.2019.04.007.

Appendices:

1. Common Terminology criteria for Adverse Events (CTCAE)
2. RECIST criteria v1.1
3. SAE Form
4. Pregnancy Form
5. Health-related Quality of life questionnaire: EORTC C30 (QLQ-C30) and submodule LC13
6. System of classification for non-microcytic lung cancer (NSCLC) and definition of lymph node maps, 8th edition
7. Risks associated with Atezolizumab and guidelines for management of adverse events associated with Atezolizumab
8. Patients pre-screening form TELMA
9. Foundation one CDx Specimen Instructions
10. Foundation one Liquid technical sheet
11. Foundation one CDx technical sheet

APPENDIX 1. Common Terminology criteria for Adverse Events (CTCAE)

Version 5.0: November 27, 2017 is available from the internet at:

https://ctep.cancer.gov/protocolDevelopment/electronic_applications/docs/CTCAE_v5_Quick_Reference_8.5x11.pdf

APPENDIX 2. RECIST criteria v1.1

1. Introduction

All included patients will be evaluated for response according to the revised Response Evaluation Criteria In Solid Tumors (RECIST version 1.1) [1]. This appendix defines all criteria applied in this trial.

2. Methods of assessment

The same method of assessment and the same technique should be used to characterize each identified and reported lesion at baseline and during follow-up. Imaging-based evaluation is preferred to evaluation by clinical examination when both methods have been used to assess the antitumor effect of a treatment.

CT is the best currently available and reproducible method to measure lesions selected for response assessment. CT should generally be performed using a ≤ 5 mm contiguous reconstruction algorithm. **MRI** is acceptable for certain situations, e.g. body scans.

Clinical lesions will only be considered measurable when they are superficial (e.g. skin nodules) and ≥10 mm. In the case of skin lesions, documentation by color photography including a ruler to estimate the size of the lesion is recommended.

Lesions on **chest X-ray** are acceptable as measurable lesions when they are clearly defined and surrounded by aerated lung. However, CT is preferable.

Ultrasound is not useful in assessment of lesion size and is not accepted as method of assessment.

FDG-PET is not foreseen for regular response assessments. It may, however, be used to detect or confirm the appearance of new lesions. Attenuation correction CT scans performed as part of a **PET/CT** scan frequently show lower resolution; therefore, dedicated CT scans are preferred. However, if the site can demonstrate that the CT performed as part of a PET/CT is of the same diagnostic quality as a diagnostic CT (with IV and oral contrast), then the CT portion of the PET/CT can be used for RECIST measurements.

3. Measurability of tumor at baseline

Measurable disease is defined as the presence of at least one measurable lesion.

Measurable lesions:

- **Tumor lesions** must be accurately measured in at least one dimension (longest diameter in the plane of measurement is to be recorded) with a minimum size of
  - 10 mm by CT scan (CT scan slice thickness no greater than 5mm)

- 10mm caliper measurement by clinical exam (lesions which cannot be accurately measured with calipers should be recorded as non-measurable)
- 20 mm by chest X-ray

- **Malignant lymph nodes:** to be considered pathologically enlarged and measurable, a lymph node must be ≥ 15mm in *short* axis when assessed by CT scan, assuming the slice thickness is ≤ 5 mm. At baseline and in follow-up, only the *short* axis will be measured.

Non-measurable lesions: all other lesions, i.e.:

- Small non-nodal lesions (longest diameter < 10 mm in CT scan)
- Small lymph nodes (short axis ≥ 10 and < 15 mm). Lymph nodes that have a short axis < 10 mm are considered non-pathological and should not be recorded or followedBone lesions. Lytic bone lesions or mixed lytic-blastic lesions, with identifiable soft tissue components, can be considered as measurable lesions if the soft tissue component meets the definition of measurability described above. Blastic bone lesions are non-measurable.
- Leptomeningeal disease
- Ascites
- Pleural or pericardial effusion
- Inflammatory breast disease
- Lymphangitic involvement of skin or lung
- Cystic lesions. Cystic lesions thought to represent cystic metastases may be considered as measurable lesions. However, if non-cystic lesions are present, these are preferred as target lesions
- Tumor lesions situated in a previously irradiated area, or subjected to other locoregional therapy. Such lesions may be considered measurable if there has been demonstrated progression in the lesion
- abdominal masses/abdominal organomegaly identified by physical exam that are not measurable by reproducible imaging techniques

4. Selection of lesions

4.1. Selection of target lesions

At baseline, measurable lesions up to a maximum of 5 lesions representative of all involved organs, and up to 2 per organ, should be identified as target lesions and measured and recorded. Target lesions (TL) should be selected on the basis of their size and their suitability for accurate repetitive measurements. A sum of diameters for all target lesions will be calculated and reported as the baseline sum of diameters. Lymph nodes selected as TL should always have the short axis recorded. All other lesions should always have their longest diameters recorded. The sum of diameters will be used as reference to further characterize the objective tumor response of the measurable dimension of the disease.

4.2. Selection of non-target lesions

All other lesions (or sites of disease) should be identified as non-target lesions and should also be recorded at baseline. Measurements are not required, but the presence or absence of each should be noted throughout follow-up. It is possible to record multiple non-target lesions as a single item on the CRF (e.g. "multiple liver metastases").

5. Evaluation of Lesions

5.1. Evaluation of Target Lesions

All target lesions will be measured at each tumor assessment, and the sum of their diameters will be compared to previous assessments in order to assign the response status as specified below.

- Complete Response (CR): Disappearance of all target lesions. Lymph nodes selected as target lesions must each have reduction in the short axis to < 10 mm in order for the response to be considered complete. In this case, the sum of diameters may be > 0.
- Partial Response (PR): At least a 30% decrease in the sum of diameters of target lesions taking as reference the baseline sum of diameters.
- Progression (PD): At least a 20% increase in the sum of diameters of target lesions, taking as reference the smallest sum recorded on study. In addition to the relative increase of 20%, the sum must also demonstrate an absolute increase of at least 5 mm.
- Stable Disease (SD): Neither sufficient shrinkage to qualify for PR nor sufficient increase to qualify for PD taking as reference the smallest sum of diameters recorded on study.
- Inevaluable for response: specify reasons (for example: early death due to malignant disease or toxicity; tumor assessment not repeated/incomplete; other, specify).

Note: All target lesions, including lymph nodes, should have their actual measurements recorded at each subsequent evaluation, even when very small (e.g. 2 mm). If the radiologist does not feel comfortable assigning an exact measure and reports a lesion as "too small to measure", a default value of 5 mm should be recorded. If a TL is thought likely to have disappeared, "0 mm" is noted.

5.2. Evaluation of Non-Target Lesions

- Complete Response (CR): Disappearance of all non-target lesions; lymph nodes selected as non-target lesions must be non-pathological in size (< 10 mm).
- Non-CR/non-PD: Persistence of one or more non-target lesions (non-CR).
- Progression (PD): unequivocal progression of existing non-target lesions. Unequivocal means: comparable in magnitude to the increase that would be required to declare PD for measurable disease, or an overall substantial increase in tumor burden that merits treatment discontinuation.

5.3. Determination of new lesions

The appearance of any new malignant lesions denotes disease progression. The finding of a new lesion should be unequivocal, i.e. not attributable to differences in scanning technique or findings thought to represent something other than tumor. If a new lesion is equivocal, e.g. because of its small size, the patient will stay on treatment (if the decision on PD is based on this lesion only). If the repeat scans confirm there is definitely a new lesion, then progression should be declared using the date of the previous scan.

Lesions found in a new location not included in the baseline scan (e.g. brain metastases) are considered new lesions.

Note: the "re-appearance" of a previously "disappeared" target or non-target lesion does not in itself necessarily qualify as PD; this is the case only if the overall evaluation meets the PD criteria, or if the patient was previously in CR.

5.4. Additional considerations

In some circumstances, it may be difficult to distinguish residual disease from normal tissue. When the evaluation of complete response depends upon this determination, it is recommended that the residual lesion be investigated (fine needle aspirate/biopsy) before confirming the complete response status.

The cytological confirmation of the neoplastic origin of any effusion that appears or worsens during treatment when the measurable tumor has met criteria for response or stable disease is mandatory to differentiate between response or stable disease (an effusion may be a side effect of the treatment) and progressive disease.

6. Determination of time point response

Based on the responses of Target Lesions, Non-Target Lesions, and the presence or absence of new lesions, the overall response will be determined at each tumor assessment time point, according to the table below:

Target lesions	Non-target lesions	New lesions	Overall response
CR	CR	No	CR
CR	Non-CR / non-PD	No	PR
CR	Not evaluated	No	PR
PR	Non-PD or not all evaluated	No	PR

SD	Non-PD or not all evaluated	No	SD
Not all evaluated	Non-PD	No	NE (unless the sum of diam. Of evaluated lesions indicates PD[1])
PD	Any	Yes or no	PD
Any	PD	Yes or no	PD
Any	Any	Yes	PD

[1] From ref. 1 p.234: When no imaging/measurement is done at all at a particular time point, the patient is not evaluable (NE) at that time point. If only a subset of lesion measurements are made at an assessment, usually the case is also considered NE at that time point, unless a convincing argument can be made that the contribution of the individual missing lesion(s) would not change the assigned time point response. This would be most likely to happen in the case of PD. For example, if a patient had a baseline sum of 50mm with three measured lesions and at follow-up only two lesions were assessed, but those gave a sum of 80 mm, the patient will have achieved PD status, regardless of the contribution of the missing lesion.

7. Determination of best overall response

Best overall response is defined as best response across all time points. Confirmation of partial or complete response by an additional scan is not requested in this trial. Best overall response will be determined by central review.

8. References

1. Eisenhauer EA, Therasse P, Bogaerts J, Schwartz LH, Sargent D, Ford R, Dancey J, Arbuck S, Gwyther S, Mooney M, Rubinstein L, Shankar L, Dodd L, Kaplan R, Lacombe D, Verweij J. New response evaluation criteria in solid tumours: revised RECIST guideline (version 1.1). EurJ Cancer 2009;45(2):228-47.

APPENDIX 3. SAE Form

SERIOUS ADVERSE EVENTS NOTIFICATION FORM (SAE)	PROTOCOL Code: GECP 18/03 (TELMA)<br>Roche code: ML40237	
	Site:	
TYPE OF NOTIFICATION:<br>☐ INITIAL ☐ FOLLOW UP nº..........	Investigator:	SAE Form Nr:

SERIOUS ADVERSE EVENT INFORMATION

PATIENT CODE	DATE OF BIRTH	AGE	GENDER<br>☐ Male ☐ Female	WEIGHT (kg)	HEIGHT (cm)

DATE OF SAE AWARENESS: I_____I______I______I	SAE NOTIFICATION DATE: I_____I_____I______I

SERIOUS ADVERSE EVENT (please, fill in the following field with the information related to the serious adverse events. The first one has to be the more relevant. The SAE term has to be filled in with the verbatim term. The SAE term according to the CTCAE will be coded by the Fundación GECP pharmacovigilance department in the study data base.

SAE term	Grade (1-5)	Start Date/Stop date	Causality[1]	Key for seriousness[2]	Outcome[3]
		Start D.: I____I____I____I<br>Stop D.: I____I____I___I	 *		 Outcome date: I____I____I____I
		Start D.: I____I____I____I<br>Stop D.: I____I____I___I	 *		
		Start D.: I____I____I____I<br>Stop D.: I____I____I___I	 *		
		Start D.: I____I____I____I<br>Stop D.: I____I____I___I	 *		
		Start D.: I____I____I____I<br>Stop D.: I____I____I___I	 *		

[1] Causality (relation to study drugs):<br>*0: Not related; 1: Related*; 2: Not applicable*<br>*Specify drug and/or therapy (e.g Radiotherapy)	[2] Key for Seriousness:<br>*1: Death; 2: Life threatening; 3: Initial/Prolonged hospitalization admission; 4:Congenital Anomaly/Birth Defect;5: Persistent or significant Disability ; 6: Medically significant (important medical event) ; 7: Non-serious adverse event of special interest (AESI) as per protocol; 8: Non-serious secondary malignancy as per protocol*	[3] Outcome<br>*1: Resolved without sequelae ; 2: Resolved with sequelae; 3: Fatal; 4: Ongoing 5: Unknown*

**SAE COMMENTS** (Please, point out any relevant data related to the SAE, test results, any additional relevant information, in case of death, please add information about date of death, cause and autopsy if applicable)

SAE Form_v.5.0_16Abr2019 1 de 2

PATIENT CODE	PROTOCOL CODE	SAE FORM Nº
	GECP 18/03 (TELMA)_Roche code: ML40237	

INFORMATION ABOUT INVESTIGATIONAL MEDICAL PRODUCT

Trial Medication (Generic name/Brand name)	Dose and units	Route (IV, PO, SC...)	Date of administration before SAE	Action taken[4]	Stopped the event when the medication was discontinued?[5]	The event returned when the medication is resumed?[5]

¿IS THERE ANY OTHER EXPLANATION OR CAUSE FOR THE ADVERSE EVENT?

☐ CONCOMITANT DISEASE ☐ POSSIBLE RELATION WITH CONCOMITANT DRUGS ☐ STUDY PROCEDURES ☐ NOT APPLICABLE ☐ OTHERS (SPECIFY):

[4] Action taken with regard to trial medication: *0: none; 1: Reduced; 2: Delayed; 3:Delayed and Reduced; 4: permanent discontinuation; 5: other (specify):*____

[5] Stopped or returned the adverse event when the medication was discontinued or resumed? *0: YES; 1: NO; 2: Not applicable*

PREVIOUS RELEVANT MEDICAL HISTORY

PREVIOUS RELEVANT MEDICAL HISTORY (PREVIOUS DIAGNOSIS, SURGERIES, ALLERGY, PREGNANCIES, CONCOMITANT DISEASES, ETC):

CONCOMITANT MEDICATION *(Medication took by the patient due to basal diseases or supportive Clinical trial medication)*

CONCOMITANT MEDICATION (Brand name)	Daily Dose	Route (IV, PO, SC...)	Onset Date	Ending date or Ongoing?	Prescription reason[6]

[6] Prescription reason: 1: Regular medication/baseline conditions; 2: Concomitant Medication/prophylaxis; 3: Adverse event; 4: Other (specify)

INFORMATION ABOUT THE SPONSOR AND THE PRINCIPAL INVESTIGATOR (PI) OF THE TRIAL

NAME AND CONTACT DETAILS OF THE SPONSOR	DATE RECEIVED AND SIGNATURE OF THE SPONSOR:	NAME AND CONTACT DETAILS OF THE PI:	DATE AND SIGNATURE OF THE PI:
Fundación GECP<br>*Av. Meridiana, 358 6º pl*<br>*08027 Barcelona*<br>*Tel: (34) 93 430 20 06*<br>*Fax: (34) 93 419 17 68*	|_____|_____|_______|		|_____|_____|_______|

PLEASE FAX THIS FORM TO THE Fundación GECP (SLCG/GECP): + 34 93 419 17 68 or EMAIL IT TO:
pharmacovigilance.slcg@gecp.org

SAE Form_v.5.0_16Abr2019

2 de 2

APPENDIX 4. Pregnancy Form

PREGNANCY NOTIFICATION FORM<br>GeCP lung cancer research	PROTOCOL Code: GECP 18/03 (TELMA)<br>Roche code: ML40237	
	Site:	
TYPE OF NOTIFICATION:<br>□ INITIAL □ FOLLOW UP nº.........	Investigator:	PREGNANCY Form Nr:

PATIENT CODE	DATE OF BIRTH	AGE	GENDER<br>□ Male □ Female	WEIGHT (kg)	HEIGHT (cm)
DATE OF PREGNANCY AWARENESS: I_____I______I______I			PREGNANCY NOTIF. DATE: I_____I_____I_____I		

PREGNANCY REPORT INFORMATION

The pregnancy has occurred in: □ Clinical trial subject * □ Female partner of trial subject
*If in pregnant trial subject. Was she withdrawn consent from study? □ YES** □ NO
** Date of withdrawal of the study I_______I________I_________I
State of the pregnancy at the moment of the withdrawal: □ Suspected □ Positive pregnancy test □ Confirmed

PHASE OF THE TREATMENT WHERE PREGNANCY WAS DETECTED AND TYPE OF CLINICAL TRIAL

□ Screening Phase □ Randomized or included patient* □ During Follow up Phase □ NA (the study not started yet)
Is it a blind Clinical trial? □ NO □ YES; If yes, Was the blind broken? □ NO □ YES*
*Specify (treatment arm, treatment...):____

MATERNAL INFORMATION

Mother date of birth: I_____I______I_______I	Contraception information:
Date of last menstrual period: I_____I______I_______I	Was the mother/father using a method of contraception?<br>□ Yes* □ No<br>*Specify: □ Oral contraception □ Condom<br>□ Diaphragm □ Others (specify): _______________
Estimated delivery date: I_____I______I_______I	
Father date of birth: I_____I______I_______I	
First pregnancy awareness date: I_____I______I_______I	Has the mother any previous pregnancies?<br>□ YES (complete next section) □ NO
PI awareness date of the pregnancy: I_____I______I_______I	

PREGNANCY RELEVANT MEDICAL HISTORY

Number of full-term pregnancies: I______I Number of pre-term pregnancies: I______I

Previous pregnancies outcome:
Normal births (nº): I_____I; Children born with defects: I______I; Spontaneous abortion: I_______I;
Induced abortion: I______I; Ectopic pregnancy: I______I; Others I______I (specify): ________________________

Are any risk factors to the current pregnancy outcome? (e.g: alcohol or substance abuse, chronic diseases, family history of birth defects...), specify: ____
____

Pregnancy Notification Form_v.3.0_26Abr2019 1 de 3

PATIENT CODE	PROTOCOL CODE<br>GECP 18/03 (TELMA)_Roche code: ML40237	PREGNANCY Form Nr

PREVIOUS RELEVANT MEDICAL HISTORY FROM MOTHER AND/OR FATHER

PREVIOUS RELEVANT MEDICAL HISTORY (PREVIOUS DIAGNOSIS, SURGERIES, ALLERGY, CONCOMITANT DISEASES, ETC):

INFORMATION ABOUT INVESTIGATIONAL MEDICAL PRODUCT

Trial medication (Generic name/Brand name)	Time of exposure[1]	Daily dose	Route (IV, PO, SC...)	Start Date	Stop date or Ongoing?	Action taken with regard to trial medication[2]

[1]Time of exposure: 0: pre-conception; 1: 1st trimester; 2: 2nd trimester; 3: 3rd trimester

[2] Action taken with regard to trial medication: 0: None; 1: Reduced; 2: Delayed; 3: Delayed and reduced; 4: permanent discontinuation; 5: other (specify): ____

CONCOMITANT MEDICATION *(medication took by the patient due to basal diseases or supportive clinical trial medication)*

CONCOMITANT MEDICATION (Brand name)	Daily dose	Route (IV, PO, SC...)	Onset Date	Ending date or Ongoing?	Prescription reason[3]

[3] Prescription reason: 1: Regular medication/baseline conditions; 2: Concomitant medication/prophylaxis; 3: Adverse Event; 4: other (specify)

CURRENT PREGNANCY STATUS AND OUTCOME

ONGOING ◻ Yes ◻ No (complete section) ◻ Unknown<br>◻ Lost to follow up: I_____I_____I_______I	Newborn birth date: I_____I______I______I<br>(delivery date)
Induced abortion date: I_____I______I_______I	Delivery mode: ◻ vaginal ◻ caesarian<br>◻ Unknown/others:
Spontaneous abortion date: I_____I______I_______I	Gestational weeks at birth: I______I
Ectopic pregnancy date: I_____I______I_______I	Duration of delivery: ______ hours _____ minutes
Others date: I_____I______I_______I	Has any prenatal testing been performed?<br>◻ YES (attach anonymized report) ◻ NO
PI date of awareness: I_____I______I_______I	

PATIENT CODE	PROTOCOL CODE	PREGNANCY Form Nº
	GECP 18/03 (TELMA)_Roche code: ML40237	

NEWBORN BABY INFORMATION

(If multiple births complete this section for each enfant)

Number of babies: I______I	Gender: ◻ Male ◻ Female		
Apgar 1 min_______ 5 min_______10 min________	Weight (Kg):	Height (cm):	Head circumference (cm):
If any abnormality in the newborn appeared, then answer the following question:<br>Are there any hints that these incidences may be related to the drug exposure of the mother/father during pregnancy?<br>◻ YES (complete SAE Form)<br>◻ NO	General status of the newborn baby (specify):		
Other relevant information about the delivery/ newborn:			

INFORMATION ABOUT THE STAFF WHO ATTENDED THE DELIVERY

GYNECOLOGIST:	PEDIATRICIAN:	OTHERS:

INFORMATION ABOUT THE SPONSOR AND THE PRINCIPAL INVESTIGATOR (PI) OF THE TRIAL

NAME AND CONTACT DETAILS OF THE SPONSOR	DATE RECEIVED AND SIGNATURE OF THE SPONSOR:	NAME AND CONTACT DETAILS OF THE PI:	DATE AND SIGNATURE OF THE PI:
Fundación GECP<br>*Av. Meridiana, 358 6º pl*<br>*08027 Barcelona*<br>*Tel: (34) 93 430 20 06*<br>*Fax: (34) 93 419 17 68*	I______I______I________I		I______I______I________I
PLEASE FAX THIS FORM TO THE Fundación GECP (SLCG/GECP): + 34 93 419 17 68 or EMAIL IT TO: *pharmacovigilance.slcg@gecp.org*			

Pregnancy Notification Form_v.3.0_26Abr2019

3 de 3

APPENDIX 5. Health-related Quality of life questionnaire: EORTC C30 (QLQ-C30) and submodule LC13

Please see documents attached to the protocol

APPENDIX 6. System of classification for non-microcytic lung cancer (NSCLC) and definition of lymph node maps, 8th edition

T: Primary tumor	
Tx	Primary tumor cannot be assessed or tumor proven by presence of malignant cells in sputum or bronchial washings but not visualized by imaging or bronchoscopy
T0	No evidence of primary tumor
Tis	Carcinoma in situ
T1	Tumor ≤3 cm in greatest dimension surrounded by lung or visceral pleura without bronchoscopic evidence of invasion more proximal than the lobar bronchus (i.e., not in the main bronchus)[a]
**T1a(mi)**	**Minimally invasive adenocarcinoma**[b]
**T1a**	**Tumor ≤1 cm in greatest dimension**[a]
**T1b**	**Tumor >1 cm but ≤2 cm in greatest dimension**[a]
**T1c**	**Tumor >2 cm but ≤3 cm in greatest dimension**[a]
**T2**	Tumor >3 cm **but ≤5 cm** or tumor with any of the following features[c]:<br>- **Involves main bronchus regardless of distance from the carina but without involvement of the carina**<br>- Invades visceral pleura<br>- **Associated with atelectasis or obstructive pneumonitis that extends to the hilar region, involving part or all of the lung**
**T2a**	**Tumor >3 cm but ≤4 cm in greatest dimension**
**T2b**	**Tumor >4 cm but ≤5 cm in greatest dimension**
**T3**	**Tumor >5 cm but ≤7 cm in greatest dimension** or associated with separate tumor nodule(s) in the same lobe as the primary tumor or directly invades any of the following structures: chest wall (including the parietal pleura and superior sulcus tumors), phrenic nerve, parietal pericardium
**T4**	**Tumor >7 cm in greatest dimension** or associated with separate tumor nodule(s) in a different ipsilateral lobe than that of the primary tumor or invades any of the following structures: **diaphragm**, mediastinum, heart, great vessels, trachea, recurrent laryngeal nerve, esophagus, vertebral body, and carina
N: Regional lymph node involvement	
Nx	Regional lymph nodes cannot be assessed
N0	No regional lymph node metastasis
N1	Metastasis in ipsilateral peribronchial and/or ipsilateral hilar lymph nodes and intrapulmonary nodes, including involvement by direct extension
N2	Metastasis in ipsilateral mediastinal and/or subcarinal lymph node(s)
N3	Metastasis in contralateral mediastinal, contralateral hilar, ipsilateral or contralateral scalene, or supraclavicular lymph node(s)
M: Distant metastasis	
M0	No distant metastasis
M1	Distant metastasis present
M1a	Separate tumor nodule(s) in a contralateral lobe; tumor with pleural or pericardial nodule(s) or malignant pleural or pericardial effusion[d]
**M1b**	**Single extrathoracic metastasis**[e]
**M1c**	**Multiple extrathoracic metastases in one or more organs**

Note: Changes to the seventh edition are in bold.
[a]The uncommon superficial spreading tumor of any size with its invasive component limited to the bronchial wall, which may extend proximal to the main bronchus, is also classified as T1a.
[b]Solitary adenocarcinoma, ≤ 3cm with a predominately lepidic pattern and ≤ 5mm invasion in any one focus.
[c]T2 tumors with these features are classified as T2a if ≤4 cm in greatest dimension or if size cannot be determined, and T2b if >4 cm but ≤5 cm in greatest dimension.
[d]Most pleural (pericardial) effusions with lung cancer are due to tumor. In a few patients, however, multiple microscopic examinations of pleural (pericardial) fluid are negative for tumor and the fluid is nonbloody and not an exudate. When these elements and clinical judgment dictate that the effusion is not related to the tumor, the effusion should be excluded as a staging descriptor.
[e]This includes involvement of a single distant (nonregional) lymph node.

Occult carcinoma	TX	N0	M0
Stage 0	Tis	N0	M0
**Stage IA1**	**T1(mi)**	**N0**	**M0**
	**T1a**	**N0**	**M0**
**Stage IA2**	**T1b**	**N0**	**M0**
**Stage IA3**	**T1c**	**N0**	**M0**
Stage IB	T2a	N0	M0
Stage IIA	T2b	N0	M0
Stage IIB	**T1a-c**	**N1**	**M0**
	**T2a**	**N1**	**M0**
	T2b	N1	M0
	T3	N0	M0
Stage IIIA	**T1a-c**	**N2**	**M0**
	T2a-b	N2	M0
	T3	N1	M0
	T4	N0	M0
	T4	N1	M0
Stage IIIB	**T1a-c**	**N3**	**M0**
	T2a-b	N3	M0
	**T3**	**N2**	**M0**
	T4	N2	M0
**Stage IIIC**	**T3**	**N3**	**M0**
	**T4**	**N3**	**M0**
**Stage IVA**	**Any T**	**Any N**	**M1a**
	**Any T**	**Any N**	**M1b**
**Stage IVB**	**Any T**	**Any N**	**M1c**

APPENDIX 7. Risks associated with Atezolizumab and guidelines for management of adverse events associated with Atezolizumab

Toxicities associated or possibly associated with atezolizumab treatment should be managed according to standard medical practice. Additional tests, such as autoimmune serology or biopsies, should be used to evaluate for a possible immunogenic etiology, when clinically indicated.

Although most immune-mediated adverse events observed with immunomodulatory agents have been mild and self-limiting, such events should be recognized early and treated promptly to avoid potential major complications. Discontinuation of atezolizumab may not have an immediate therapeutic effect, and in severe cases, immune-mediated toxicities may require acute management with topical corticosteroids, systemic corticosteroids, or other immunosuppressive agents.

The investigator should consider the benefit-risk balance a given patient may be experiencing prior to further administration of atezolizumab. In patients who have met the criteria for permanent discontinuation, resumption of atezolizumab may be considered if the patient is deriving benefit and has fully recovered from the immune-mediated event. The decision to rechallenge patients with atezolizumab should be based on investigator’s assessment of benefit-risk and documented by the investigator (or an appropriate delegate). Medical Monitor is available to advise as needed.

Guidelines for managing patients who experience selected adverse events are provided in the following sections. Management guidelines are presented by adverse event severity based on the National Cancer Institute Common Terminology Criteria for Adverse Events (NCI CTCAE).

PULMONARY EVENTS

Dyspnea, cough, fatigue, hypoxia, pneumonitis, and pulmonary infiltrates have been associated with the administration of atezolizumab. Patients will be assessed for pulmonary signs and symptoms throughout the study and will have computed tomography (CT) scans of the chest performed at every tumor assessment.

All pulmonary events should be thoroughly evaluated for other commonly reported etiologies such as pneumonia or other infection, lymphangitic carcinomatosis, pulmonary embolism, heart failure, chronic obstructive pulmonary disease, or pulmonary hypertension. Management guidelines for pulmonary events are provided in Table 1.

Table 1 Management Guidelines for Pulmonary Events, Including Pneumonitis

Event	Management
Pulmonary event, Grade 1	• Continue atezolizumab and monitor closely.<br>• Re-evaluate on serial imaging.<br>• Consider patient referral to pulmonary specialist.<br>• For Grade 1 pneumonitis, consider withholding atezolizumab
Pulmonary event, Grade 2	• Withhold atezolizumab for up to 12 weeks after event onset. [a]<br>• Refer patient to pulmonary and infectious disease specialists and consider bronchoscopy or BAL.<br>• Initiate treatment with corticosteroids equivalent to 1–2 mg/kg/day oral prednisone.<br>• If event resolves to Grade 1 or better, resume atezolizumab. [b]<br>• If event does not resolve to Grade 1 or better while withholding atezolizumab, permanently discontinue atezolizumab and contact Medical Monitor. [c]<br>• For recurrent events or events with no improvement after 48–72 hours of corticosteroids, treat as a Grade 3 or 4 event.
Pulmonary event, Grade 3 or 4	• Permanently discontinue atezolizumab and contact Medical Monitor. [c]<br>• Bronchoscopy or BAL is recommended.<br>• Initiate treatment with corticosteroids equivalent to 1–2 mg/kg/day IV methylprednisolone.<br>• If event does not improve within 48 hours after initiating corticosteroids, consider adding an immunosuppressive agent.<br>• If event resolves to Grade 1 or better, taper corticosteroids over ≥1 month.

BAL = bronchoscopic alveolar lavage.

[a] Atezolizumab may be withheld for a longer period of time (i.e., > 12 weeks after event onset) to allow for corticosteroids (if initiated) to be reduced to the equivalent of ≤ 10 mg/day oral prednisone. The acceptable length of the extended period of time must be based on assessment of benefit-risk by the investigator and in alignment with the protocol requirement for duration of treatment and documented by the investigator. Medical Monitor is available to advise as needed.

[b] If corticosteroids have been initiated, they must be tapered over ≥ 1 month to the equivalent of ≤ 10 mg/day oral prednisone before atezolizumab can be resumed.

[c] Resumption of atezolizumab may be considered in patients who are deriving benefit and have fully recovered from the immune-mediated event. The decision to rechallenge patients with atezolizumab should be based on investigator's assessment of benefit-risk and documented by the investigator (or an appropriate delegate). Medical Monitor is available to advise as needed.

HEPATIC EVENTS

Immune-mediated hepatitis has been associated with the administration of atezolizumab. Eligible patients must have adequate liver function, as manifested by measurements of total bilirubin and hepatic transaminases, and liver function will be monitored throughout study treatment. Management guidelines for hepatic events are provided in Table 2.

Patients with right upper-quadrant abdominal pain and/or unexplained nausea or vomiting should have liver function tests (LFTs) performed immediately and reviewed before administration of the next dose of study drug.

For patients with elevated LFTs, concurrent medication, viral hepatitis, and toxic or neoplastic etiologies should be considered and addressed, as appropriate.

Table 2 Management Guidelines for Hepatic Events

Event	Management
**In patients without HCC**	
Hepatic event, Grade 1	• Continue atezolizumab.<br>• Monitor LFTs until values resolve to within normal limits or to baseline values.
Hepatic event, Grade 2	**All events:**<br>• Monitor LFTs more frequently until return to baseline values.<br><br>**Events of > 5 days' duration:**<br>• Withhold atezolizumab for up to 12 weeks after event onset. [a]<br>• Initiate treatment with corticosteroids equivalent to 1–2 mg/kg/day oral prednisone.<br>• If event resolves to Grade 1 or better, resume atezolizumab. [b]<br>• If event does not resolve to Grade 1 or better while withholding atezolizumab, permanently discontinue atezolizumab and contact Medical Monitor. [c]
Hepatic event, Grade 3 or 4	• Permanently discontinue atezolizumab and contact Medical Monitor. [c]<br>• Consider patient referral to GI specialist for evaluation and liver biopsy to establish etiology of hepatic injury.<br>• Initiate treatment with corticosteroids equivalent to 1–2 mg/kg/day oral prednisone.<br>• If event does not improve within 48 hours after initiating corticosteroids, consider adding an immunosuppressive agent.<br>• If event resolves to Grade 1 or better, taper corticosteroids over ≥ 1 month.

Event	Management
**In patients with HCC**	
AST/ALT is within normal limits at baseline and increases to $>3\times$ULN to $\leq 10\times$ULN<br><br>or<br><br>AST/ALT is $>$ULN to $\leq 3\times$ULN at baseline and increases to $>5\times$ULN to $\leq 10\times$ULN<br><br>or<br><br>AST/ALT is $>3\times$ULN to $\leq 5\times$ULN at baseline and increases to $>8\times$ULN to $\leq 10\times$ULN	• Monitor LFTs more frequently until return to baseline values.<br>• Withhold atezolizumab for up to 12 weeks after event onset. [a]<br><br>Events of >5 days' duration:<br>• Consider initiating treatment with corticosteroids equivalent to 1–2 mg/kg/day oral prednisone.<br>• If event resolves to baseline or to Grade 1 or better, resume atezolizumab. [b]<br>• If event does not resolve to baseline or to Grade 1 or better while withholding atezolizumab, permanently discontinue atezolizumab and contact Medical Monitor. [c]
AST or ALT increases to $>10\times$ULN or total bilirubin increases to $>3\times$ULN	• Permanently discontinue atezolizumab and contact Medical Monitor. [c]<br>• Consider patient referral to GI specialist for evaluation and liver biopsy to establish etiology of hepatic injury.<br>• Initiate treatment with corticosteroids equivalent to 1–2 mg/kg/day oral prednisone.<br>• If event does not improve within 48 hours after initiating corticosteroids, consider adding an immunosuppressive agent.<br>• If event resolves to baseline, taper corticosteroids over ≥ 1 month.

GI=gastrointestinal; LFT = liver function test; ULN=upper limit of normal.

[a] Atezolizumab may be withheld for a longer period of time (i.e., >12 weeks after event onset) to allow for corticosteroids (if initiated) to be reduced to the equivalent of ≤ 10 mg/day oral prednisone. The acceptable length of the extended period of time must be based on assessment of benefit-risk by the investigator and in alignment with the protocol requirement for duration of treatment and documented by the investigator. Medical Monitor is available to advise as needed.

[b] If corticosteroids have been initiated, they must be tapered over ≥ 1 month to the equivalent of ≤ 10 mg/day oral prednisone before atezolizumab can be resumed.

[c] Resumption of atezolizumab may be considered in patients who are deriving benefit and have fully recovered from the immune-mediated event. The decision to rechallenge patients with atezolizumab should be based on investigator's assessment of benefit-risk and documented by the investigator (or an appropriate delegate). Medical Monitor is available to advise as needed.

GASTROINTESTINAL EVENTS

Immune-mediated colitis has been associated with the administration of atezolizumab. Management guidelines for diarrhea or colitis are provided in Table 3.

All events of diarrhea or colitis should be thoroughly evaluated for other more common etiologies. For events of significant duration or magnitude or associated with signs of systemic inflammation or acute-phase reactants (e.g., increased C-reactive protein, platelet count, or bandemia): Perform sigmoidoscopy (or colonoscopy, if appropriate) with colonic biopsy, with three to five specimens for standard paraffin block to check for inflammation and lymphocytic infiltrates to confirm colitis diagnosis.

Table 3 Management Guidelines for Gastrointestinal Events (Diarrhea or Colitis)

Event	Management
Diarrhea or colitis, Grade 1	• Continue atezolizumab.<br>• Initiate symptomatic treatment.<br>• Endoscopy is recommended if symptoms persist for >7 days.<br>• Monitor closely.
Diarrhea or colitis, Grade 2	• Withhold atezolizumab for up to 12 weeks after event onset. [a]<br>• Initiate symptomatic treatment.<br>• Patient referral to GI specialist is recommended.<br>• For recurrent events or events that persist >5 days, initiate treatment with corticosteroids equivalent to 1–2 mg/kg/day oral prednisone.<br>• If event resolves to Grade 1 or better, resume atezolizumab. [b]<br>• If event does not resolve to Grade 1 or better while withholding atezolizumab, permanently discontinue atezolizumab and contact Medical Monitor. [c]
Diarrhea or colitis, Grade 3	• Withhold atezolizumab for up to 12 weeks after event onset. [a]<br>• Refer patient to GI specialist for evaluation and confirmatory biopsy.<br>• Initiate treatment with corticosteroids equivalent to 1–2 mg/kg/day IV methylprednisolone and convert to 1–2 mg/kg/day oral prednisone or equivalent upon improvement.<br>• If event resolves to Grade 1 or better, resume atezolizumab. [b]<br>• If event does not resolve to Grade 1 or better while withholding atezolizumab, permanently discontinue atezolizumab and contact Medical Monitor. [c]

Event	Management
Diarrhea or colitis, Grade 4	• Permanently discontinue atezolizumab and contact Medical Monitor. [c]<br>• Refer patient to GI specialist for evaluation and confirmatory biopsy.<br>• Initiate treatment with corticosteroids equivalent to 1–2 mg/kg/day IV methylprednisolone and convert to 1–2 mg/kg/day oral prednisone or equivalent upon improvement.<br>• If event does not improve within 48 hours after initiating corticosteroids, consider adding an immunosuppressive agent.<br>• If event resolves to Grade 1 or better, taper corticosteroids over ≥1 month.

[a] Atezolizumab may be withheld for a longer period of time (i.e., >12 weeks after event onset) to allow for corticosteroids (if initiated) to be reduced to the equivalent of ≤10 mg/day oral prednisone. The acceptable length of the extended period of time must be based on assessment of benefit-risk by the investigator and in alignment with the protocol requirement for duration of treatment and documented by the investigator. Medical Monitor is available to advise as needed.

[b] If corticosteroids have been initiated, they must be tapered over ≥1 month to the equivalent of ≤10 mg/day oral prednisone before atezolizumab can be resumed.

[c] Resumption of atezolizumab may be considered in patients who are deriving benefit and have fully recovered from the immune-mediated event. The decision to rechallenge patients with atezolizumab should be based on investigator's assessment of benefit-risk and documented by the investigator (or an appropriate delegate). Medical Monitor is available to advise as needed.

ENDOCRINE EVENTS

Thyroid disorders, adrenal insufficiency, diabetes mellitus, and pituitary disorders have been associated with the administration of atezolizumab. Management guidelines for endocrine events are provided in Table 4.

Patients with unexplained symptoms such as headache, fatigue, myalgias, impotence, constipation, or mental status changes should be investigated for the presence of thyroid, pituitary, or adrenal endocrinopathies. The patient should be referred to an endocrinologist if an endocrinopathy is suspected. Thyroid-stimulating hormone (TSH) and free triiodothyronine and thyroxine levels should be measured to determine whether thyroid abnormalities are present. Pituitary hormone levels and function tests (e.g., TSH, growth hormone, luteinizing hormone, follicle-stimulating hormone, testosterone, prolactin, adrenocorticotropic hormone [ACTH] levels, and ACTH stimulation test) and magnetic resonance imaging (MRI) of the brain (with detailed pituitary sections) may help to differentiate primary pituitary insufficiency from primary adrenal insufficiency.

Table 4 Management Guidelines for Endocrine Events

Event	Management
Asymptomatic hypothyroidism	• Continue atezolizumab.<br>• Initiate treatment with thyroid replacement hormone.<br>• Monitor TSH closely.
Symptomatic hypothyroidism	• Withhold atezolizumab.<br>• Initiate treatment with thyroid replacement hormone.<br>• Monitor TSH closely.<br>• Consider patient referral to endocrinologist.<br>• Resume atezolizumab when symptoms are controlled and thyroid function is improving.
Asymptomatic hyperthyroidism	**TSH ≥ 0.1 mU/L and < 0.5 mU/L:**<br>• Continue atezolizumab.<br>• Monitor TSH every 4 weeks.<br>• Consider patient referral to endocrinologist.<br>**TSH < 0.1 mU/L:**<br>• Follow guidelines for symptomatic hyperthyroidism.<br>• Consider patient referral to endocrinologist.
Symptomatic hyperthyroidism	• Withhold atezolizumab.<br>• Initiate treatment with anti-thyroid drug such as methimazole or carbimazole as needed.<br>• Consider patient referral to endocrinologist.<br>• Resume atezolizumab when symptoms are controlled and thyroid function is improving.<br>• Permanently discontinue atezolizumab and contact Medical Monitor for life-threatening immune-mediated hyperthyroidism.[c]

Event	Management
Symptomatic adrenal insufficiency, Grades 2–4	• Withhold atezolizumab for up to 12 weeks after event onset. [a]<br>• Refer patient to endocrinologist.<br>• Perform appropriate imaging.<br>• Initiate treatment with corticosteroids equivalent to 1–2 mg/kg/day IV methylprednisolone and convert to 1–2 mg/kg/day oral prednisone or equivalent upon improvement.<br>• If event resolves to Grade 1 or better and patient is stable on replacement therapy, resume atezolizumab. [b]<br>• If event does not resolve to Grade 1 or better or patient is not stable on replacement therapy while withholding atezolizumab, permanently discontinue atezolizumab and contact Medical Monitor. [c]
Hyperglycemia, Grade 1 or 2	• Continue atezolizumab.<br>• Investigate for diabetes. If patient has Type 1 diabetes, treat as a Grade 3 event. If patient does not have Type 1 diabetes, treat as per institutional guidelines.<br>• Monitor for glucose control.
Hyperglycemia, Grade 3 or 4	• Withhold atezolizumab.<br>• Initiate treatment with insulin.<br>• Evaluate for diabetic ketoacidosis and manage as per institutional guidelines.<br>• Monitor for glucose control.<br>• Resume atezolizumab when symptoms resolve and glucose levels are stable.

Event	Management
Hypophysitis (pan-hypopituitarism), Grade 2 or 3	• Withhold atezolizumab for up to 12 weeks after event onset. [a]<br>• Refer patient to endocrinologist.<br>• Perform brain MRI (pituitary protocol).<br>• Initiate treatment with corticosteroids equivalent to 1–2 mg/kg/day IV methylprednisolone and convert to 1–2 mg/kg/day oral prednisone or equivalent upon improvement.<br>• Initiate hormone replacement if clinically indicated.<br>• If event resolves to Grade 1 or better, resume atezolizumab. [b]<br>• If event does not resolve to Grade 1 or better while withholding atezolizumab, permanently discontinue atezolizumab and contact Medical Monitor. [c]<br>• For recurrent hypophysitis, treat as a Grade 4 event.
Hypophysitis (pan-hypopituitarism), Grade 4	• Permanently discontinue atezolizumab and contact Medical Monitor. [c]<br>• Refer patient to endocrinologist.<br>• Perform brain MRI (pituitary protocol).<br>• Initiate treatment with corticosteroids equivalent to 1–2 mg/kg/day IV methylprednisolone and convert to 1–2 mg/kg/day oral prednisone or equivalent upon improvement.<br>• Initiate hormone replacement if clinically indicated.

[a] Atezolizumab may be withheld for a longer period of time (i.e., > 12 weeks after event onset) to allow for corticosteroids (if initiated) to be reduced to the equivalent of ≤ 10 mg/day oral prednisone. The acceptable length of the extended period of time must be based on assessment of benefit-risk by the investigator and in alignment with the protocol requirement for duration of treatment and documented by the investigator. Medical Monitor is available to advise as needed.

[b] If corticosteroids have been initiated, they must be tapered over ≥ 1 month to the equivalent of ≤ 10 mg/day oral prednisone before atezolizumab can be resumed.

[c] Resumption of atezolizumab may be considered in patients who are deriving benefit and have fully recovered from the immune-mediated event. The decision to rechallenge patients with atezolizumab should be based on investigator's assessment of benefit-risk and documented by the investigator (or an appropriate delegate). Medical Monitor is available to advise as needed.

OCULAR EVENTS

An ophthalmologist should evaluate visual complaints (e.g., uveitis, retinal events). Management guidelines for ocular events are provided inTable 5.

Table 5 Management Guidelines for Ocular Events

Event	Management
Ocular event, Grade 1	• Continue atezolizumab.<br>• Patient referral to ophthalmologist is strongly recommended.<br>• Initiate treatment with topical corticosteroid eye drops and topical immunosuppressive therapy.<br>• If symptoms persist, treat as a Grade 2 event.
Ocular event, Grade 2	• Withhold atezolizumab for up to 12 weeks after event onset. [a]<br>• Patient referral to ophthalmologist is strongly recommended.<br>• Initiate treatment with topical corticosteroid eye drops and topical immunosuppressive therapy.<br>• If event resolves to Grade 1 or better, resume atezolizumab. [b]<br>• If event does not resolve to Grade 1 or better while withholding atezolizumab, permanently discontinue atezolizumab and contact Medical Monitor. [c]
Ocular event, Grade 3 or 4	• Permanently discontinue atezolizumab and contact Medical Monitor. [c]<br>• Refer patient to ophthalmologist.<br>• Initiate treatment with corticosteroids equivalent to 1–2 mg/kg/day oral prednisone.<br>• If event resolves to Grade 1 or better, taper corticosteroids over ≥1 month.

[a] Atezolizumab may be withheld for a longer period of time (i.e., >12 weeks after event onset) to allow for corticosteroids (if initiated) to be reduced to the equivalent of ≤10 mg/day oral prednisone. The acceptable length of the extended period of time must be based on assessment of benefit-risk by the investigator and in alignment with the protocol requirement for duration of treatment and documented by the investigator. Medical Monitor is available to advise as needed.

[b] If corticosteroids have been initiated, they must be tapered over ≥1 month to the equivalent of ≤10 mg/day oral prednisone before atezolizumab can be resumed.

[c] Resumption of atezolizumab may be considered in patients who are deriving benefit and have fully recovered from the immune-mediated event. The decision to rechallenge patients with atezolizumab should be based on investigator's assessment of benefit-risk and documented by the investigator (or an appropriate delegate). Medical Monitor is available to advise as needed.

IMMUNE-MEDIATED MYOCARDITIS

Immune-mediated myocarditis has been associated with the administration of atezolizumab. Immune-mediated myocarditis should be suspected in any patient presenting with signs or symptoms suggestive of myocarditis, including, but not limited to, laboratory (e.g., BNP [B-Natriuretic Peptide]) or cardiac imaging abnormalities, dyspnea, chest pain, palpitations, fatigue, decreased exercise tolerance, or syncope. Myocarditis may also be a clinical manifestation of myositis and should be managed accordingly Immune-mediated myocarditis needs to be

distinguished from myocarditis resulting from infection (commonly viral, e.g., in a patient who reports a recent history of gastrointestinal illness), ischemic events, underlying arrhythmias, exacerbation of preexisting cardiac conditions, or progression of malignancy.

All patients with possible myocarditis should be urgently evaluated by performing cardiac enzyme assessment, an ECG, a chest X-ray, an echocardiogram, and a cardiac MRI as appropriate per institutional guidelines. A cardiologist should be consulted. An endomyocardial biopsy may be considered to enable a definitive diagnosis and appropriate treatment, if clinically indicated.

Patients with signs and symptoms of myocarditis, in the absence of an identified alternate etiology, should be treated according to the guidelines inTable 6.

Table 6 Management Guidelines for Immune-Related Myocarditis

Event	Management
Immune-mediated myocarditis, Grades 2–4	• Permanently discontinue atezolizumab and contact Medical Monitor. [a]<br>• Refer patient to cardiologist.<br>• Initiate treatment as per institutional guidelines and consider antiarrhythmic drugs, temporary pacemaker, ECMO, or VAD as appropriate.<br>• Initiate treatment with corticosteroids equivalent to 1–2 mg/kg/day IV methylprednisolone and convert to 1–2 mg/kg/day oral prednisone or equivalent upon improvement.<br>• If event does not improve within 48 hours after initiating corticosteroids, consider adding an immunosuppressive agent.<br>• If event resolves to Grade 1 or better, taper corticosteroids over ≥ 1 month.

ECMO = extracorporeal membrane oxygenation; VAD = ventricular assist device.

[a] Resumption of atezolizumab may be considered in patients who are deriving benefit and have fully recovered from the immune-mediated event. The decision to rechallenge patients with atezolizumab should be based on investigator's assessment of benefit-risk and documented by the investigator (or an appropriate delegate). Medical Monitor is available to advise as needed.

INFUSION-RELATED REACTIONS AND CYTOKINE-RELEASE SYNDROME

No premedication is indicated for the administration of Cycle 1 of atezolizumab. However, patients who experience an infusion-related reaction (IRR) or cytokine-release syndrome (CRS) with atezolizumab may receive premedication with antihistamines, anti-pyretics, and/or analgesics (e.g., acetaminophen) for subsequent infusions. Metamizole (dipyrone) is prohibited in treating atezolizumab-associated IRRs because of its potential for causing agranulocytosis.

IRRs are known to occur with the administration of monoclonal antibodies and have been reported with atezolizumab. These reactions, which are thought to be due to release of cytokines and/or other chemical mediators, occur within 24 hours of atezolizumab administration and are generally mild to moderate in severity.

CRS is defined as a supraphysiologic response following administration of any immune therapy that results in activation or engagement of endogenous or infused T cells and/or other immune effector cells. Symptoms can be progressive, always include fever at the onset, and may include hypotension, capillary leak (hypoxia), and end-organ dysfunction (Lee et al. 2019). CRS has been well documented with chimeric antigen receptor T-cell therapies and bispecific T-cell engager antibody therapies but has also been reported with immunotherapies that target PD-1 or PD-L1 (Rotz et al. 2017; Adashek and Feldman 2019), including atezolizumab.

There may be significant overlap in signs and symptoms of IRRs and CRS, and in recognition of the challenges in clinically distinguishing between the two, consolidated guidelines for medical management of IRRs and CRS are provided in table 7.

Severe COVID-19 appears to be associated with a cytokine-release syndrome (CRS) involving the inflammatory cytokines interleukin (IL)-6, IL-10, IL-2, and interferon-γ (Merad and Martin 2020). If a patient develops suspected CRS during the study, a differential diagnosis should include COVID-19, which should be confirmed or refuted through assessment of exposure history, appropriate laboratory testing, and clinical or radiologic evaluations per investigator judgment. If a diagnosis of COVID-19 is confirmed, the disease should be managed as per local or institutional guidelines.

Table 7 Management Guidelines for Infusion-Related Reactions and Cytokine-Release Syndrome

Event	Management
Grade 1 [a]<br>fever [b] with or without constitutional symptoms	• Immediately interrupt infusion.<br>• Upon symptom resolution, wait for 30 minutes and then restart infusion at half the rate being given at the time of event onset.<br>• If the infusion is tolerated at the reduced rate for 30 minutes, the infusion rate may be increased to the original rate.<br>• If symptoms recur, discontinue infusion of this dose.<br>• Administer symptomatic treatment, [c] including maintenance of IV fluids for hydration.<br>• In case of rapid decline or prolonged CRS (>2 days) or in patients with significant symptoms and/or comorbidities, consider managing as per Grade 2.<br>• For subsequent infusions, consider administration of oral premedication with antihistamines, anti-pyretics, and/or analgesics, and monitor closely for IRRs and/or CRS.
Grade 2 [a]<br>fever [b] with hypotension not requiring vasopressors<br>**and/or**<br>hypoxia requiring low-flow oxygen [d] by nasal cannula or blow-by	• Immediately interrupt infusion.<br>• Upon symptom resolution, wait for 30 minutes and then restart infusion at half the rate being given at the time of event onset.<br>• If symptoms recur, discontinue infusion of this dose.<br>• Administer symptomatic treatment. [c]<br>• For hypotension, administer IV fluid bolus as needed.<br>• Monitor cardiopulmonary and other organ function closely (in the ICU, if appropriate). Administer IV fluids as clinically indicated and manage constitutional symptoms and organ toxicities as per institutional practice.<br>• Rule out other inflammatory conditions that can mimic CRS (e.g., sepsis). If no improvement within 24 hours, initiate workup and assess for signs and symptoms of HLH or MAS.<br>• Consider IV corticosteroids (e.g., methylprednisolone 2 mg/kg/day or dexamethasone 10 mg every 6 hours).<br>• Consider anti-cytokine therapy.<br>• Consider hospitalization until complete resolution of symptoms. If no improvement within 24 hours, manage as per Grade 3, that is, hospitalize patient (monitoring in the ICU is recommended), permanently discontinue atezolizumab, and contact Medical Monitor.<br>• If symptoms resolve to Grade 1 or better for 3 consecutive days, next dose of atezolizumab may be administered. For subsequent infusions, consider administration of oral premedication with antihistamines, anti-pyretics, and/or analgesics and monitor closely for IRRs and/or CRS.<br>• If symptoms do not resolve to Grade 1 or better for 3 consecutive days, contact Medical Monitor.

Event	Management
Grade 3 [a]<br>fever [b] with hypotension requiring a vasopressor (with or without vasopressin)<br>**and/or**<br>hypoxia requiring high-flow oxygen [d] by nasal cannula, face mask, non-rebreather mask, or venturi mask	• Permanently discontinue atezolizumab and contact Medical Monitor. [e]<br>• Administer symptomatic treatment. [c]<br>• For hypotension, administer IV fluid bolus and vasopressor as needed.<br>• Monitor cardiopulmonary and other organ function closely; monitoring in the ICU is recommended. Administer IV fluids as clinically indicated, and manage constitutional symptoms and organ toxicities as per institutional practice.<br>• Rule out other inflammatory conditions that can mimic CRS (e.g., sepsis). If no improvement within 24 hours, initiate workup and assess for signs and symptoms of HLH or MAS.<br>• Administer IV corticosteroids (e.g., methylprednisolone 2 mg/kg/day or dexamethasone 10 mg every 6 hours).<br>• Consider anti-cytokine therapy.<br>• Hospitalize patient until complete resolution of symptoms. If no improvement within 24 hours, manage as per Grade 4, that is, admit patient to ICU and initiate hemodynamic monitoring, mechanical ventilation, and/or IV fluids and vasopressors as needed; for patients who are refractory to anti-cytokine therapy, experimental treatments may be considered at the discretion of the investigator and in consultation with the Medical Monitor.
Grade 4 [a]<br>fever [b] with hypotension requiring multiple vasopressors (excluding vasopressin)<br>**and/or**<br>hypoxia requiring oxygen by positive pressure (e.g., CPAP, BiPAP, intubation and mechanical ventilation)	• Permanently discontinue atezolizumab and contact Medical Monitor. [e]<br>• Administer symptomatic treatment. [c]<br>• Admit patient to ICU and initiate hemodynamic monitoring, mechanical ventilation, and/or IV fluids and vasopressors as needed. Monitor other organ function closely. Manage constitutional symptoms and organ toxicities as per institutional practice.<br>• Rule out other inflammatory conditions that can mimic CRS (e.g., sepsis). If no improvement within 24 hours, initiate workup and assess for signs and symptoms of HLH or MAS.<br>• Administer IV corticosteroids (e.g., methylprednisolone 2 mg/kg/day or dexamethasone 10 mg every 6 hours).<br>• Consider anti-cytokine therapy. For patients who are refractory to anti-cytokine therapy, experimental treatments [f] may be considered at the discretion of the investigator and in consultation with the Medical Monitor.<br>• Hospitalize patient until complete resolution of symptoms.

ASTCT = American Society for Transplantation and Cellular Therapy; BiPAP = bi-level positive airway pressure; CAR = chimeric antigen receptor; CPAP = continuous positive airway pressure; CRS = cytokine-release syndrome; CTCAE = Common Terminology Criteria for Adverse Events; eCRF = electronic Case Report Form; HLH = hemophagocytic lymphohistiocytosis; ICU = intensive care unit; IRR = infusion-related reaction; IV=intraveous; MAS = macrophage activation syndrome; NCCN = National Cancer Comprehensive Network; NCI = National Cancer Institute.

Note: These management guidelines have been adapted from the NCCN guidelines for the management of CAR T-cell–related toxicities (Version 2.2019).

[a] Grading system for these management guidelines is based on ASTCT consensus grading for CRS. NCI CTCAE (version as specified in the protocol) should be used when reporting severity of IRRs, CRS, or organ toxicities associated with CRS on the Adverse Event eCRF. Organ toxicities associated with CRS should not influence overall CRS grading.

[b] Fever is defined as temperature ≥ 38°C not attributable to any other cause. In patients who develop CRS and who then receive anti-pyretic, anti-cytokine, or corticosteroid therapy, fever is no longer required when subsequently determining event severity (grade). In this case, the grade is driven by the presence of hypotension and/or hypoxia.

[c] Symptomatic treatment may include oral or IV antihistamines, anti-pyretics, analgesics, bronchodilators, and/or oxygen. For bronchospasm, urticaria, or dyspnea, additional treatment may be administered as per institutional practice.

[d] Low flow is defined as oxygen delivered at ≤ 6 L/min, and high flow is defined as oxygen delivered at > 6 L/min.

[e] Resumption of atezolizumab may be considered in patients who are deriving benefit and have fully recovered from the immune-mediated event. The decision to rechallenge patients with atezolizumab should be based on investigator's assessment of benefit-risk and documented by the investigator (or an appropriate delegate). Medical Monitor is available to advise as needed. For subsequent infusions, administer oral premedication with antihistamines, anti-pyretics, and/or analgesics, and monitor closely for IRRs and/or CRS. Premedication with corticosteroids and extending the infusion time may also be considered after assessing the benefit–risk ratio.

[f] Refer to Riegler et al. (2019)

PANCREATIC EVENTS

Symptoms of abdominal pain associated with elevations of amylase and lipase, suggestive of pancreatitis, have been associated with the administration of atezolizumab.

The differential diagnosis of acute abdominal pain should include pancreatitis. Appropriate workup should include an evaluation for ductal obstruction, as well as serum amylase and lipase tests. Management guidelines for pancreatic events, including pancreatitis, are provided in Table 8.

Table 8 Management Guidelines for Pancreatic Events, Including Pancreatitis

Event	Management
Amylase and/or lipase elevation, Grade 2	**Amylase and/or lipase > 1.5–2.0 × ULN:**<br>• Continue atezolizumab.<br>• Monitor amylase and lipase weekly.<br>• For prolonged elevation (e.g., > 3 weeks), consider treatment with corticosteroids equivalent to 10 mg/day oral prednisone.<br>**Asymptomatic with amylase and/or lipase > 2.0–5.0 × ULN:**<br>• Treat as Grade 3.
Amylase and/or lipase elevation, Grade 3 or 4	• Withhold atezolizumab for up to 12 weeks after event onset. [a]<br>• Refer patient to GI specialist.<br>• Monitor amylase and lipase every other day.<br>• If no improvement, consider treatment with corticosteroids equivalent to 1–2 mg/kg/day oral prednisone.<br>• If event resolves to Grade 1 or better, resume atezolizumab. [b]<br>• If event does not resolve to Grade 1 or better while withholding atezolizumab, permanently discontinue atezolizumab and contact Medical Monitor. [c]<br>• For recurrent events, permanently discontinue atezolizumab and contact Medical Monitor. [c]

Event	Management
Immune-mediated pancreatitis, Grade 2 or 3	• Withhold atezolizumab for up to 12 weeks after event onset.[a]<br>• Refer patient to GI specialist.<br>• Initiate treatment with corticosteroids equivalent to 1–2 mg/kg/day IV methylprednisolone and convert to 1–2 mg/kg/day oral prednisone or equivalent upon improvement.<br>• If event resolves to Grade 1 or better, resume atezolizumab.[b]<br>• If event does not resolve to Grade 1 or better while withholding atezolizumab, permanently discontinue atezolizumab and contact Medical Monitor.[c]<br>• For recurrent events, permanently discontinue atezolizumab and contact Medical Monitor.[c]
Immune-mediated pancreatitis, Grade 4	• Permanently discontinue atezolizumab and contact Medical Monitor.[c]<br>• Refer patient to GI specialist.<br>• Initiate treatment with corticosteroids equivalent to 1–2 mg/kg/day IV methylprednisolone and convert to 1–2 mg/kg/day oral prednisone or equivalent upon improvement.<br>• If event does not improve within 48 hours after initiating corticosteroids, consider adding an immunosuppressive agent.<br>• If event resolves to Grade 1 or better, taper corticosteroids over ≥1 month.

GI=gastrointestinal.

[a] Atezolizumab may be withheld for a longer period of time (i.e., >12 weeks after event onset) to allow for corticosteroids (if initiated) to be reduced to the equivalent of ≤10 mg/day oral prednisone. The acceptable length of the extended period of time must be based on assessment of benefit-risk by the investigator and in alignment with the protocol requirement for duration of treatment and documented by the investigator. Medical Monitor is available to advise as needed.

[b] If corticosteroids have been initiated, they must be tapered over ≥1 month to the equivalent of ≤10 mg/day oral prednisone before atezolizumab can be resumed.

[c] Resumption of atezolizumab may be considered in patients who are deriving benefit and have fully recovered from the immune-mediated event. The decision to rechallenge patients with atezolizumab should be based on investigator's assessment of benefit-risk and documented by the investigator (or an appropriate delegate). Medical Monitor is available to advise as needed.

DERMATOLOGIC EVENTS

Treatment-emergent rash has been associated with atezolizumab. The majority of cases of rash were mild in severity and self limited, with or without pruritus. Although uncommon, cases of severe cutaneous adverse reactions such as Stevens-Johnson syndrome and toxic epidermal necrolysis have been reported with atezolizumab. A dermatologist should evaluate persistent and/or severe rash or pruritus. A biopsy should be considered unless contraindicated. Management guidelines for dermatologic events are provided in Table 9.

Table 9 Management Guidelines for Dermatologic Events

Event	Management
Dermatologic event, Grade 1	• Continue atezolizumab.<br>• Consider treatment with topical corticosteroids and/or other symptomatic therapy (e.g., antihistamines).
Dermatologic event, Grade 2	• Continue atezolizumab.<br>• Consider patient referral to dermatologist for evaluation and, if indicated, biopsy.<br>• Initiate treatment with topical corticosteroids.<br>• Consider treatment with higher-potency topical corticosteroids if event does not improve.<br>• If unresponsive to topical corticosteroids, consider oral prednisone 0.5 mg/kg/day.
Dermatologic event, Grade 3	• Withhold atezolizumab for up to 12 weeks after event onset. [a]<br>• Refer patient to dermatologist for evaluation and, if indicated, biopsy.<br>• Initiate treatment with corticosteroids equivalent to 10 mg/day oral prednisone, increasing dose to 1–2 mg/kg/day if event does not improve within 48–72 hours.<br>• If event resolves to Grade 1 or better, resume atezolizumab. [b]<br>• If event does not resolve to Grade 1 or better while withholding atezolizumab, permanently discontinue atezolizumab and contact Medical Monitor. [c]
Dermatologic event, Grade 4	• Permanently discontinue atezolizumab and contact Medical Monitor. [c]
Stevens-Johnson syndrome or toxic epidermal necrolysis, (any grade)	Additional guidance for Stevens-Johnson syndrome or toxic epidermal necrolysis:<br>• Withhold atezolizumab for suspected Stevens-Johnson syndrome or toxic epidermal necrolysis.<br>• Confirm diagnosis by referring patient to a specialist (dermatologist, ophthalmologist or urologist as relevant) for evaluation and, if indicated, biopsy.<br>• Follow the applicable treatment and management guidelines above.<br>• If Stevens-Johnson syndrome or toxic epidermal necrolysis is confirmed, permanently discontinue atezolizumab.

[a] Atezolizumab may be withheld for a longer period of time (i.e., > 12 weeks after event onset) to allow for corticosteroids (if initiated) to be reduced to the equivalent of ≤ 10 mg/day oral prednisone. The acceptable length of the extended period of time must be based on assessment of benefit-risk by the investigator and in alignment with the protocol requirement for duration of treatment and documented by the investigator. Medical Monitor is available to advise as needed.

[b] If corticosteroids have been initiated, they must be tapered over ≥ 1 month to the equivalent of ≤ 10 mg/day oral prednisone before atezolizumab can be resumed.

[c] Resumption of atezolizumab may be considered in patients who are deriving benefit and have fully recovered from the immune-mediated event. The decision to rechallenge patients with atezolizumab should be based on investigator's assessment of benefit-risk and documented by the investigator (or an appropriate delegate). Medical Monitor is available to advise as needed.

NEUROLOGIC DISORDERS

Myasthenia gravis and Guillain-Barré syndrome have been observed with single-agent atezolizumab. Patients may present with signs and symptoms of sensory and/or motor neuropathy. Diagnostic work-up is essential for an accurate characterization to differentiate between alternative etiologies. Management guidelines for neurologic disorders are provided in Table 10.

Table 10 Management Guidelines for Neurologic Disorders

Event	Management
Immune-mediated neuropathy, Grade 1	• Continue atezolizumab.<br>• Investigate etiology.
Immune-mediated neuropathy, Grade 2	• Withhold atezolizumab for up to 12 weeks after event onset. [a]<br>• Investigate etiology and refer patient to neurologist.<br>• Initiate treatment as per institutional guidelines.<br>• If event resolves to Grade 1 or better, resume atezolizumab. [b]<br>• If event does not resolve to Grade 1 or better while withholding atezolizumab, permanently discontinue atezolizumab and contact Medical Monitor. [c]
Immune-mediated neuropathy, Grade 3 or 4	• Permanently discontinue atezolizumab and contact Medical Monitor. [c]<br>• Refer patient to neurologist.<br>• Initiate treatment as per institutional guidelines.
Myasthenia gravis and Guillain-Barré syndrome (any grade)	• Permanently discontinue atezolizumab and contact Medical Monitor. [c]<br>• Refer patient to neurologist.<br>• Initiate treatment as per institutional guidelines.<br>• Consider initiation of corticosteroids equivalent to 1–2 mg/kg/day oral or IV prednisone.

[a] Atezolizumab may be withheld for a longer period of time (i.e., > 12 weeks after event onset) to allow for corticosteroids (if initiated) to be reduced to the equivalent of ≤ 10 mg/day oral prednisone. The acceptable length of the extended period of time must be based on assessment of benefit-risk by the investigator and in alignment with the protocol requirement for duration of treatment and documented by the investigator. Medical Monitor is available to advise as needed.

[b] If corticosteroids have been initiated, they must be tapered over ≥ 1 month to the equivalent of ≤ 10 mg/day oral prednisone before atezolizumab can be resumed.

[c] Resumption of atezolizumab may be considered in patients who are deriving benefit and have fully recovered from the immune-mediated event. The decision to rechallenge patients with atezolizumab should be based on investigator's assessment of benefit-risk and documented by the investigator (or an appropriate delegate). Medical Monitor is available to advise as needed.

IMMUNE-MEDIATED MENINGOENCEPHALITIS

Immune-mediated meningoencephalitis is an identified risk associated with the administration of atezolizumab. Immune-mediated meningoencephalitis should be suspected in any patient

presenting with signs or symptoms suggestive of meningitis or encephalitis, including, but not limited to, headache, neck pain, confusion, seizure, motor or sensory dysfunction, and altered or depressed level of consciousness.
Encephalopathy from metabolic or electrolyte imbalances needs to be distinguished from potential meningoencephalitis resulting from infection (bacterial, viral, or fungal) or progression of malignancy, or secondary to a paraneoplastic process.
All patients being considered for meningoencephalitis should be urgently evaluated with a CT scan and/or MRI scan of the brain to evaluate for metastasis, inflammation, or edema. If deemed safe by the treating physician, a lumbar puncture should be performed and a neurologist should be consulted.
Patients with signs and symptoms of meningoencephalitis, in the absence of an identified alternate etiology, should be treated according to the guidelines in Table 11.

Table 11 Management Guidelines for Immune-Mediated Meningoencephalitis

Event	Management
Immune-mediated meningoencephalitis, all grades	• Permanently discontinue atezolizumab and contact Medical Monitor. [a]<br>• Refer patient to neurologist.<br>• Initiate treatment with corticosteroids equivalent to 1–2 mg/kg/day IV methylprednisolone and convert to 1–2 mg/kg/day oral prednisone or equivalent upon improvement.<br>• If event does not improve within 48 hours after initiating corticosteroids, consider adding an immunosuppressive agent.<br>• If event resolves to Grade 1 or better, taper corticosteroids over ≥1 month.

[a] Resumption of atezolizumab may be considered in patients who are deriving benefit and have fully recovered from the immune-mediated event. The decision to rechallenge patients with atezolizumab should be based on investigator's assessment of benefit-risk and documented by the investigator (or an appropriate delegate). Medical Monitor is available to advise as needed.

RENAL EVENTS

Immune-mediated nephritis has been associated with the administration of atezolizumab. Eligible patients must have adequate renal function. Renal function, including serum creatinine, should be monitored throughout study treatment. Patients with abnormal renal function should be evaluated and treated for other more common etiologies (including prerenal and postrenal causes, and concomitant medications such as non-steroidal anti-inflammatory drugs). Refer the patient to a renal specialist if clinically indicated. A renal biopsy may be required to enable a definitive diagnosis and appropriate treatment.
Patients with signs and symptoms of nephritis, in the absence of an identified alternate etiology, should be treated according to the guidelines in Table 12.

Table 12 Management Guidelines for Renal Events

Event	Management
Renal event, Grade 1	• Continue atezolizumab.<br>• Monitor kidney function closely, including creatinine and urine protein, until values resolve to within normal limits or to baseline values.
Renal event, Grade 2	• Withhold atezolizumab for up to 12 weeks after event onset. [a]<br>• Refer patient to renal specialist.<br>• Initiate treatment with corticosteroids equivalent to 1–2 mg/kg/day oral prednisone.<br>• If event resolves to Grade 1 or better, resume atezolizumab. [b]<br>• If event does not resolve to Grade 1 or better while withholding atezolizumab, permanently discontinue atezolizumab and contact Medical Monitor. [c]
Renal event, Grade 3 or 4	• Permanently discontinue atezolizumab and contact Medical Monitor.<br>• Refer patient to renal specialist and consider renal biopsy.<br>• Initiate treatment with corticosteroids equivalent to 1–2 mg/kg/day oral prednisone.<br>• If event does not improve within 48 hours after initiating corticosteroids, consider adding an immunosuppressive agent.<br>• If event resolves to Grade 1 or better, taper corticosteroids over ≥ 1 month.

[a] Atezolizumab may be withheld for a longer period of time (i.e., > 12 weeks after event onset) to allow for corticosteroids (if initiated) to be reduced to the equivalent of ≤ 10 mg/day oral prednisone. The acceptable length of the extended period of time must be based on assessment of benefit-risk by the investigator and in alignment with the protocol requirement for duration of treatment and documented by the investigator. Medical Monitor is available to advise as needed.

[b] If corticosteroids have been initiated, they must be tapered over ≥ 1 month to the equivalent of ≤ 10 mg/day oral prednisone before atezolizumab can be resumed.

[c] Resumption of atezolizumab may be considered in patients who are deriving benefit and have fully recovered from the immune-mediated event. The decision to rechallenge patients with atezolizumab should be based on investigator's assessment of benefit-risk and documented by the investigator (or an appropriate delegate). Medical Monitor is available to advise as needed.

IMMUNE-MEDIATED MYOSITIS

Myositis or inflammatory myopathies are a group of disorders sharing the common feature of inflammatory muscle injury; dermatomyositis and polymyositis are among the most common disorders. Initial diagnosis is based on clinical (muscle weakness, muscle pain, skin rash in dermatomyositis), biochemical (serum creatine kinase increase), and imaging (electromyography/MRI) features, and is confirmed with a muscle biopsy. Patients with possible myositis should be referred to a rheumatologist or neurologist. Patients with possible myositis should be monitored for signs of miocarditis.

Patients with signs and symptoms of myositis, in the absence of an identified alternate etiology, should be treated according to the guidelines in Table 13.

Table 13 Management Guidelines for Immune-Mediated Myositis

Event	Management
Immune-mediated myositis, Grade 1	• Continue atezolizumab.<br>• Refer patient to rheumatologist or neurologist.<br>• Initiate treatment as per institutional guidelines.
Immune-mediated myositis, Grade 2	• Withhold atezolizumab for up to 12 weeks after event onset [a] and contact Medical Monitor.<br>• Refer patient to rheumatologist or neurologist.<br>• Initiate treatment as per institutional guidelines.<br>• Consider treatment with corticosteroids equivalent to 1–2 mg/kg/day IV methylprednisolone and convert to 1–2 mg/kg/day oral prednisone or equivalent upon improvement.<br>• If corticosteroids are initiated and event does not improve within 48 hours after initiating corticosteroids, consider adding an immunosuppressive agent.<br>• If event resolves to Grade 1 or better, resume atezolizumab. [b]<br>• If event does not resolve to Grade 1 or better while withholding atezolizumab, permanently discontinue atezolizumab and contact Medical Monitor. [c]
Immune-mediated myositis, Grade 3	• Withhold atezolizumab for up to 12 weeks after event onset [a] and contact Medical Monitor.<br>• Refer patient to rheumatologist or neurologist.<br>• Initiate treatment as per institutional guidelines.<br>• Respiratory support may be required in more severe cases.<br>• Initiate treatment with corticosteroids equivalent to 1–2 mg/kg/day IV methylprednisolone, or higher-dose bolus if patient is severely compromised (e.g., cardiac or respiratory symptoms, dysphagia, or weakness that severely limits mobility); convert to 1–2 mg/kg/day oral prednisone or equivalent upon improvement.<br>• If event does not improve within 48 hours after initiating corticosteroids, consider adding an immunosuppressive agent.<br>• If event resolves to Grade 1 or better, resume atezolizumab. [b]<br>• If event does not resolve to Grade 1 or better while withholding atezolizumab, permanently discontinue atezolizumab and contact Medical Monitor.<br>• For recurrent events, treat as Grade 4 event. Permanently discontinue atezolizumab and contact Medical Monitor. [c]

Event	Management
Immune-mediated myositis, Grade 4	• Permanently discontinue atezolizumab and contact Medical Monitor. [c]<br>• Refer patient to rheumatologist or neurologist.<br>• Initiate treatment as per institutional guidelines.<br>• Respiratory support may be required in more severe cases.<br>• Initiate treatment with corticosteroids equivalent to 1–2 mg/kg/day IV methylprednisolone, or higher-dose bolus if patient is severely compromised (e.g., cardiac or respiratory symptoms, dysphagia, or weakness that severely limits mobility); convert to 1–2 mg/kg/day oral prednisone or equivalent upon improvement.<br>• If event does not improve within 48 hours after initiating corticosteroids, consider adding an immunosuppressive agent.<br>• If event resolves to Grade 1 or better, taper corticosteroids over ≥ 1 month.

[a] Atezolizumab may be withheld for a longer period of time (i.e., > 12 weeks after event onset) to allow for corticosteroids (if initiated) to be reduced to the equivalent of ≤ 10 mg/day oral prednisone. The acceptable length of the extended period of time must be based on assessment of benefit-risk by the investigator and in alignment with the protocol requirement for duration of treatment and documented by the investigator. Medical Monitor is available to advise as needed.

[b] If corticosteroids have been initiated, they must be tapered over ≥ 1 month to the equivalent of ≤ 10 mg/day oral prednisone before atezolizumab can be resumed.

[c] Resumption of atezolizumab may be considered in patients who are deriving benefit and have fully recovered from the immune-mediated event. The decision to rechallenge patients with atezolizumab should be based on investigator's assessment of benefit-risk and documented by the investigator (or an appropriate delegate). Medical Monitor is available to advise as needed.

HEMOPHAGOCYTIC LYMPHOHISTIOCYTOSIS AND MACROPHAGE ACTIVATION SYNDROME

Immune-mediated reactions may involve any organ system and may lead to hemophagocytic lymphohistiocytosis (HLH) and macrophage activation syndrome (MAS).

Clinical and laboratory features of severe CRS overlap with HLH, and HLH should be considered when CRS presentation is atypical or prolonged.

Patients with suspected HLH should be diagnosed according to published criteria by McClain and Eckstein (2014). A patient should be classified as having HLH if five of the following eight criteria are met:

Fever ≥ 38.5°C

Splenomegaly

Peripheral blood cytopenia consisting of at least two of the following:

- Hemoglobin < 90 g/L (9 g/dL) (< 100 g/L [10 g/dL] for infants < 4 weeks old)
- Platelet count < 100 × 10^9/L (100,000/μL)

– ANC < 1.0 × 10^9/L (1000/µL)

Fasting triglycerides > 2.992 mmol/L (265 mg/dL) and/or fibrinogen < 1.5 g/L (150 mg/dL)

Hemophagocytosis in bone marrow, spleen, lymph node, or liver

Low or absent natural killer cell activity

Ferritin > 500 mg/L (500 ng/mL)

Soluble IL-2 receptor (soluble CD25) elevated ≥ 2 standard deviations above age-adjusted laboratory-specific norms

Patients with suspected MAS should be diagnosed according to published criteria for systemic juvenile idiopathic arthritis by Ravelli et al. (2016). A febrile patient should be classified as having MAS if the following criteria are met:

Ferritin > 684 mg/L (684 ng/mL)

At least two of the following:

– Platelet count ≤ 181 × 10^9/L (181,000/µL)

– AST ≥ 48 U/L

– Triglycerides > 1.761 mmol/L (156 mg/dL)

– Fibrinogen ≤ 3.6 g/L (360 mg/dL)

Patients with suspected HLH or MAS should be treated according to the guidelines in Table 14.

Table 14 Management Guidelines for Suspected Hemophagocytic Lymphohistiocytosis or Macrophage Activation Syndrome

Event	Management
Suspected HLH or MAS	• Permanently discontinue atezolizumab and contact Medical Monitor.<br>• Consider patient referral to hematologist.<br>• Initiate supportive care, including intensive care monitoring if indicated per institutional guidelines.<br>• Consider initiation of IV corticosteroids, an immunosuppressive agent, and/or anti-cytokine therapy.<br>• If event does not respond to treatment within 24 hours, contact Medical Monitor and initiate treatment as appropriate according to published guidelines (La Rosée 2015; Schram and Berliner 2015; La Rosée et al. 2019).<br>• If event resolves to Grade 1 or better, taper corticosteroids over ≥1 month.

HLH=hemophagocytic lymphohistiocytosis; IV=intravenous; MAS=macrophage activation syndrome.

APPENDIX 8: Patient pre-screening form TELMA

GECP lung cancer research

Av. Meridiana, 358 / 6ªplanta
08027 Barcelona
Tel. 934 302 006
Fax. 934 191 768
Email: gecp@gecp.org
www.gecp.org

TELMA (GECP 18/03-ML40237)

"A phase II open-label study of Atezolizumab in combination with bevacizumab as first line treatment for locally advanced or metastatic high-intermediate tumor mutation burden (TMB) selected non-squamous non-small cell lung cancer (NSCLC) patients"

PRE-SCREENING FORM

Please send to Fundación GECP by Fax (Fax: 93 419 17 68) or

Email (Email: lmorales@gecp.org / asancho@gecp.org / averdu@gecp.org)

PRE-SCREENING NUMBER (Assigned by Fundación GECP)
PRE-SCREENING NUMBER: ST / ____ /____ /____ /____

HOSPITAL DATA	
HOSPITAL: ____________________________<br>INVESTIGATOR: _______________________	FAX OR EMAIL TO RECEIVE RESULT: ___________________

INFORMED CONSENT, DATE OF BIRTH AND CRITERIA		
INFORMED CONSENT DATE: _____ / _____ / _________	DATE OF BIRTH: _____ / _____ / ________	
CRITERIA:<br>Does the patient comply with all inclusion criteria and none of the exclusion?		▫ Yes<br>▫ No
TUMOR SAMPLE:<br>Is there enough tumor sample for the analysis of TMB?		▫ Yes<br>▫ No<br>▫ Unknown
SCREENING DATE: ____ / _____ /______	NAME AND SIGNATURE: _____________________	

LABORATORY RESULTS (To be filled out by Fundación GECP)			
SUFFICIENT SAMPLE:	▫ Yes	▫ No	▫ NA
TMB (≥10 mutations/ MB):	▫ Yes	▫ No	▫ NA
CAN THE PATIENT BE ENROLLED?	▫ Yes	▫ No, specify: __________________	
RESULTS DATE: _____ / _____ / _______	NAME AND SIGNATURE: ______________________		

1

Screening Form_v.1.0_11Apr2019

APPENDIX 9: Foundation one CDx Specimen Instructions

FOUNDATIONONE®CDx

Specimen Instructions

FoundationOne®CDx is an extensively validated tissue-based comprehensive genomic profiling service for all solid tumours. FoundationOne CDx analyses 324 cancer-related genes to provide potentially actionable information to help guide treatment options.[1-3]

Acceptable Samples

- Formalin-fixed paraffin embedded (FFPE) specimens, including cut slide specimens are acceptable.
- Use standard fixation methods to preserve nucleic acid integrity. 10% neutral-buffered formalin for 6-72 hours is industry standard. DO NOT use other fixatives (Bouins, B5, AZF, Holland's).
- Do not decalcify.

☐ **SAMPLE SIZE**

1 When feasible, please send the block + 1 H&E slide.*

10 unstained slides (positively charged and unbaked at 4-5 microns thick) + 1 H&E slide.*

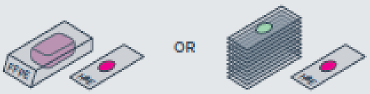

*For smaller samples, providing the original H&E will preserve material for testing.

☐ **SURFACE AREA**

2 **MINIMUM: 25 mm²**

If sending slides, provide 10 unstained slides cut at 4-5 microns thick to achieve a tissue volume of 1 mm³.**

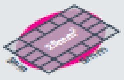

**Specimens with a smaller surface area may meet volume requirements by submitting additional unstained slides (USS) or block.

☐ **TUMOUR CONTENT**

3 **OPTIMUM: 30% TN MINIMUM: 20% TN**

Percent tumour nuclei (%TN) = number of tumour cells divided by total number of all cells with nuclei.

Note for liver specimens: higher tumour content may be required because hepatocyte nuclei have twice the DNA content of other somatic nuclei.

Shipping Instructions

1. Place the samples, FoundationOne CDx requisition form, and any other attachments into the FoundationOne CDx Specimen Shipping Kit.
2. Place the specimen shipping kit (including samples and paperwork) into the shipping pack, first ensuring that primary specimen containers (e.g. blocks, slides) are labelled with two patient-specific identifiers. Seal the shipping pack.
3. Complete the pre-printed shipping labels (if necessary) and apply to shipping pack.
4. Call to request a pick-up or drop the package at your site's designated pick-up location and ship sealed shipping pack to:

[Insert local shipping information]

Intended Use

FoundationOne®CDx is a next generation sequencing based *in vitro* diagnostic device for detection of substitutions, insertion and deletion alterations (indels) and copy number alterations (CNAs) in 324 genes and select gene rearrangements, as well as genomic signatures including tumour mutational burden (TMB) and microsatellite instability (MSI) using DNA isolated from formalin-fixed paraffin embedded (FFPE) tumour tissue specimens. The test is intended as a companion diagnostic to identify patients who may benefit from treatment with therapies in accordance with the approved therapeutic product labeling. Additionally, FoundationOne CDx is intended to provide tumour mutation profiling to be used by qualified health care professionals in accordance with professional guidelines in oncology for patients with solid malignant neoplasms.

For full information on the intended use, assay descriptions, and for detailed performance specifications, refer to the complete FoundationOne CDx label at rochefoundationmedicine.com

References

1. FoundationOne®CDx FDA Approval, 2017. Available at: https://www.accessdata.fda.gov/cdrh_docs/pdf17/P170019a.pdf (Accessed August 2018).
2. FoundationOne®CDx FDA Approval Press Release, 2017. Available at: https://www.fda.gov/NewsEvents/Newsroom/PressAnnouncements/ucm587273.htm (Accessed August 2018).
3. FoundationOne®CDx Technical Specifications 2018. Available at: www.rochefoundationmedicine.com/f1cdxtech.

Foundation Medicine® and FoundationOne® are registered trademarks of Foundation Medicine®, Inc.
Roche is the licensed distributor of Foundation Medicine® products outside of the United States
[local affiliate contact details]

PR/FMI/1808/0015
Date of preparation: September 2018

FOUNDATION MEDICINE® | Roche

APPENDIX 10: Foundation one Liguid technical sheet

Technical Specifications

FOUNDATIONONE®LIQUID

FoundationOne®Liquid is a liquid biopsy test for solid tumors that analyzes circulating tumor DNA (ctDNA) in blood.

Clinical Background

Cell-free DNA (cfDNA) is DNA that circulates freely in the bloodstream. In a cancer patient, tumor cells that undergo apoptosis or necrosis also shed cell-free DNA. The tumor derived cell-free DNA is called circulating tumor DNA (ctDNA). By analyzing cell-free DNA isolated from a patient's blood, we can identify microsatellite instability (MSI)* and clinically relevant genomic alterations in ctDNA and may match them to targeted therapies, immunotherapies and clinical trials.

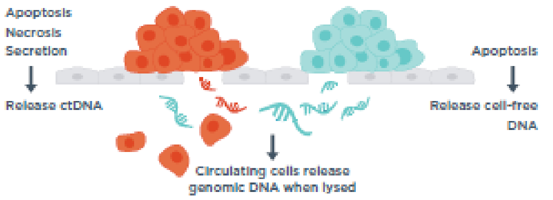

Blood contains circulating cell-free DNA released from healthy tissue and tumor tissue (ctDNA)

Methods

FoundationOne Liquid:

- Analyzes blood samples from patients with solid tumors including lung, breast, colon, etc.
- Uses a hybrid-capture, next-generation sequencing test method combined with proprietary computational algorithms that enable accurate variant calls by discriminating sequencing artifacts from bona fide mutations.
- Identifies four classes of genomic alterations (base substitutions, insertions and deletions, copy number alterations, and rearrangements), and reports high microsatellite instability.
- Evaluates select clinically relevant genomic alterations in 70 commonly altered oncogenes.
- Features an optimized laboratory process to achieve high sensitivity and specificity, with enhanced extraction methodology to generate a large amount of high quality ctDNA.
- Utilizes proprietary technology to accurately identify unique ctDNA fragments from plasma.

PERFORMANCE SPECIFICATIONS

	**Mutant Allele Frequency** (MAF)/ **Tumor Fraction**[1]	**Sensitivity**[2]	**Positive Predictive Value** (PPV)[2]
**Base substitutions**	> 0.5%	99.9% (CI 99.7% – 99.9%)	100% (CI 99.9% – 100%)
	0.25% – 0.5%	95.8% (CI 94.5% – 96.9%)	99.8% (CI 99.3% – 99.9%)
	0.125% – 0.25%	68.4% (CI 65.7% – 70.9%)	96.1% (CI 94.8% – 97.1%)
**Insertions/Deletions** (Indels) (1-40bp)	> 0.5%	99.7% (CI 98.7% – 99.9%)	100% (CI 99.3% – 100%)
	0.25% – 0.5%	87.7% (CI 81.1% – 92.2%)	98.8% (CI 95.4% – 99.8%)
	0.125% – 0.25%	60.5% (CI 52.7% – 67.7%)	96.8% (CI 92.3% – 98.8%)
**Rearrangements**[3]	> 0.5%	100% (CI 85.9% – 100%)	100% (CI 85.9% – 100%)
	0.25% – 0.5%	89.4% (CI 65.5% – 98.2%)	100% (CI 77.1% – 100%)
	0.125% – 0.25%	68.4% (CI 43.5% – 86.4%)	100% (CI 71.7% – 100%)
**Copy Number Amplifications** (CNA)[4]	≥ 20%	95.3% (CI 82.9% – 99.2%)	97.6% (CI 85.9% – 99.9%)
	< 20%	Varies depending on amplitude of CNA and ctDNA fraction	
**Microsatellite Instability** (MSI)[5]	> 2.0%	92.0% (CI 72.5% – 98.6%)	100% (CI 82.2% – 100%)
**Reproducibility** (average concordance between replicates)		97.7% inter-batch precision<br>95.9% intra-batch precision	
**Specimen Type**		Peripheral whole blood (see Specimen Instructions for details)	
**Turnaround Time**[6]		< 2 Weeks	

1. Copy number amplifications were calculated using tumor fraction. **2.** 95% confidence interval. **3.** Performance for gene fusions within targeted introns only. Sensitivity for gene fusions occurring outside targeted introns or in highly repetitive intronic sequence contexts is reduced. **4.** Copy number ≥ 8. **5.** Reported when MSI is determined to be high. **6.** Based on typical turnaround time from receipt of sample.

Reporting

- Test results are provided in an interpretive report, curated by biomedical informatics scientists, and approved by board-certified and licensed pathologists.
- Genomic findings are listed with clinically relevant targeted therapies, immunotherapies, and clinical trials.
- Reported alterations may indicate response or lack of response to therapy (approved or in clinical trials), or may be drivers of oncogenesis based on reported scientific knowledge.
- Reports include microsatellite instability (MSI)* status, a biomarker that may help predict response to checkpoint inhibitors.
- Test results are available via our online portal at *www.foundationmedicine.com*† or by fax.

Additional Features

Mutant Allele Fraction (MAF)

The MAF listed denotes the frequency of the mutant allele identified in the sample. It is reported for base substitutions and insertions and deletions (indels).

Visualization of MAF

The clinical report includes a graphic representation of MAF. If multiple FoundationOne Liquid tests are ordered in the patient's treatment journey, the graphic will show the relative change in MAF which will allow treating physicians to better understand the evolution of a patient's disease and may help to inform the next steps in patient care.

Current Gene List‡

Entire coding sequence (base substitutions, indels, copy number alterations).

APC	*AR*	*ATM*	*BRCA1*	*BRCA2*	*CCND1*	*CD274 (PD-L1)*	*CDH1*	*CDK4*
CDK6	*CDK12*	*CDKN2A*	*CHEK2*	*CRKL*	*EGFR*	*ERBB2*	*ERRFI1*	*FGFR1*
FGFR2	*FOXL2*	*KRAS*	*MDM2*	*MET*	*MYC*	*MYCN*	*NF1*	*PALB2*
PDCD1LG2 (PD-L2)		*PTEN*	*PTPN11*	*RB1*	*SMO*	*STK11*	*TP53*	*VEGFA*

Select Exons§

ABL1	*AKT1*	*ALK*	*ARAF*	*BRAF*	*BTK*	*CTNNB1*	*DDR2*	*ESR1*
EZH2	*FGFR3*	*FLT3*	*GNA11*	*GNAQ*	*GNAS*	*HRAS*	*IDH1*	*IDH2*
JAK2	*JAK3*	*KIT*	*MAP2K1 (MEK1)*	*MAP2K2 (MEK2)*	*MPL*	*MTOR*	*MYD88*	*NPM1*
NRAS	*PDGFRA*	*PDGFRB*	*PIK3CA*	*RAF1*	*RET*	*ROS1*	*TERT*	

Select Rearrangements§

ALK	*EGFR*	*FGFR2*	*FGFR3*	*PDGFRA*	*RET*	*ROS1*

To learn more about our analytical validation based on a prior version of the test called FoundationACT (62 genes), see our publication in the Journal of Molecular Diagnostics:‖ "Analytical validation of a hybrid capture-based next-generation sequencing clinical assay for genomic profiling of cell-free circulating tumor DNA".

References

* Reported when MSI is determined to be high.
† Visit foundationmedicine.com to create an online account.
‡ Current as of August 2018. Please visit foundationmedicine.com for the most up-to-date gene list.
§ Detailed list available upon request.
‖ Clark TA, et al. Analytical validation of a hybrid capture-based next-generation sequencing clinical assay for genomic profiling of cell-free circulating tumor DNA. J of Mol Diagn. 2018;published online ahead of print.

FOUNDATION MEDICINE®

© 2018 Foundation Medicine, Inc. | Foundation Medicine® and FoundationOne® are registered trademarks of Foundation Medicine, Inc.
www.foundationmedicine.com | Tel. 888.988.3639 | Fax 617.418.2290 | MKT-0061-04

APPENDIX 11: Foundation one CDx technical sheet

Please see documents attached to the protocol
