# Supplementary material for: Atezolizumab Plus Bevacizumab as First-line Treatment for Patients With Metastatic Nonsquamous Non–Small Cell Lung Cancer With High Tumor Mutation Burden: A Nonrandomized Controlled Trial
Source: JAMA Oncol. 2022 Dec 15;9(3):344–53. doi: 10.1001/jamaoncol.2022.5959 (PMC9856905; doi:10.1001/jamaoncol.2022.5959)
Supplement: Supplement 2. — eMethods. eResults. eFigure 1. Disposition of Patients in the Study eFigure 2. PD-L1 and TMB Biomarkers eFigure 3. PD-L1 Categories and ORR eFigure 4. TMB and PD-L1 Correlation eFigure 5. TMB and Druggable Alterations in All Screened Patients eFigure 6. High Prevalence Sequence Variations (KRAS or P53) and Outcomes (ORR and PFS-OS) eFigure 7. Sequence Variation Signature and Outcomes (ORR and PFS-OS) eFigure 8. Blood Counts and Biochemistry With Outcomes (ORR and PFS-OS) eFigure 9. Flow Cytometry Analysis of Samples During Treatment and PFS eFigure 10. Flow Cytometry Analysis of Paired Response and Progression Samples eFigure 11. ctDNA Follow-up eFigure 12. Impact of Clinical and Molecular Variables on PFS and OS eTable 1. Associated Comorbidities (Per-Protocol Population) eTable 2. Treatment Compliances [file jamaoncol-e225959-s002.pdf]

Supplemental Online Content

Provencio M, Ortega AL, Coves-Sarto J, et al. Atezolizumab plus bevacizumab as first-line treatment for patients with metastatic nonsquamous non–small cell lung cancer with high tumor mutation burden: a nonrandomized controlled trial. *JAMA Oncol*. Published online December 15, 2022. doi:10.1001/jamaoncol.2022.5959

eMethods.

eResults.

eFigure 1. Disposition of Patients in the Study

eFigure 2. PD-L1 and TMB Biomarkers

eFigure 3. PD-L1 Categories and ORR

eFigure 4. TMB and PD-L1 Correlation

eFigure 5. TMB and Druggable Alterations in All Screened Patients

eFigure 6. High Prevalence Sequence Variations (*KRAS* or *P53*) and Outcomes (ORR and PFS-OS)

eFigure 7. Sequence Variation Signature and Outcomes (ORR and PFS-OS)

eFigure 8. Blood Counts and Biochemistry With Outcomes (ORR and PFS-OS)

eFigure 9. Flow Cytometry Analysis of Samples During Treatment and PFS

eFigure 10. Flow Cytometry Analysis of Paired Response and Progression Samples

eFigure 11. ctDNA Follow-up

eFigure 12. Impact of Clinical and Molecular Variables on PFS and OS

eTable 1. Associated Comorbidities (Per-Protocol Population)

eTable 2. Treatment Compliances

This supplemental material has been provided by the authors to give readers additional information about their work.

© 2022 Provencio M et al. *JAMA Oncology.*

eMethods.

Supplementary Study Design and Patients

Patients who had received prior neoadjuvant or adjuvant chemotherapy, radiotherapy, or chemoradiotherapy with curative intent for non-metastatic disease must have experienced a treatment-free interval of at least 6 months from enrollment since the last chemotherapy, radiotherapy, or chemo-radiotherapy. Active or untreated CNS metastases (previously treated asymptomatic supratentorial and cerebellar metastases were permitted, provided that there was no evidence of interim progression between the completion of CNS-directed therapy and the screening radiographic study, no ongoing requirement for corticosteroids as therapy for CNS disease, and no stereotactic radiation was received within seven days or whole-brain radiation within 14 days prior to enrollment); malignancies other than NSCLC within five years prior to enrollment, with the exception of those with a negligible risk of metastasis or death treated with expected curative outcome; acute or chronic infection with hepatitis B or C virus; HIV positive test; history of autoimmune disease or treatment with systemic immunosuppressive medications within two weeks prior to inclusion; active tuberculosis; severe infections within four weeks prior to be included in the study; history of allergy to study drug components; had received treatment with any approved anti-cancer therapy within three weeks prior to initiation of study treatment or treatment with any other investigational agent with therapeutic intent within 28 days prior to initiation of study treatment; history of idiopathic pulmonary fibrosis, organizing pneumonia, drug-induced pneumonitis, idiopathic pneumonitis, or evidence of active pneumonitis on screening chest CT scan; had received therapeutic oral or intravenous antibiotics within two weeks prior to be included in the study.

Supplementary Procedures:

Adverse events and abnormal laboratory findings were graded according to the National Cancer Institute Common Terminology Criteria for Adverse Events (NCI-CTCAE) version 4.0. For laboratory parameters, only grade ≥2 events were reported as adverse events. All other adverse events not related to laboratory values were reported from grade 1.

Dose reductions were not permitted for either atezolizumab or bevacizumab in this study. Treatment could be temporarily interrupted, delayed, or discontinued depending on tolerability. Dose adjustment for bevacizumab was recommended if the patient's body weight changed by 10% or more during treatment. Treatment administration was resumed if protocol-defined criteria for treatment resumption were met (see trial protocol).

Withdrawal criteria included patient withdrawal of consent, unacceptable toxicity, non-compliance, intercurrent illnesses, or other reasons that the investigator deemed would substantially affect the patient’s safety.

Supplementary Molecular Methods:

PD-L1:

Formalin-fixed paraffin-embedded (FFPE) tissue samples were obtained from primary tumor or metastatic sites at pre-screening. PD-L1 expression was qualitatively assessed at each center according to clinical practice guidelines. PD-L1 protein expression was determined by using Tumor Proportion Score (TPS, percentage of viable tumor cells showing partial or complete

membrane staining at any intensity). A tissue sample was considered adequate for assay interpretation if it contained at least 50 viable tumor cells. PD-L1 TPS was categorized using different thresholds for statistical analysis (<1% vs ≥1%; <1% vs 1-49% vs ≥50%; <50% vs ≥50%).

Tumor mutational Burden (TMB):

Baseline TMB was assessed using the US Food and Drug Administration (FDA)-approved FoundationOne CDx assay (Foundation Medicine, Cambridge, MA, USA). Tissue samples were sent to a central laboratory (Foundation Medicine GmbH, Penzberg, Germany) in which tissue sections were analyzed by different methods including IHC and Hybrid-capture NGS based comprehensive genome sequencing by Foundation One technology. A minimum of 10 unstained slides cut at 4-5 microns thick (1 mm3), with at least 20% tumor cellularity was required (number of tumor cells divided by total number of all cells with nuclei). If the tissue submitted did not meet the criteria for successful testing and tissue biopsy was not feasible, TMB could be determined in a blood sample, after approval by the study chair/sponsor, using the FDA-approved FoundationOne Liquid CDx assay in a central laboratory (Foundation Medicine, Cambridge, MA, USA).

Blood samples:

Blood samples for the prespecified exploratory analyses of pharmacogenomic data were collected at the following time points: (i) baseline or before day 1 of cycle 1 (stool samples were also collected at baseline), (ii) first CT-scan evaluation (cycle 5), (iii) second CT-scan evaluation (cycle 9), (iv) fourth CT-scan evaluation (cycle 17), (v) sixth CT-scan evaluation (cycle 25) and at disease progression, either if it occurred during treatment with atezolizumab plus bevacizumab or during the follow up phase.

Hemoglobin (g/dL), Lymphocytes (x103/uL), Neutrophils (x103/uL), Platelets (x103/uL), and derived cell ratios (Neutrophil to lymphocyte ratio; NLR and Platelet to lymphocyte ratio; PLR), as well as Creatinine (mg/dl), Creatinine clearance (ml/min), Total Bilirubin (mg/dL), AST (IU/L), ALT (IU/L), ALP (IU/L), Calcium (mg/dl), Magnesium (mg/dL), Sodium (mmol/L), Potassium (mmol/L), LDH (IU/L), Glucose (mg/dl), Amylase (IU/L), Lipase (IU/L), Albumin (g/dL), and Total Protein (g/dL), were retrieved for identification of prognostic biomarkers from blood tests at baseline from each hospital.

Flow cytometry:

Peripheral blood samples (10mL) were diluted 1:1 in 1640 RPMI and mononuclear cells (PBMCs) were isolated by density gradient centrifugation using Lymphoprep (Stemcell, Vancouver, Canada) and cryopreserved in 1:1 RPMI/FBS media containing 10% DMSO (Carl Roth, Germany) until use. Cryopreserved PBMCs were thawed, washed with 5% FBS in 1x PBS, and surface stained with two antibody panels: First panel consisting of CD3-PerCP (clone BW264/56), CD8-APCVio770 (clone 135/80), CD4-Viogreen (clone REA623), CD19-FITC (clone LT19), CD127-PE (clone REA614), CD14-APC (clone Tük4) from Miltenyi Biotec (Germany), and CD25-PeCy7 (clone M-A251) from BD Bioscience (NJ, USA). Second panel consisting of CD3-PerCP (clone BW264/56), CD8-APCVio770 (clone 135/80), CD4-Viogreen (clone REA623), CD45RO-FITC (clone REA611), PD1-PEVio770 (clone PD1 3.1.3), CD62L-PE (clone REA615) from Miltenyi Biotec, and CD14-APC (clone Tük4) from Miltenyi Biotec (Germany). Also, samples were blocked with FcR

© 2022 Provencio M et al. *JAMA Oncology.*

Blocking Reagent (Miltenyi Biotec) and the Live/Dead Fixable Violet 405nm stain from ThermoFisher (MA, USA) was used to exclude dead cells in the analysis. Finally, samples were fixated with 1% PFA in 1x PBS before sample acquisition (MACS Quant 10 cytometer).

The main studied immune populations were B cells (CD3-CD14-CD19+), T cells (CD3+CD14-), cytotoxic T cells (CD3+CD14-CD4-CD8+) and helper T cells (CD3+CD14-CD4+CD8-) using FlowJo V10 software. The surface markers expression was evaluated by the median fluorescence intensity (MFI) and the percentage of positive cells using fluorescence minus one (FMO) controls. Flow cytometry was performed in 27 PBMCs samples from 19 patients: 19 samples obtained from patients with stable disease or partial response during treatment, and 8 at disease progression for paired analysis.

ctDNA:

Blood samples for ctDNA analysis were obtained at baseline, at the first, second, fifth, and seventh CT scans, and upon disease progression, and were sent to a central laboratory (Hospital Puerta de Hierro, Majadahonda-Madrid, Spain). In 16 cases, a plasma sample was available at all time points. Samples were collected in a 10mL Cell-Free DNA BCT (Streck). For plasma isolation, blood samples were centrifuged at 1600 x g for 10 min and immediately at 6000 x g for 10 min.

Cell-free DNA (cfDNA) was extracted using cfDNA QIAmp Circulating Nucleic Acid kit (Qiagen®) following the manufacturer's protocol. Libraries were prepared using the Oncomine™ Pan-Cancer Cell-Free Assay kit (Thermo Fisher, Palo Alto, CA, USA), according to the manufacturer´s instructions. For library purification, AMPureXP magnetic beads (Beckman Coulter, Inc., Brea, CA, USA) were used. Finally, libraries were diluted to 50 pM. 16 samples were sequenced in every NGS run using two Ion 550™ Chips, each of them loaded with eight pooled samples. Templating and Ion 550™ Chip loading were carried out with an Ion Chef™ System (Thermo Fisher, Palo Alto, CA, USA), then chips were sequenced on an Ion GeneStudio™ S5 Sequencer (Thermo Fisher, Palo Alto, CA, USA). Torrent Suite Software v5.12.2 was used to perform raw sequencing data analysis. The CoverageAnalysis v5.12.2 plugin was used for sequencing coverage analysis. Raw reads were aligned to the human reference genome hg19. Variant calling was carried out on the Ion Reporter platform v.5.18 using Oncomine TagSeq Pan-Cancer Liquid Biopsy – w2.5 – Single Sample (workflow versions from w2.1 to w2.5 were used). Variant filtering was performed using an internal pipeline (available from the corresponding author on reasonable request). All candidate mutations were manually reviewed using the Integrative Genomics Viewer (IGV) v.2.3.40, (Broad Institute, Cambridge, MA, USA). The sum of MAF from all of the variants detected for each sample was used to determine the amount of ctDNA.

Supplementary Endpoints:

Additional secondary endpoints included ORR, DOR, TTR, and PFS according to immune-related response criteria (irRC); PFS according to irRC; ORR, PFS and DOR according to irRC in patients with TMB ≥16; 2-year PFS and OS rate; patient-reported outcomes (PROs) of lung cancer symptoms, patient functioning, and health-related quality of life (HRQoL), as measured by the European Organisation for Research and treatment of Cancer (EORTC) Quality-of-life Questionnaire Core 30 (QLQ C30) and its Lung Cancer Module (QLQ LC13).

© 2022 Provencio M et al. *JAMA Oncology.*

Prespecified exploratory endpoints included determination of TMB and its association with all four types of genomic alterations (i.e., short insertions and deletions [indels], mutations, copy number variation [CNV] and rearrangements) in 324 tumor related genes, microsatellite instability (MSI), and other biomarkers such as tumor PD-L1 expression; to investigate changes in the landscape of genomic alterations in blood samples after disease progression relative to the landscape of genomic alterations determined in tissue samples, including MSI and TMB at progression;

Additional pharmacogenomic exploratory analysis include the basal peripheral blood T-cell receptor (TCR) repertoire and their changes during treatment, as well as their correlation with clinical variables associated with treatment efficacy (PFS, OS, ORR and DOR) and adverse events; analysis of pretreatment fecal microbiome in all patients included in the study and its predictive value in terms of PFS, OS, ORR and DOR.

© 2022 Provencio M et al. *JAMA Oncology.*

eResults.

Additional secondary (PFS and OS at 24months) and exploratory analysis (peripheral blood immune cells, soluble factors, and microbiome characterization) specified in protocol will be presented elsewhere.

Patient Characteristics:

From May 2019 through January 2021, a total of 307 patients were assessed for eligibility at 13 sites, with 266 ineligibles for enrolment. (149 TMB<10, 41 TMB≥10 but other non-eligible reasons are meet, 13 TMB cannot be determined, 24 no tumor or invalid sample, 21 Insufficient sample, 18 Other reasons). Of the 41 patients enrolled (intention-to-treat population, ITT), three patients (04600015, 02400023, 04600026) did not fulfill all enrollment criteria and were excluded. Patient 04600015 did not have measurable disease (inclusion criteria nº8); patient 02400023 was receiving clopidogrel (exclusion criteria nº37); and patient 04600026 was receiving azathioprine and had proteinuria (exclusion criteria nº31 and nº44).

Exploratory objectives:

PD-L1:

PD-L1 TPS was available in 30 cases (78.9%), showing a median PD-L1 TPS of 1% (IQR, 0-51.25%). 15 cases (50%) showed PD-L1 TPS <1%. Median PD-L1 TPS for patients with objective response was 20% (IQR, 0-90%, n=11) vs 0% (IQR, 0-13.5%, n=18) for patients with SD or PD (p=0.112), showing an ROC AUC of 0.679 (95%CI 0.466-0.892) (p=0.111). Finally, no differences in PFS, nor OS (post-hoc analysis) were observed (p=0.946 and p=0.250, respectively, using <1%, 1-49%, ≥50% PD-L1 TPS) (**Figure S2A**). ORR was not statistically significant associated to PD-L1 TPS subgroups using different thresholds (<1% vs ≥1%; <1% vs 1-49% vs ≥50%; <50% vs ≥50%). However, a trend for improved ORR across increasing PD-L1 TPS categories was observed (26,7% for <1%, 33,3% 1-49%, and 62,5% ≥50%, p=0.233) (**Figure S3A)**. No PFS differences between any PD-L1 subgroups using log-rank test were observed (p=0.880 for <1% vs ≥1%; p=0.946 for <1%, 1-49%, ≥50%; p=0.752 for <50% vs ≥50%) (**Figure S3B)**. Similarly, post-hoc analysis showed no differences for OS (p=0.111 for <1% vs ≥1%; p=0.250 for <1%, 1-49%, ≥50%; p=0.643 for <50% vs ≥50%) (**Figure S3C)**. Similarly, post-hoc analysis showed no differences for DOR or TTR using any PD-L1 categories (data not shown).

TMB:

TMB determined from tissue was available for all patients (n=38). Median TMB was 13 mut/Mb (IQR, 11-20). No association between TMB and PD-L1 levels was found (R=0.221, p=0.240) **(Figure S4)**. TMB was higher in patients with objective response, with a median TMB of 15.5 (IQR, 11.5-24.5) compared to 13 (IQR, 10.5-15.0) in patients with PD or SD (p=0.029), and showing a ROC AUC of 0.71 (95%CI, 0.539-0.880) for response prediction (p=0.031). TMB≥13 showed the best performance; however, no differences were observed in OS using this (p=0.830), or any other value, as cut-off. Similarly, post-hoc analysis showed no association of TMB categories with PFS (p=0.353), DOR (p=0.608), TTR (p=0.316). Median PFS for TMB<13

© 2022 Provencio M et al. *JAMA Oncology.*

group was 8.4 months (95%CI 6.4-10.4) vs 13.3 months (95%CI 10.3-16.4) in patients with TMB≥13 (p=0.353) (**Figure S2B**).

Role of Foundation One in detection of druggable mutations:

Of the 149 patients with low TMB, 56 (37.6%) presented at least one druggable alteration or driver mutation that might influence treatment selection; *KRAS* (G12C, 23 cases, 15.4%), *EGFR* (L858R+Exon19del+Exon20ins, 17 cases, 11.4%), *BRAF* (V600E, 7 cases, 4.7%), *HER2* (3 cases, 2.0%), *MET* (Exon 14 skipping, 2 cases, 1.3%), *ALK* fusion (2, 1.3%), *RET* fusion (2, 1.3%), and *ROS1* fusion (0, 0%). The proportions of patients harboring any druggable alterations, or *EGFR* alterations, were inferior in the 82 patients with TMB≥10, with only 14 cases with any alteration (17.1%, Fisher test p=0.001), and 1 case with *EGFR* (1.2%, p=0.004) (**Figure S5A**). Similarly, lower TMB levels were found in the group of screened patients with any druggable alteration (UMW test p=0.001), *EGFR* (p=0.001), *BRAF* (p=0.020) or *ALK* (p=0.038), compared to their wild-type counterparts **(Figure S5B).**

Mutations in per protocol population:

Post-hoc analysis showed no influence of mutations in *KRAS* (10 cases, 26.3%) or *P53* (31 cases, 81.6%) with responses, PFS or OS **(Figure S6).** However, a mutational signature of 5 genes (present in 12 cases, 31.6%) consisting on the presence of at least one mutation in *KEAP1* (4 cases, 10.5%), *RB1* (3 cases, 7.9%), *VEGFA* (2 cases, 5.3%), *PTEN* (2 cases, 5.3%), or *HER2* (2 cases, 5.3%), was associated to worse PFS (Median PFS 9.1; 95% CI 3.3-15.0 *vs* 16.5 months; 95% CI 4.7-28.4 in wild-type group; log-rank p=0.007) and OS (Median OS 13.0 months; 95% CI 5.3-20.6 *vs* not reached in wild-type group; log-rank p=0.048), but not with objective response (Fisher test p=1.00) (**Figure S7**). No mutations were found in *JAK1*, *JAK2*, *JAK3*, or *STAT3*.

Blood cell count and biochemistry:

None of the complete blood cell count and biochemistry variables at inclusion were associated with clinical response (data not shown). However, an association of higher basal levels of lactate dehydrogenase (LDH) (>333 IU/L) or alkaline phosphatase (ALP) (>147 IU/L) with worse PFS and OS was found. Median PFS and OS for patients with high LDH was 5.87 months (95%CI 0-14.08) and 7.00 months (95%CI 3.15-10.85), compared to 14.47 months (95% CI 11.76-17.17) and not reached, for patients with low LDH levels (p< 0.001 in both cases). Similarly, Median PFS and OS for patients with high ALP was 2.67 months (95%CI 0-7.27) and 5.50 months (95%CI 0-11.82), compared to 14.47 months (95% CI 12.45-16.48) and not reached, respectively, for patients with low ALP levels (p< 0.001 for PFS, p=0.003 for OS). (**Figure S8**).

Peripheral blood immunophenotyping:

PBMCs samples were obtained from 19 patients with stable disease or partial response during treatment and analyzed by flow cytometry to identify possible markers associated with disease progression. (**Figure S9**). At data cutoff, from these 19 patients, 11 had disease progression and 3 had died, limiting overall survival analysis. Percentage of subpopulations and MFIs for different cell markers were categorized in high or low levels according to cohort median values.

Patients with higher PD-1 positive T cells (% CD3+CD14-PD1+) or low CD25 positive B cells (% CD3-CD14-CD19+CD25+) percentages showed worse PFS, with a HR (hazard ratio) for disease progression of 4.08 (95% CI 1.29-13.68; p=0.031) and 4.43 (95% CI 1.19-16.45; p=0.006),

© 2022 Provencio M et al. *JAMA Oncology.*

respectively (**Figure S9A**). Similarly, patients with higher MFI levels of CD4 in helper T cells (CD3+CD14-CD8-CD4+) or CD62L in monocytes (CD3-CD14+) showed poorer PFS, with a HR for disease progression of 4.08 (95% CI 1.29-13.68; p=0.019) and 3.86 (95% CI 1.16-12.85; p=0.029), respectively (**Figure S9B**).

Finally, differences between paired samples during treatment and at disease progression were determined from 8 patients (**Figure S10**). In the analysis of B cells, we found that patients at disease progression showed lower median values of CD19 MFI (1931 [IQR 1469–2227]) compared to samples obtained during disease control (2283 [IQR 1928–2807]) (p value=0.039). Conversely, patients at disease progression showed higher percentage of B cells (CD3-CD14-CD19+) (3.06% [IQR 1.96%-3.78%] compared to samples during disease control (1.76% [1.62%-2.40%]) (p value=0.023) (**Figure S10A**).

Regarding T cells, patients at disease progression showed lower median values of CD3 MFI in T cells, reaching statistical significance for the CD8+ cytotoxic subpopulation (CD3+CD14-CD4-CD8+) (2111 [IQR 1843–2317] vs 1993 [IQR 1661–2191], p value=0.039). Additionally, cytotoxic T cells showed a trend for lower CD8 MFI at disease progression that was not statistically significant. Further subclassification into CD8 high or CD8 low cytotoxic T cells showed opposite behavior for CD8 MFI, with significantly increased levels in samples at disease progression compared to samples during disease control for CD8 low subpopulation (794 [IQR 765–888] vs 728 [IQR 699–796], p value=0.031) (**Figure S10B**).

ctDNA analysis:

In 16 cases, at least two plasma samples were available for ctDNA analysis during the course of treatment. Among patients in whom ctDNA decrease during treatment or became undetectable (n=4) (below the limit of detection established at 0.1% MAF) progressive disease was diagnosed in one case (25%) and none of them were deceased. On the other hand, in patients in whom ctDNA increased (n=12), disease progression was diagnosed in 8 (67%) of them and three of them were deceased. However, these differences were not statistically significant, probably due to the small sample size. Kaplan Meier curves for PFS and OS and according to ctDNA fluctuations are depicted on **Figure S11**.

Cox proportional-hazards survival analysis:

Univariate Cox proportional-hazards of clinical and molecular variables were carried out for progression-free and overall survival **Figure S12**. Results shown that having stage IVB, elevated ALP, or elevated LDH was statistically significant associated to higher risk of disease progression or death. Additionally, having ECOG 1 was associated to higher risk of death. PD-L1 and TMB showed no statistically significant impact in PFS nor OS in multivariate analysis after adjustment by different co-variables (data not shown).

© 2022 Provencio M et al. *JAMA Oncology.*

eFigure 1. Disposition of Patients in the Study

Supplementary Figure S1.

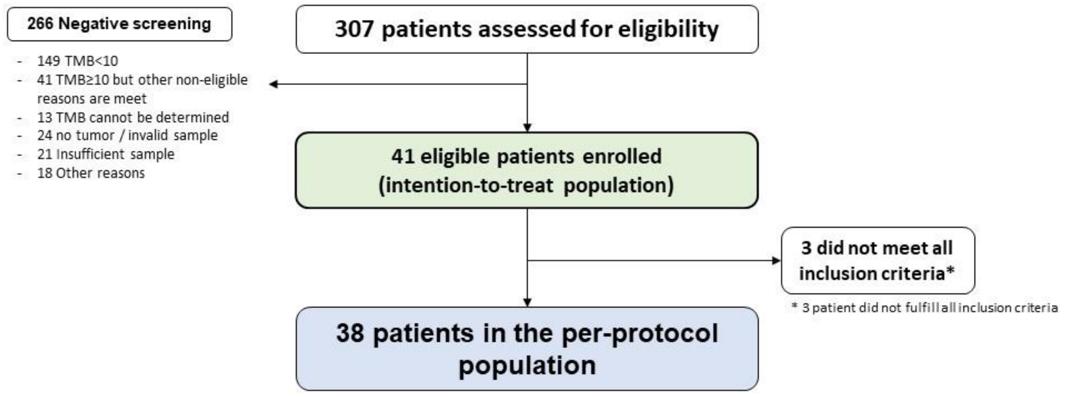

© 2022 Provencio M et al. *JAMA Oncology.*

eFigure 2. PD-L1 and TMB Biomarkers

Association of PD-L1 TPS and response (n=29) (left panel) or survival; progression-free survival or overall survival (n=30) (right panels) (A). Association of TMB and response (n=37) (left panel) or survival; progression-free survival or overall survival (n=38) (right panels) (B).

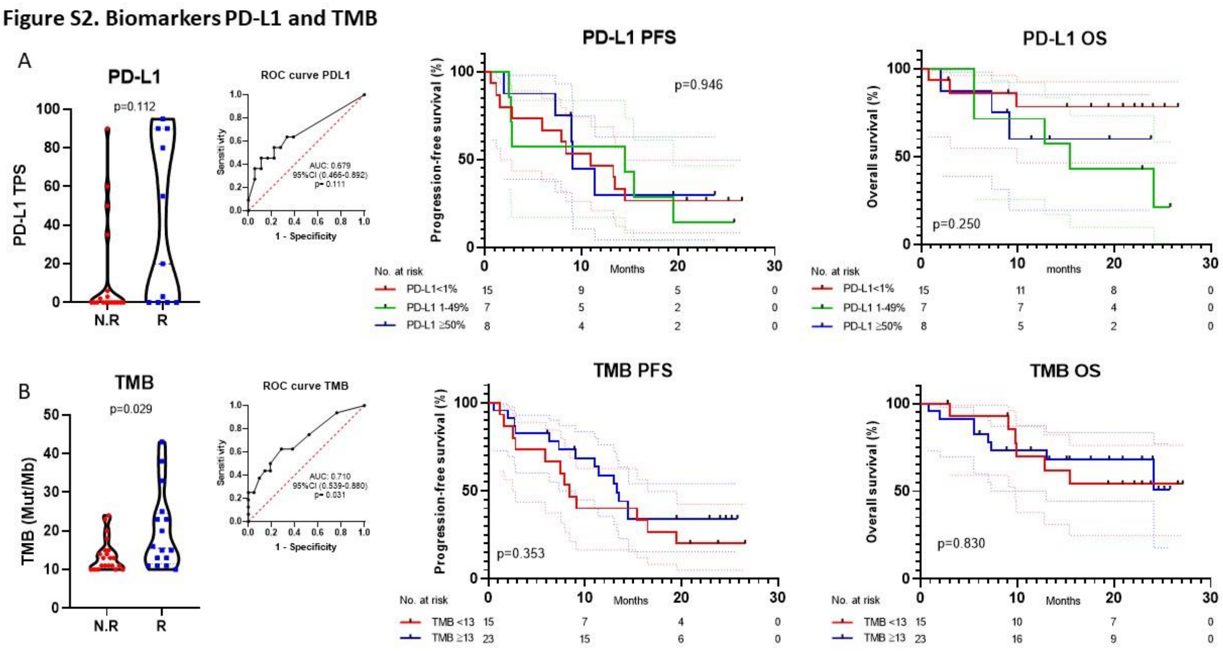

© 2022 Provencio M et al. *JAMA Oncology.*

eFigure 3. PD-L1 Categories and ORR

(A) Distribution of patients according to PD-L1 categories and ORR. Kaplan-Meier curves for progression-free survival (PFS) (B) and overall survival (OS) (C) in patients according to PD-L1 categories (<1% vs ≥1% and <50% vs ≥50%).

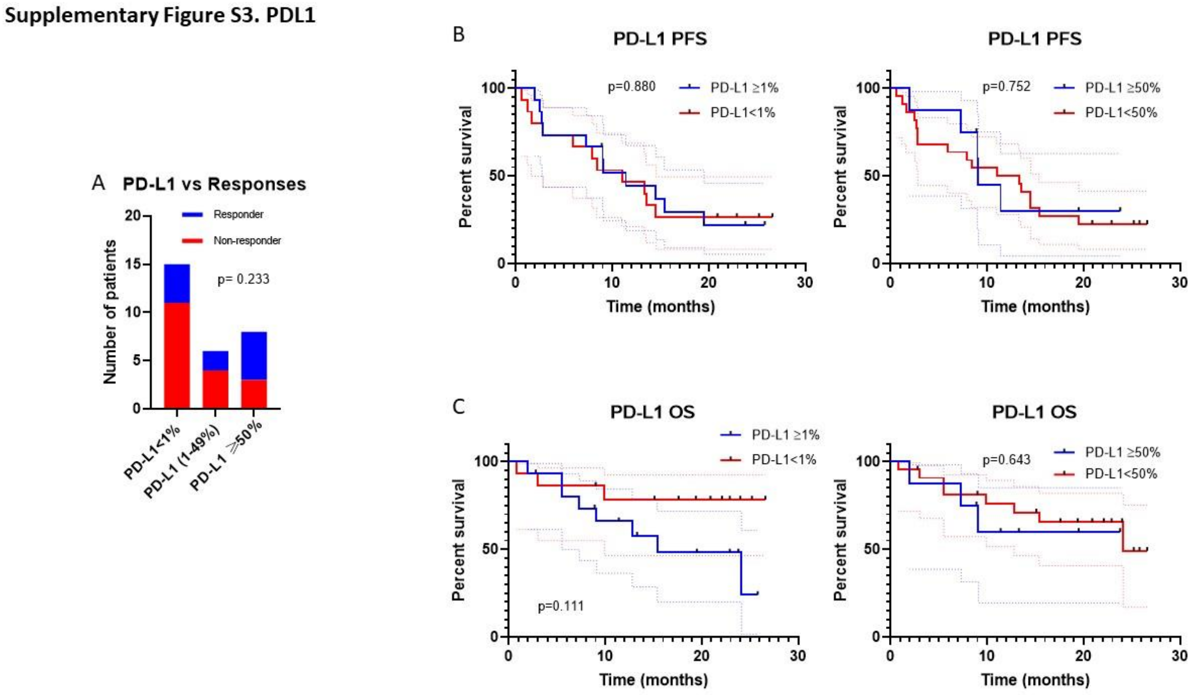

© 2022 Provencio M et al. *JAMA Oncology.*

eFigure 4. TMB and PD-L1 Correlation

No association between TMB and PD-L1 levels was found (R=0.221, p=0.240).

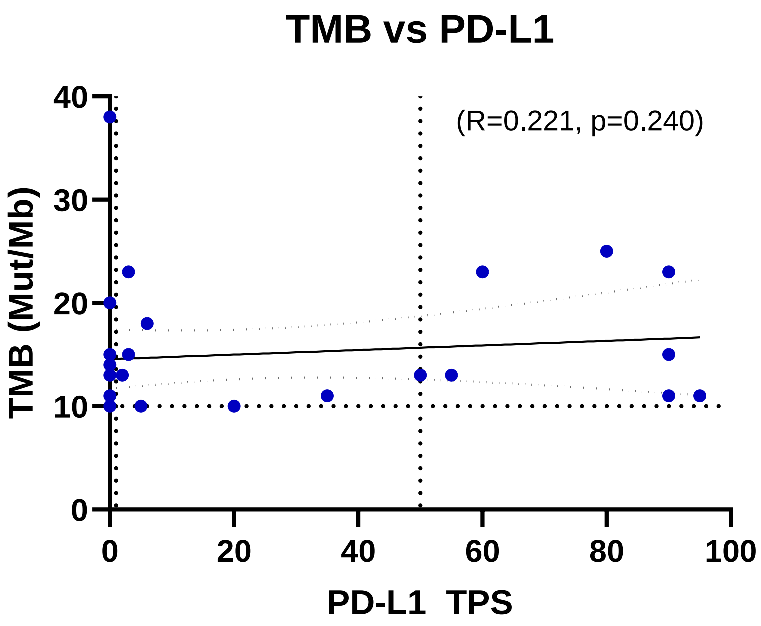

© 2022 Provencio M et al. *JAMA Oncology.*

eFigure 5. TMB and Druggable Alterations in All Screened Patients

(A) Number of cases with druggable alterations in TMB <10 and TMB>10 subgroups of patients.

(B) TMB levels according to gene status for any druggable alteration, *EGFR*, *BRAF*, and *ALK*.

Supplementary Figure S5. TMB and druggable alterations in all screened patients

A

	TMB<10 (n=149)		TMB≥10 (n=82)		
	Cases	Percentage	Cases	Percentage	p-value
Any alteration	56	37,6%	14	17,1%	0.001
KRAS_G12C	23	15,4%	11	13,4%	0.846
EGFR_	17	11,4%	1	1,2%	0.004
BRAF_V600E	7	4,7%	1	1,2%	0.265
HER2_	3	2,0%	3	3,7%	0.669
MET_exon14skip	2	1,3%	0	0,0%	0.540
EML4-ALK fusion	2	1,3%	0	0,0%	0.540
KIF5B-RET fusion	2	1,3%	0	0,0%	0.540
EZR-ROS1 fusion	0	0,0%	0	0,0%	1.00

B

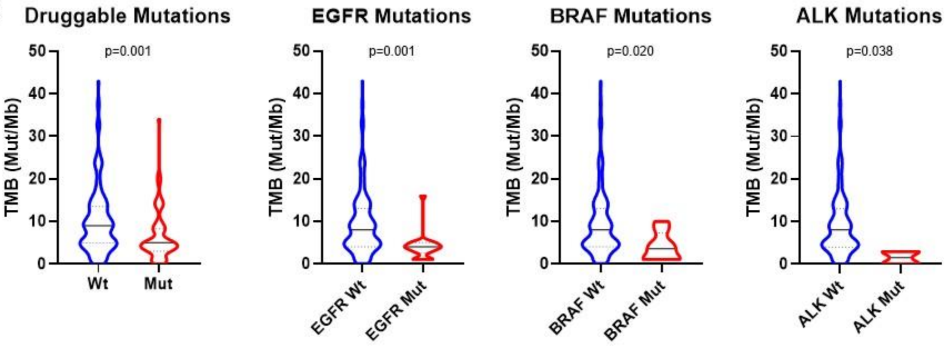

© 2022 Provencio M et al. *JAMA Oncology.*

eFigure 6. High Prevalence Sequence Variations (*KRAS* or *P53*) and Outcomes (ORR and PFS-OS)

(A) Distribution of patients according to *KRAS* or *TP53* mutational status and ORR. Kaplan-Meier curves for progression-free survival (PFS) (B) and overall survival (OS) (C) in patients according to KRAS or TP53 mutational status.

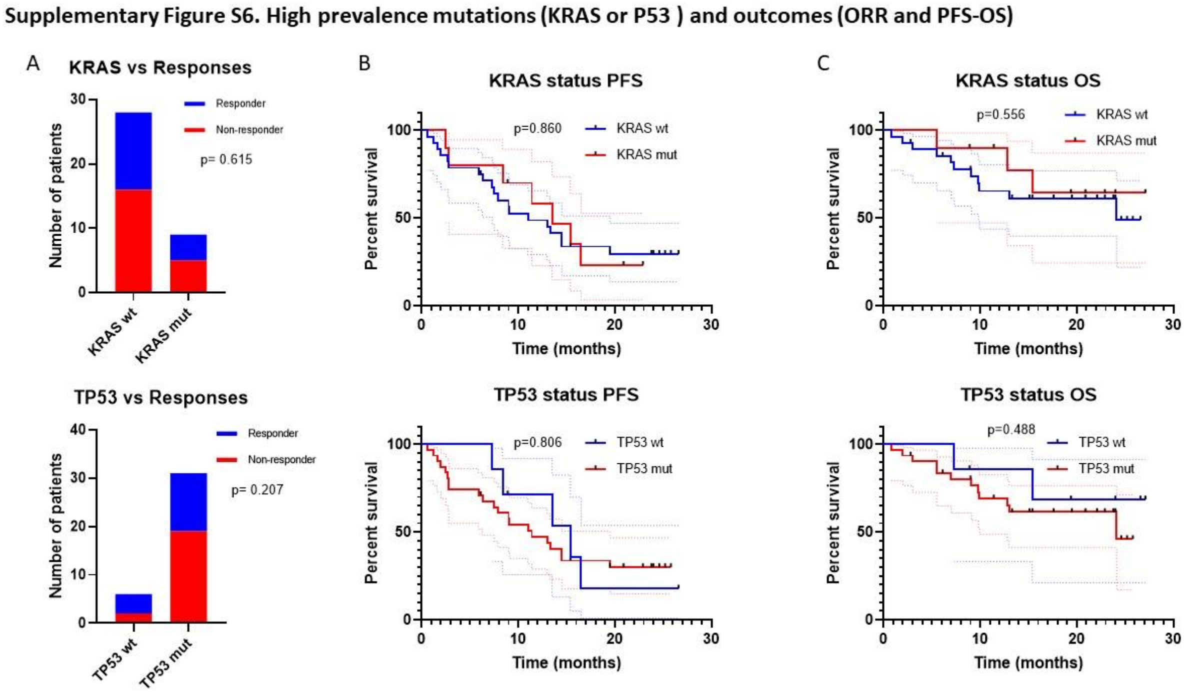

© 2022 Provencio M et al. *JAMA Oncology.*

eFigure 7. Sequence Variation Signature and Outcomes (ORR and PFS-OS)

Mutational signature of 5 genes (present in 12 cases, 31.6%) consisting on the presence of at least one mutation in *KEAP1* (4 cases, 10.5%), *RB1* (3 cases, 7.9%), *VEGFA* (2 cases, 5.3%), *PTEN* (2 cases, 5.3%), or *HER2* (2 cases, 5.3%), was associated to worse PFS and OS, but not with objective response.

Supplementary Figure S7. Mutational signature and outcomes (ORR and PFS-OS)

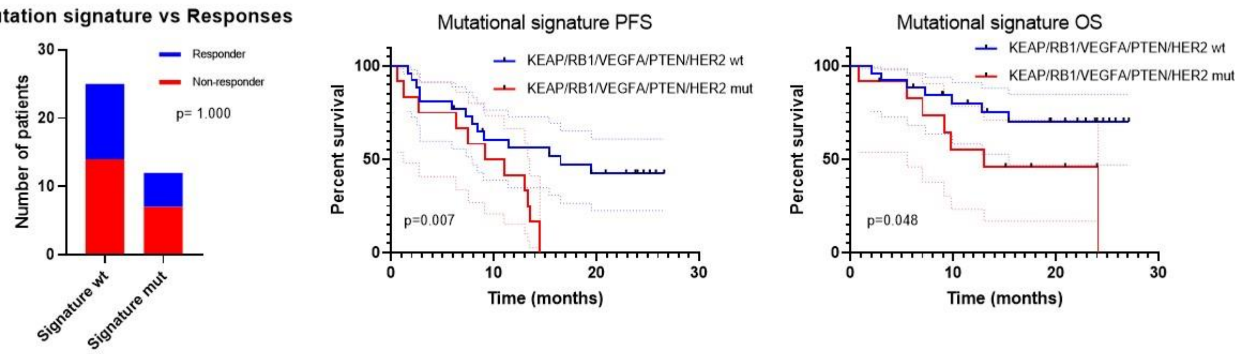

© 2022 Provencio M et al. *JAMA Oncology.*

eFigure 8. Blood Counts and Biochemistry With Outcomes (ORR and PFS-OS)

Distribution of patients according to elevated plasma levels of LDH (>333 IU/L) or ALP (>147 IU/L) and ORR. Kaplan-Meier curves for progression-free survival (PFS) and overall survival (OS) in patients according to LDH or ALP plasma levels.

Supplementary Figure S8. Blood counts and Biochemistry and outcomes (ORR and PFS-OS

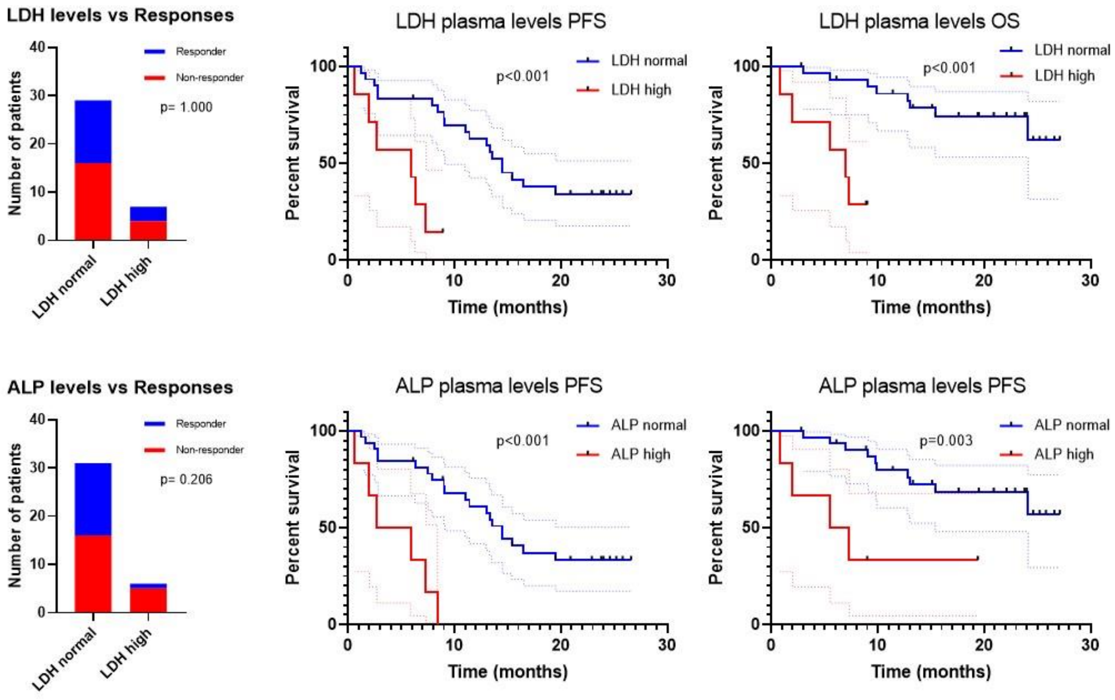

© 2022 Provencio M et al. *JAMA Oncology.*

eFigure 9. Flow Cytometry Analysis of Samples During Treatment and PFS

(A) Kaplan-Meier curves for progression-free survival (PFS) according to percentage of T cells PD-1+ (% CD3+ CD14-PD-1+) and B cells CD25+ (CD3-CD14-CD19+CD25+) and (B) to CD4 MFI of helper T cells (CD3+CD14-CD8-CD4+) and CD62L MFI of monocytes CD62L+ (CD3-CD14+CD62L+). High or low categories were determined using cohort median values.

Supplementary Figure S9. Flow cytometry analysis of samples during treatment and PFS.

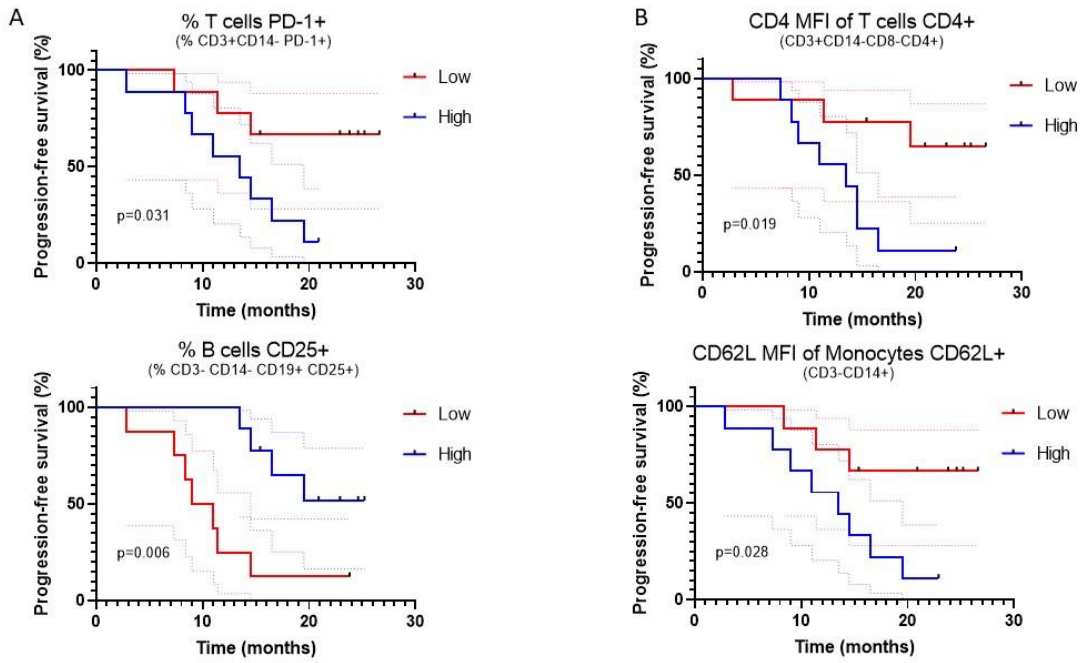

© 2022 Provencio M et al. *JAMA Oncology.*

eFigure 10. Flow Cytometry Analysis of Paired Response and Progression Samples

(A) Median fluorescence intensity (MFI) of CD19 on B lymphocytes (CD3-CD14-CD19+) (upper panel) and percentage of B lymphocytes (CD3-CD14-CD19+) (lower panel) (B) MFI of CD3 on T lymphocytes (CD3+CD14-), CD8+ T lymphocytes (CD3+CD14-CD4-CD8+) and CD4+ T lymphocytes (CD3+CD14-CD4+CD8-) and MFI of CD8 on the total of CD8+ T lymphocytes (CD3+CD14-CD4-CD8+), and the subpopulations of CD8high T lymphocytes (CD3+CD14-CD4-CD8high) and CD8low T lymphocytes (CD3+CD14-CD4-CD8low).

Supplementary Figure S10. Flow cytometry analysis of paired samples during disease control and progression.

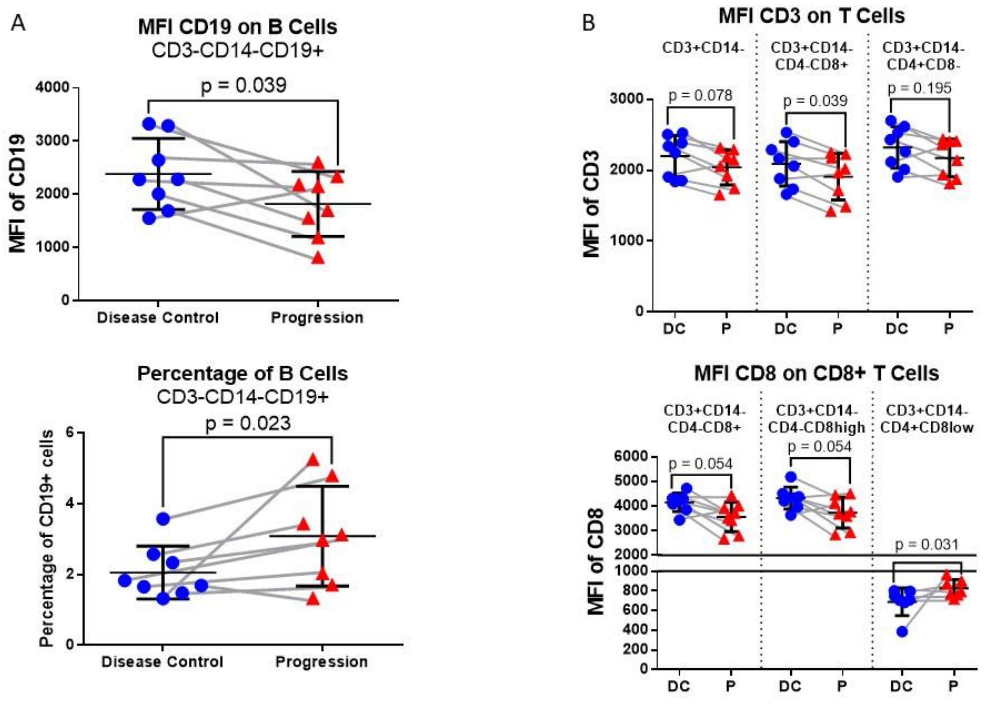

© 2022 Provencio M et al. *JAMA Oncology.*

eFigure 11. ctDNA Follow-up

Kaplan-Meier curves for progression-free survival (PFS) and overall survival (OS) in patients who became ctDNA-negative or in whom ctDNA decrease over the course of treatment (n=4, Blue line, ctDNA decrease) and patients who did not (n=12, Red line, ctDNA increase).

Supplementary Figure S11. ctDNA follow up.

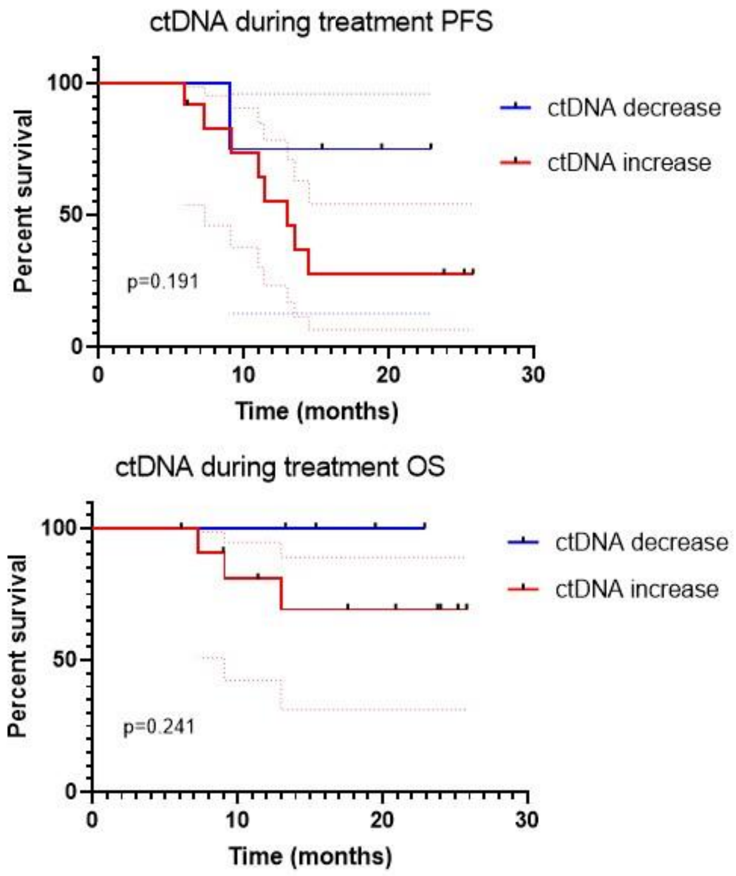

© 2022 Provencio M et al. *JAMA Oncology.*

eFigure 12. Impact of Clinical and Molecular Variables on PFS and OS

Univariate Cox proportional-hazards of clinical and molecular variables were carried out for progression-free and overall survival. Having stage IVB, elevated ALP, or elevated LDH was statistically significant associated to higher risk of disease progression and death. Additionally, having ECOG 1 was associated to higher risk of death. Hazard ratios and corresponding 95% confidence interval are shown for each variable. Categorical variables: Sex (Female vs Male), Smoking (Former vs current smoker), ECOG-PS (1 vs 0), Histology (Large Cell or NOS vs Adenocarcinoma), TMB (TMB≥13 vs TMB<13), ALP (alkaline phosphatase) (ALP>147 vs ALP≤147), LDH (lactate dehydrogenase) (LDH>333 vs LDH≤333), PD-L1 (PD-L1 TPS≥1% vs PD-L1 TPS<1%).

Supplementary Figure S12. Impact of clinical and molecular variables on PFS and OS.

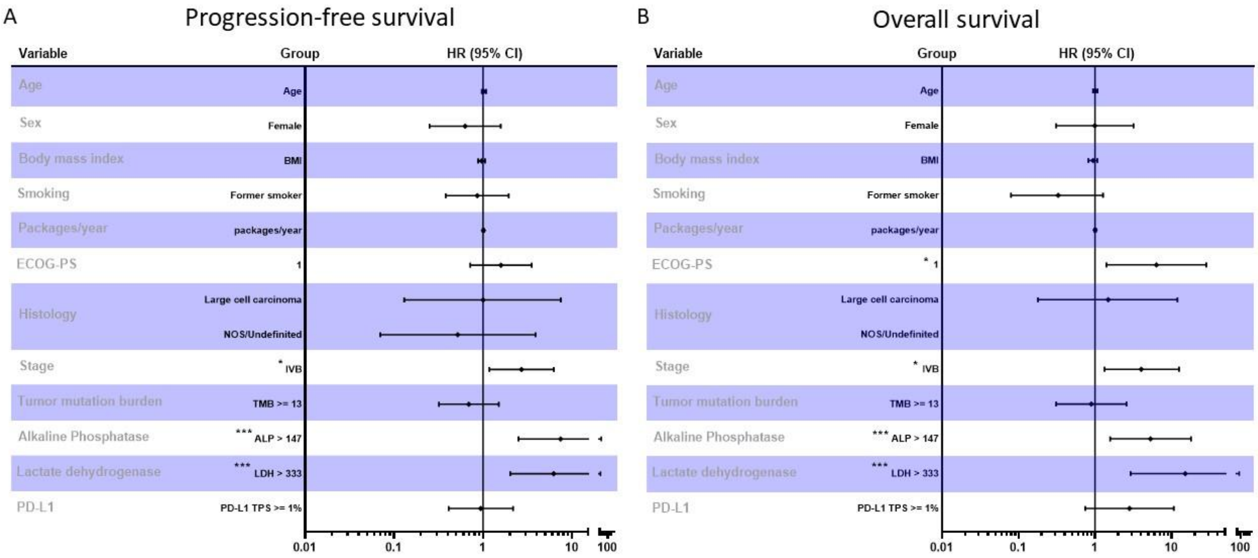

Variable	HR (PFS)	95% CI	p-value
ECOG	1.59	(0.72-3.52)	0.25
Stage	2.70	(1.18-6.25)	0.019
ALP	7.43	(2.50-22.11)	<0.001
LDH	6.21	(2.01-19.14)	0.003

Variable	HR (OS)	95% CI	p-value
ECOG	6.33	(1.41-28.40)	0.016
Stage	4.00	(1.33-12.50)	0.014
ALP	5.30	(1.57-17.88)	0.007
LDH	15.10	(2.91-78.44)	0.001

© 2022 Provencio M et al. *JAMA Oncology.*

eTable 1. Associated Comorbidities (Per-Protocol Population)

Comorbidity	No. (%)
Hypertension	19 (50)
Dyslipemia	17 (44.7)
COPD	12 (31.6)
Diabetes mellitus	11 (28.9)
Benign prostatic hypertrophy	5 (13.2)
Depressive syndrome anxiety	5 (13.2)
Heart disease	4 (10.5)
Alcoholism	2 (5.3)
Hepatitis	2 (5.3)
Vasculopathy	2 (5.3)
Asthma	1 (2.6)
Hypercholesterolemia	1 (2.6)
Obesity	1 (2.6)
Arthritis	0 (0)
Autoimmune disease	0 (0)
Hyperthyroidism	0 (0)
Hypothyroidism	0 (0)
Nephropathy	0 (0)
Neurological disease	0 (0)
Osteoporosis	0 (0)
Tuberculosis	0 (0)

COPD, chronic obstructive pulmonary disease.

© 2022 Provencio M et al. *JAMA Oncology.*

eTable 2. Treatment Compliance

**Atezolizumab**				
		**AEs grade 1**	**AEs grade 2**	**AEs grade 3**
Treatment discontinuation	4 (10.5)			
Cause: Adverse events	2 (5.3)	0 (0.0)	0 (0.0)	2 (5.3)
Cause: Missing	2 (5.3)	—	—	—
Treatment delays	15 (39.5)			
Cause: Adverse events	9 (23.7)	1 (2.6)	4 (10.5)	4 (10.5)
Cause: Other	5 (13.2)	—	—	—
Cause: Missing	1 (2.6)	—	—	—
Dose omission	6 (15.8)			
Cause: Adverse events	3 (7.9)			3 (7.9)
Cause: Other	0 (0.0)	—	—	—
Cause: Missing	3 (7.9)	—	—	—

**Bevacizumab**				
		**AEs grade 1**	**AEs grade 2**	**AEs grade 3**
Treatment discontinuation	4 (10.5)			
Cause: Adverse events	3 (7.9)	0 (0.0)	1 (2.6)	2 (5.3)
Cause: Missing	1 (2.6)	—	—	—
Treatment delays	14 (31,8%)			
Cause: Adverse events	9 (23.7)	2 (5.3)	3 (7.9)	4 (10.5)
Cause: Other	4 (10.5)	—	—	—
Cause: Missing	1 (2.6)	—	—	—
Dose omission	12 (31.6)			
Cause: Adverse events	10 (26.3)	2 (5.3)	2 (5.3)	6 (15.8)
Cause: Other	1 (2.6)	—	—	—
Cause: Missing	1 (2.6)	—	—	—

© 2022 Provencio M et al. *JAMA Oncology.*
