# Supplementary material for: Atezolizumab Plus Bevacizumab as First-line Treatment for Patients With Metastatic Nonsquamous Non–Small Cell Lung Cancer With High Tumor Mutation Burden: A Nonrandomized Controlled Trial
Source: JAMA Oncol. 2022 Dec 15;9(3):344–53. doi: 10.1001/jamaoncol.2022.5959 (PMC9856905; doi:10.1001/jamaoncol.2022.5959)
Supplement: Supplement 3. — Data Sharing Statement [file jamaoncol-e225959-s003.pdf]

Data Sharing Statement

Provencio. Atezolizumab Plus Bevacizumab as First-Line Treatment for Patients With Metastatic Nonsquamous Non-Small Cell Lung Cancer With High Tumor Mutation Burden. *JAMA Oncol*. Published December 15, 2022. doi:10.1001/jamaoncol.2022.5959

Data
Data available: Yes
Data types: Deidentified participant data
How to access data: mprovenciop@gmail.com
When available: With publication

Supporting Documents
Document types: None

Additional Information
Who can access the data: researchers whose proposed use of the data has been approved
Types of analyses: Academic purpose
Mechanisms of data availability: after approval of a proposal
